# Mass Spectrometry-Based Metabolomics Analysis of Obese Patients’ Blood Plasma

**DOI:** 10.3390/ijms21020568

**Published:** 2020-01-15

**Authors:** Petr G. Lokhov, Elena E. Balashova, Oxana P. Trifonova, Dmitry L. Maslov, Elena A. Ponomarenko, Alexander I. Archakov

**Affiliations:** 1 Institute of Biomedical Chemistry, 10 Building 8, Pogodinskaya Street, 119121 Moscow, Russia; balashlen@mail.ru (E.E.B.); oxana.trifonova@gmail.com (O.P.T.); dlmaslov@mail.ru (D.L.M.); 2463731@gmail.com (E.A.P.); alexander.archakov@ibmc.msk.ru (A.I.A.); 2 Metabometrics Inc, 651 N Broad St, Suite 205 #1370, Middletown, DE 19709, USA

**Keywords:** metabolomics, obesity, mass spectrometry, blood plasma, metabolite set enrichment analysis, biological context, metabolite identification, putatively annotated metabolites

## Abstract

Scientists currently use only a small portion of the information contained in the blood metabolome. The identification of metabolites is a huge challenge because only highly abundant and well-separated compounds can be easily identified in complex samples. However, new approaches that enhance the identification of compounds have emerged; among them, the identification of compounds based on their involvement in a particular biological context is a recent development. In this work, this approach was first applied to identify metabolites in complex samples and, together with metabolite set enrichment analysis, was used for the evaluation of blood plasma from obese patients. The proposed approach was found to provide a statistically sound overview of the biochemical pathways, thus presenting additional information on obesity. Obesity progression was demonstrated to be accompanied by marked alterations in steroidogenesis, androstenedione metabolism, and androgen and estrogen metabolism. The findings of this study suggest that the workflow used for blood analysis is sufficient to demonstrate obesity at the biochemical pathway level as well as to monitor the response to treatment. This workflow is also expected to be suitable for studying other metabolic diseases.

## 1. Introduction

Metabolomics belongs to the “omics” sciences and has emerged as a result of the evolution of measurement technologies. Modern scientific instruments can measure nearly complete sets of compounds in a sample. This type of research allows performing large-scale studies of nucleic acids in genomics, proteins in proteomics, and low-molecular-weight compounds (metabolites) in metabolomics. 

At the molecular level, life is a biochemical process consisting of hundreds of biochemical reactions; as a result, some compounds convert to others, a building material (amino acids and lipids) appears, and energy is produced for the organism. Since low-molecular-weight compounds (metabolites) are involved in all biochemical reactions as substrates, intermediates, and products, any process that occurs in the body is reflected in the metabolome—the total set of these compounds. By measuring the concentration of these compounds, the state of the organism can be defined, and dysfunction in the organism can be defined at the molecular level [1]. 

Generally, mass spectrometry techniques involved in metabolomics studies allow for the detection of hundreds of compounds, which is crucial for attaining biochemical knowledge. Unfortunately, no complete identification of compounds in any organismal metabolome has been reported to date [2]. Even if the most advanced metabolomics technologies using mass spectrometry are applied, an overwhelming majority of the compounds in the sample remain unknown [3]. The reason for this is that well-detected compounds are identified, which are usually the most abundant in the sample and are well-separated during the measurement. This is due to the fact that the mass spectrometry fingerprint of the metabolite fragments must be compared with the fingerprint from the database or with the fragmentation pattern of the chemical standard of the compound, which requires both a high-intensity mass spectrometry peak of the identified compound and high-quality isolation from other substances. The situation is complicated by the fact that the compounds are detected in different forms (adducts with different ions of salts and solvents), they are subjected to fragmentation during measurement (and fragments can also give different adducts), and many compounds are catabolized in the body, leading to the formation of many derivatives. As a result, in metabolomics studies, too many detected compounds remain unidentified, and more complete identification of compounds requires significant effort.

Thus, there is a problem: while metabolomics allows for the measurement of large sets of compounds and effectively describes an organism’s state at the molecular level, metabolomics methods are still weak with respect to compound identification. This has led to the emergence of new approaches to improve the identification of compounds; among them, the annotation (i.e., low probability identification) of compounds based on their involvement in a particular biological context has been developed. The aim of this study was to apply such an approach, which was previously introduced by Rogers and coworkers [4] and further advanced by Silva and coworkers [5], where the probabilistic algorithm is used for the quick annotation of low-molecular-weight compounds in the mass spectra. This algorithm takes experimental data and biochemical reaction database information to calculate the likelihood function, allowing compounds to be annotated. This approach was first applied to study blood samples from obese patients in order to test the approach on complex organisms (e.g., human) and complex samples (e.g., blood), as well as to determine its capacity to reveal additional insights of obesity at the molecular level. 

Obesity has become one of the major public health problems worldwide [6]. Obesity is a critical risk factor for cardiovascular diseases, insulin resistance and type 2 diabetes, hypertension, gallstone disease, and metabolic syndrome [7]. Furthermore, the link between obstructive sleep apnea and obesity has been recognized for a long time [8]. Reviews of available epidemiological data reiterate the existence of a significant association between obesity and increased risk for several cancers [9]. With the epidemic proportions achieved by the prevalence of obesity worldwide, it is crucial that we fully understand the factors that can control the development of this disease. 

## 2. Results

### 2.1. Mass Spectrometry Measurement of Compounds in Blood

**Figure 1:**
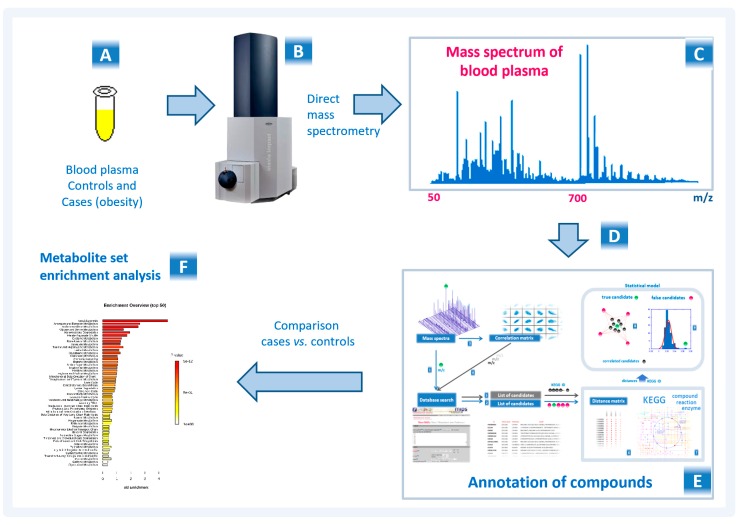
The workflow used to analyze the blood plasma samples from subjects involved in this study. Blood plasma samples (**A**) are collected and transported to the laboratory. In the laboratory, using ultrahigh-resolution mass spectrometry (**B**), the mass spectrum of blood plasma is obtained (**C**). The obtained mass spectra after preprocessing (alignment and normalization) (**D**) are analyzed according to a compound annotation algorithm (**E**), and the retrieved results are used in the metabolite set enrichment analysis (MSEA) (**F**).

Mass spectrometry of blood plasma generated typical mass spectra of the low-molecular-weight fraction of blood (Figure 1C). On average, 9333 peaks were detected in the spectrum (Table 1). The mass peaks of compounds were submitted to the metabolite search engine MASSTrix to annotate the compounds matching the mass peaks; 26,430 records of compound names with an associated *m*/*z* value were retrieved. 

### 2.2. Compound Annotation Algorithm

From the 26,430 records of compound names associated with *m*/*z* values, the annotation algorithm selected 2015 candidates as a putatively true annotation. The names of 41 xenobiotics were presented among the annotations. Due to the nature of the annotation algorithm, they were considered as unreliable annotations and were excluded from the results. Finally, application of the annotation algorithm resulted in the annotation of 386 compounds (Table 1). Among the annotated compounds, nearly all of the well-known and clinically relevant metabolites were present: amino acids, hormones, fatty acids, nucleotides, carbohydrates, cholesterol and its derivatives, etc.

### 2.3. Metabolite Set Enrichment Analysis (MSEA)

MSEA revealed alterations in steroidogenesis, androstenedione metabolism, and the metabolism of androgens and estrogens in patients who were overweight or at various stages of obesity (Table 2).

**Figure 2:**
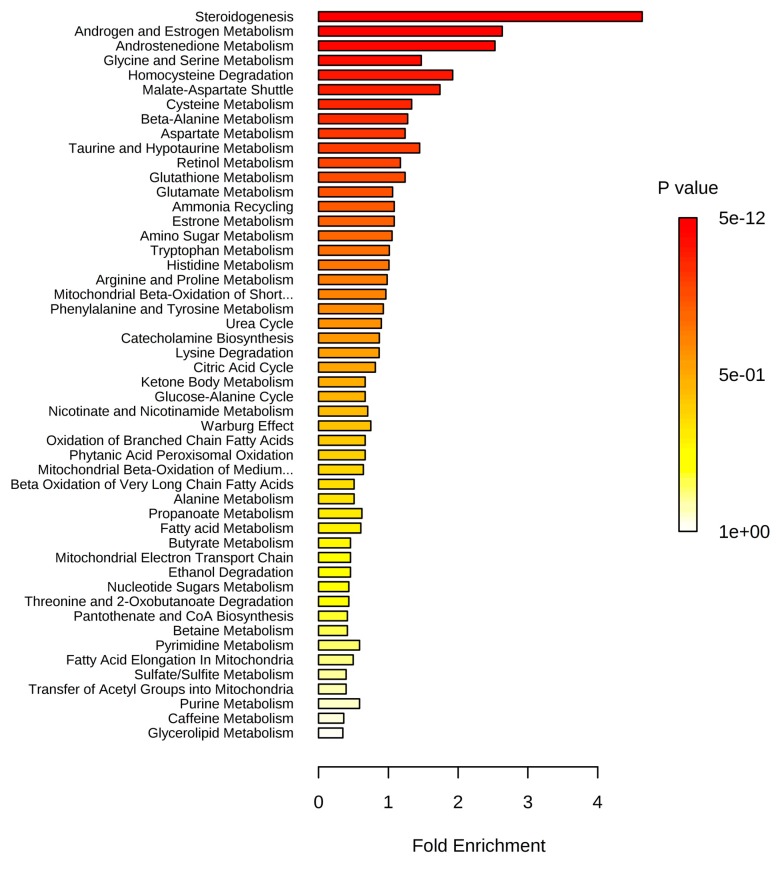
Summary plot for over-representation analysis (ORA) of blood plasma metabolites with an abnormal concentration in obese patients (stage 3). Information on the total number of metabolites in the pathways and other statistics are presented in Table S6.

It is noteworthy that an alteration in the metabolism of androgens and estrogens was clearly detected in overweight as well as obese individuals. Moreover, alteration of steroidogenesis increased with the stage of obesity (Figure 2) and appeared as a decrease of metabolite concentrations. 

**Figure 3:**
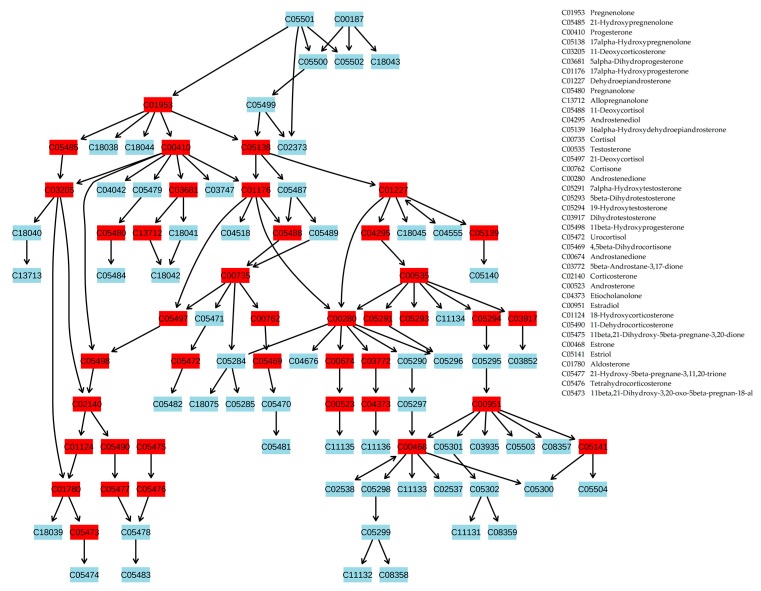
Projection of blood plasma metabolites with an abnormal concentration in patients with stage 3 obesity on the steroid hormone biosynthesis pathway. Metabolites are labeled by KEGG identifiers. Altered metabolites are marked by red boxes. This figure shows that almost all steroid hormone biosynthesis is altered in obese patients. The image was generated using MetaboAnalyst software. The names of the altered metabolites are presented in the figure. Statistical data for the altered metabolites are presented in Table S2. The same data, separately for men and women, are presented in Figures S13 and S14.

A review of the male and female groups, separately, revealed that these changes begin earlier and are much more pronounced in the female group (from stage 1 obesity in females compared to stage 2–3 obesity in males). In addition, less pronounced changes were found in the urea cycle (stage 1 obesity in males; overweight and stage 1 obesity in females) and aspartate metabolism (stage 1 obesity in females) pathways in the female group. The most pronounced metabolic changes associated with steroid hormone biosynthesis were projected onto the metabolic pathway (Figure 3), which showed that almost all steroidogenesis was altered in obese patients.

## 3. Discussion

Metabolomics is a rapidly emerging science, and current advances in mass spectrometry resolution and sensitivity make metabolomics methods attractive for scientific studies. However, there is still a bottleneck to overcome before metabolomics measurements can be accepted as a comprehensive way to study metabolites. Usually, only the most abundant and well-separated metabolites in the sample can be annotated. The reason for this is that the most popular method to annotate compounds is via the detection of fragmentation patterns, and a clearly separated and intense signal (mass peak) from the annotated metabolite is required.

To address this bottleneck, a new compound annotation approach was recently developed [5]. This algorithm uses metabolic pathway data and allows for the effective analysis of low-molecular-weight blood components (metabolome) with relatively high speed. Unfortunately, food intake and blood clearance result in fluctuating compound levels in the blood that smooth the connections between compounds established in pathways. In addition, blood is a collector of compounds released by different types of cells with their own biochemical reaction profile, making the connection between the concentration of compounds in the blood more complex. Therefore, samples of cell and tissue fragments are the most suitable for the application of the proposed approach because they do not have any of the abovementioned shortcomings of bodily fluids. It is no coincidence that this approach was tested previously only on simple organisms (*Trypanosoma brucei* and *Saccharum officinarum*), in which the composition of the substances is consistent with metabolic pathways. However, we hypothesized that in the case of metabolomic disorders, this approach should work. Cascade alterations in the metabolic pathway may be reflected in blood plasma, allowing the application of the metabolite annotation algorithm based on the biological context. Starting from this point, we applied this approach to study obesity.

While the implementation of a compound annotation algorithm appears relatively complex, the basic principle is quite simple. The first step is the selection of compound names by an accurate mass tag. This step is realized by high-resolution mass spectrometry and results in lists of compound names associated with a measured molecular weight. In this study, many candidates on average were associated with one mass. The main tasks of the algorithm are to compare the obtained experimental data, i.e., mass spectra, with the available information on biochemical pathways and to decline all false candidates. It is known that the concentrations of compounds involved in the same pathways correlate [10]. Thus, if we have mass spectrometric data for a set of samples, we can determine which mass spectrometric peak correlates with the mass of interest. The masses of these correlating peaks can also be associated with a set of compounds whose locations in the metabolic pathway must be bunched around the compound with the true annotation. 

It should be noted that the annotation algorithm has a restricted capacity for xenobiotic annotation. In general, xenobiotic biotransformation is accomplished by a limited number of enzymes with broad substrate specificities [11]. As a consequence, typical biotransformation methods (conjugation, oxidation, reduction, etc.) are common for the majority of xenobiotics and result in typical shifts in molecular weight for the correlating masses of different xenobiotics, yielding false context support (CS) and annotations. For this reason, xenobiotics were excluded from the annotation results.

Being metabolomics-related data, the obtained compound annotations should be related to Metabolomics Standards Initiative (MSI) chemical identification standards. Compound annotations are related to level 2 of metabolite identification (‘putatively annotated compounds’) according to the MSI standard, because two independent orthogonal features of each compound are used for annotation. Similar compounds identified together for the same mass relate to levels 2 and 3 (‘identification of class of compounds’). Identification of xenobiotics relates to level 3 and level 4 (‘unknown compound differentiated and quantified-based upon spectral data’) [12]. Thus, annotation results mainly include identifications at level 2 and a few portions at levels 3 and 4, and they do not include most robust identifications at level 1, which is acceptable for medical purposes and requires a chemical standard for identification. Obviously, for big data, to which the metabolomics data relates, a level 1 often is impossible, thus making it reasonable to use the described approach as a screening technology, which helps the clinician to optimize the selection of confirmatory, secondary tests [13].

The levels of compound identification, i.e., its putative annotations, determine the type of further data processing. The relatively low, albeit acceptable for metabolomics, identification probability suggests that the final data analysis should be based on the analysis of sets of metabolites. This allows us to ignore errors in the identification results associated with the level 2 identifications and to draw statistically valid conclusions. This approach is widespread in metabolomics, and MSEA seems to be the most well-known method. MSEA is a way to identify biologically meaningful patterns that are significantly enriched in metabolomic data. Essentially, MSEA is a metabolomic version of the popular gene set enrichment analysis [14], which is widely used in genomics data analysis, and investigates whether or not a set of metabolites is functionally related. It has the potential to identify subtle but consistent changes among a group of related compounds, which may go undetected with the individual evaluation of metabolites.

Applying MSEA to annotated compounds with an abnormal concentration in the blood revealed statistically significant alterations in steroidogenesis, androstenedione metabolism, and androgen and estrogen metabolism in obese patients. Most of the pronounced changes associated with the stage of obesity were detected in steroidogenesis. Projection of blood plasma metabolites with an abnormal concentration in obese patients on the steroidogenesis pathway showed that almost all steroidogenesis processes were altered. These data indicate that the proposed workflow works to reveal systemic changes on the molecular level in obese patients, suggesting further application of this approach to diagnosing obesity at the metabolome level, monitoring disease progression in a digitized manner, and estimating response after treatment. In addition, the proposed workflow is hypothesized to be acceptable for the investigation of other metabolic diseases.

Obesity is the result of many factors; therefore, it should be considered as a polygenic disease [15]. Obesity is characterized by many hormonal and metabolic features; however, to date, metabolic differences between obese and thin people cannot be unambiguously determined. This study is the first overview of this problem, and the data obtained from this study are consistent with data showing an association between being overweight or obese and steroid abnormalities. It has been demonstrated that steroid hormone dysfunction appears to be linked to the development of obesity [6,16,17] and that correction of steroid abnormalities may offer new approaches to therapy [18]. In addition, improvement of the relative androgen deficiency in men and ovarian hormone (estrogen) deficiency in women leads to improvement of the metabolic profile [7]. Nevertheless, substitutive hormonal treatments obviously need to be considered in the context of their effects or side effects on other systems. For example, female hormone replacement therapy has been seriously reconsidered or even abandoned by many women following data indicating that the oral combination of estrogens and a progestin causes a 26% increase in the incidence of breast cancer with a negative impact on cardiovascular events [19]. On the other hand, the link between androgen replacement and favorable body composition/fat distribution changes is increasingly recognized in hypogonadal, aging males [20]. Obviously, steroid-based therapies require monitoring of the metabolic profile. Therefore, the proposed workflow would be useful for such monitoring. This will certainly contribute to improving the quality of life and preventing the later and dangerous complications of obesity.

One of the most important results of this study is the difference in the alteration of steroidogenesis and other metabolic pathways, with some degree of overlap, in men and women. This result concerns the body mass index (BMI) as the criterion for the formation of the control and case groups. A recent population-based study from 200 countries has estimated that the global mean BMI is increasing; thus, it has become a serious public health concern [21]. A high BMI is a risk factor for a variety of diseases [22] and results in an increased morbidity and mortality [23]. Some studies have examined the association between BMI and health-related quality of life (HRQOL) [24,25], which has been recognized as a valid health indicator [26,27]. However, the impact of BMI on HRQOL may vary by gender. One study has shown that at higher BMI values, men report a higher HRQOL than women [28]. Meanwhile, another study has reported that the association between obesity and a lower HRQOL was significant for women, but not for men [21]. Furthermore, it has been demonstrated that obesity in women was negatively and significantly associated with HRQOL, whereas in men this association was positive but not statistically significant [29]. The overall view on the metabolic pathways provided by our approach may give a possible explanation for these findings. The association between BMI and HRQOL differs by gender. Therefore, there was evidence of the so-called “obesity-HRQOL paradox” [29], with more pronounced alterations in metabolic pathways in women, whereas men at the same BMI values have alterations in the same pathways and in the same direction (downregulated), but they are sufficiently less pronounced. Our results suggest that from the viewpoint of biochemical insights, the BMI borders should be corrected to make them more consistent with the HRQOL, which will make the BMI more valid for implementing targeted weight control programs and appropriate interventions to improve the HRQOL.

## 4. Materials and Methods 

### 4.1. Blood Samples

Venous blood was collected from 100 volunteers (20 healthy subjects, 20 overweight subjects, and 60 subjects with stage 1, 2, or 3 obesity, according to the World Health Organization classification of obesity by BMI) into EDTA Vacutainer plasma tubes (BD, Franklin Lakes, NJ, USA) at the Federal State Budgetary Institution “Nutrition and Biotechnology” (Moscow, Russia). Table 3 summarizes the cohort characteristics. To reduce the effect of food intake on the metabolic composition of the blood, blood was collected before breakfast. The subjects were not treated with medication at the time of the blood sampling. An anamnesis was collected, the overall condition of the body was evaluated by a doctor, a laboratory study of blood (14 parameters) and urine (11 parameters) was carried out, resting energy expenditures were determined, and the body composition was estimated by bioimpedance measurements. Based on the results obtained, the doctor categorized the volunteers into the appropriate group. The groups of cases included volunteers with obesity of varying stages with a diagnosis of E 66.0, according to the International Classification of Diseases (obesity of exchange-alimentary origin). The presence of hyperuremia, dyslipidemia, and steatosis (in stage 3 obesity) in the case groups was allowed.

Blood samples were processed according to the manufacturer’s instructions [30]. The resultant blood plasma was stored at −80 °C until analysis (no more than two months). The analyzed samples were subjected to one freeze/thaw cycle. Plasma (10 μL) was mixed with 10 μL of water (LiChrosolv; Merck KGaA, Darmstadt, Germany) and 80 μL of methanol (Fluka, Munich, Germany). After incubation at room temperature for 10 min, the samples were centrifuged at 13,000× *g* (Centrifuge 5804R; Eppendorf AG, Hamburg, Germany) for 15 min. The supernatant was then transferred to clean plastic Eppendorf™ tubes, and fifty volumes of methanol containing 0.1% formic acid (Fluka, Munich, Germany) were added to each tube. The resulting solutions were subjected to mass spectrometry analysis. All procedures performed in studies involving human participants were in accordance with the ethical standards of the institutional or national research committee and with the 1964 Helsinki declaration and its later amendments or comparable ethical standards. The study was approved by the relevant ethical review committee #28-01 (Moscow, Russia; approval number #102). Informed consent was obtained from all individual participants included in the study. 

### 4.2. Mass Spectrometry

Samples were analyzed by a hybrid quadrupole time-of-flight mass spectrometer (maXis Impact, Bruker Daltonics, Billerica, MA, USA) equipped with an electrospray ionization (ESI) source. The mass spectrometer was set up to prioritize the detection of ions with a mass-to-charge ratio (*m*/*z*) ranging from 45 to 900 Da, with a mass accuracy of 1–3 parts per million (ppm). The spectra were recorded in the positive ion charge detection mode. The samples were injected into the ESI source using a glass syringe (Hamilton Bonaduz AG, Bonaduz, Switzerland) connected to a syringe injection pump (KD Scientific, Holliston, MA, USA). The rate of sample flow to the ionization source was 180 µL/h. Technical replicates were not performed. Mass spectra were obtained using DataAnalysis version 3.4 (Bruker Daltonics, Billerica, MA, USA) to summarize one-minute signals.

### 4.3. Mass Spectra Preprocessing

Recalibration, peak detection, and peak intensity calculation of mass spectra were carried out automatically by DataAnalysis software. Masses of compounds were determined from the mass spectrum peaks obtained using the following parameters: peak width, 2; signal-to-noise ratio, 1; relative and absolute threshold intensity, 0.01% and 100, respectively. For recalibration of all the peak intensity values, the internal standard losartan (*m*/*z* 423.169) was used. Normalization of mass peak intensities was performed by dividing the intensity by the normalization value, which was calculated for each peak separately as follows: the 50 Da range (started 25 Da before and ended 25 Da after the *m*/*z* of the mass peak) was selected; all peaks inside the range were sorted in descending order according to their intensities; the intensity of the 150th peak was selected as the normalization value. Such intensities improved the annotation algorithm due to the correction of ion suppression of peak intensities that resulted in a more correct calculation of the correlation coefficients (see the calculation of the correlation matrix below).

Alignment of the *m*/*z* values of the mass peaks to the different mass spectra was performed as described previously [31]. The resulting *m*/*z* values with a nonzero mass peak intensity for more than nine samples were submitted to the metabolite search engine, MassTRIX (Helmholtz Centre, Munich, Germany; http://masstrix3.helmholtz-muenchen.de), with the following parameters: scan mode, positive ionization (corrected for H^+^, Na^+^, K^+^ adducts); max. error, 0.005 Da; database, KEGG/HMDB/LipidMaps with isotopes; organism, *Homo sapiens*. The retrieved list of metabolite names for each submitted *m*/*z* value was further processed by the compound annotation algorithm.

The workflow used in this study to analyze blood plasma samples from obese patients is presented in Figure 1. 

### 4.4. Compound Annotation Algorithm

**Figure 4:**
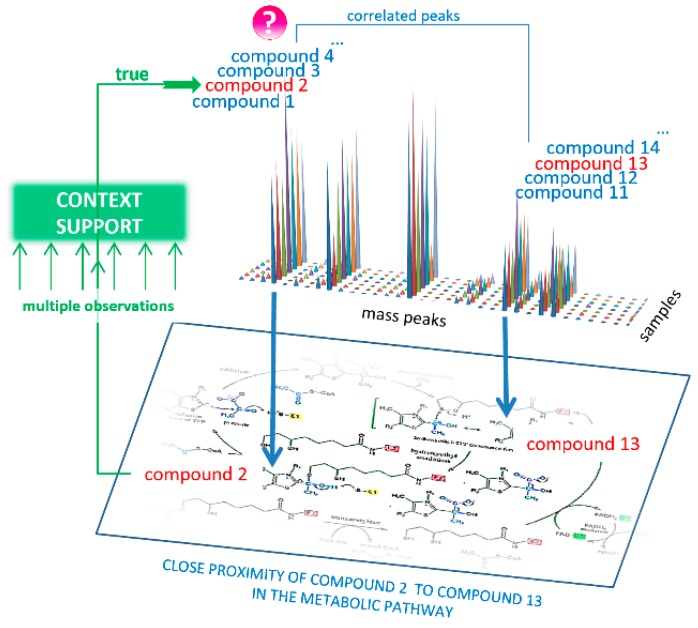
Concept of the annotation of compounds from mass spectra using biological context data. The mass peak (marked by ‘?’), the intensity of which reflects a concentration of the compound in the sample, is associated with a set of possible compound names (labeled as compounds 1–4), whose molecular weights matched this peak. The essence of the annotation algorithm lies in the fact that the concentrations of compounds in the samples from living organisms are interrelated by metabolic pathways. Therefore, the correct compound name (labeled as compound 2) should be supported by a list of possible compound names (labeled as compounds 11–14) that match the correlated mass peaks because among these names, the names of the compound closely located in the pathway should be presented (labeled as compound 13). If numerous correlated mass peaks are used, the locations of the associated compounds in the metabolic pathways provide statistically sound support (labeled as ‘context support’) to annotate the correct compound name (compound 2).

The purpose of the compound annotation algorithm is to select only true annotations from the retrieved list of metabolite names using CS from biological data—supporting the annotation result by mutual confirmation of the mass spectrometry data and data on metabolic pathways (Figure 4). 

The following steps were taken:

- Coefficients for Pearson correlation between aligned mass peak intensities were calculated using the *corr* function in MATLAB (MathWorks, Natick, MA, USA). As a result, the correlation matrix was built (columns correspond to *m*/*z* values of peaks for which correlation was calculated, rows and columns correspond to other *m*/*z* values of peaks taken to calculate pairwise correlation, in matrix cells correlation coefficients (***r***) are presented). Correlation values were calculated only if at least nine nonzero values were available to calculate correlation. Zero values in the matrix cells were not used in the calculation of the correlation. To find correlations, the mass spectra of all samples involved in the study were used.
- Each mass spectrometry peak was tested for support from context data (Figure 4). Using the correlation matrix, the top 30 (empirically established as optimal) highly correlated (positive or negative, with ***r*** < 0.85) mass peaks were also associated with KEGG IDs proposed by MassTRIX, which were considered CS for the tested peak. Highly correlated peaks from CS with ***r*** > 0.85 were annotated as the derivatives of the tested peaks (fragment, adduct, or multi-ion of the compound).
- To reveal the true compound name, the distance between each candidate name of the compound and the compound name in its CS was calculated. To do this, the distance matrix between compounds in the human metabolic pathways was generated using Metabonetworks toolbox [32]. A distance equal to N means that the distance from the testing annotation to a particular annotation from the CS lies through the N-1 intermediate compounds (i.e., shortest way through the N biochemical reactions). The retrieved values for the N = 1, 2, 3, and 4 positions were tested against random cause. CS was randomly generated 300 times, and the retrieved data for the N = 1, 2, 3, and 4 positions were used to calculate the *p*-value for candidates using the cumulative function for generalized extreme value distribution (1-*cdf* function with *gev* option in MATLAB).
- A probability of *p* > 0.95 for any N 1–4 position statistically confirms that the tested annotation is verified by CS, i.e., it is a true annotation.
- Retrieved true annotations were submitted to the compound annotation algorithm again, and this process was repeated six times. Each time, the CS became more competent. Finally, annotations with a Z score<-1.64 (correspond to probability of *p* > 0.95) were considered the output of the annotation algorithm.

**Figure 5:**
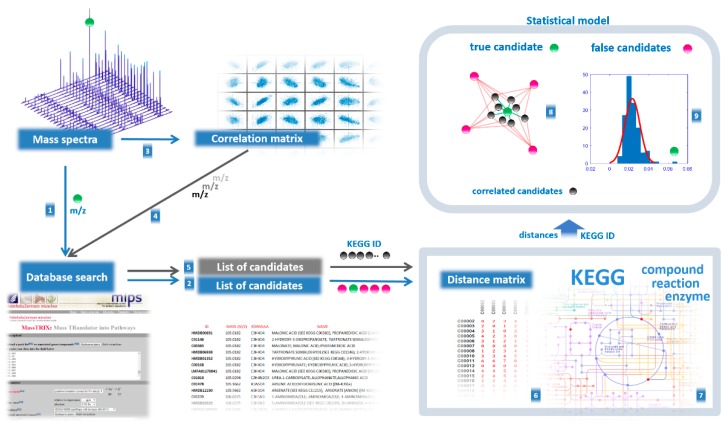
Schematic for compound annotation used in this study. The mass peak *m*/*z* value for the annotating compound (●) is submitted to a metabolite search engine (**1**), and a list of candidates of compound names is retrieved (**2**). From the set of mass spectra, the correlation between intensity of the mass peak of the annotating compound and other peaks is calculated (**3**), and the most highly correlated are also submitted to the search engine (**4**) to retrieve a list of candidates (**5**). Each candidate in the list for the annotating compound is sequentially applied to a distance matrix (**6**) with candidates for the correlating masses from another list in order to measure the distance between them. The distance matrix includes distances between compounds in biochemical pathways and was calculated using the *KEGG compound*, *KEGG reaction*, and *KEGG enzyme* databases (**7**). The retrieved distances are used to find the true name for the annotating compound, which is surrounded by candidates with correlating peaks (**8**) that are confirmed by *p*-values from the statistical model based on distances.

Finally, xenobiotics were considered to be false annotations and were excluded from the annotation results. The schematic for compound annotation used in this study is presented in Figure 5. To perform all calculations, a Sony-VAIO (Sony Corporation, Tokyo, Japan; Intel^®^ Core™ i7-2640 CPU 2.80 GHz; Windows 10 Pro) personal computer was used.

### 4.5. Statistical Analysis

To reveal mass spectrometry peaks associated with being overweight or different stages of obesity, the aligned and normalized intensities of the mass spectrometry peaks were analyzed by the Wilcoxon rank sum test (*ranksum* function in Matlab), and mass peaks with *p* < 0.01 were considered to be associated with the corresponding stage of obesity. Statistical analysis was applied to the following groups: females with a normal weight vs. overweight females and females at different stages of obesity; males with a normal weight vs. overweight males and males at different stages of obesity; and all subjects with a normal weight vs. all overweight subjects and subjects at different stages of obesity (Figures S1–S12, Tables S3–S14). 

### 4.6. Metabolite Set Enrichment Analysis

MSEA [33] was applied using MetaboAnalyst 4.0 software (https://www.metaboanalyst.ca/ MetaboAnalyst/home.xhtml) [34] with the following options: module, enrichment analysis; compound names, KEGG ID; metabolite set library, pathway-associated metabolite sets (SMPDB; 99 metabolite sets based on normal human metabolic pathways); type of enrichment analysis, over-representation analysis (ORA). This type is performed when a list of compound names is provided. ORA is implemented using the *hypergeometric test* to evaluate whether a particular metabolite set is represented more than expected by chance within the given compound list. One-tailed *p*-values were provided by ORA after adjusting for multiple testing.

To project the blood plasma metabolites with an abnormal concentration in patients on the steroidogenesis pathway, MetaboAnalyst was used with the following options: module, pathway analysis; compound names, KEGG ID; pathway analysis algorithm, ORA (hypergeometric test); pathway topology analysis, relative-betweenness centrality; pathway library, *Homo sapiens* (KEGG).

## 5. Conclusions

This metabolomics study of blood samples using enhanced compound annotation based on the biological context provided an overview of alterations at different stages of obesity at the molecular level. The results suggested a metabolome-based explanation of the “obesity-HRQOL paradox.” Application of the proposed workflow affords digitized data sets, which may be useful for diagnostics, as well as monitoring of treatment response. This workflow is also expected to be suitable for studying other metabolic diseases. 

## Figures and Tables

**Table 1:** Variables associated with this study.

Parameter	Value
Detection mass range of compounds (*m*/*z*)	45–900
Number of detected compound mass peaks	9333 ± 416 ^1^
Number of masses submitted to MassTrix	14,738
Number of mass peaks/compound candidate submitted to the annotation algorithm	26,430
Number of mass peaks with putatively annotated compound(s) by the annotation algorithm	2015
Number of unique compound names retrieved by the annotation algorithm	386

^1^ average ± standard deviation.

**Table 2:** Results of metabolite set enrichment analysis.

Metabolite Set	*p* Value ^1^											
	♂♀ vs. ♂♀				♂ vs. ♂				♀ vs. ♀			
	Normal vs. Overweight	Normal vs. Stage 1 Obesity	Normal vs. Stage 2 Obesity	Normal vs. Stage 3 Obesity	Normal vs. Overweight	Normal vs. Stage 1 Obesity	Normal vs. Stage 2 Obesity	Normal vs. Stage 3 Obesity	Normal vs. Overweight	Normal vs. Stage 1 Obesity	Normal vs. Stage 2 Obesity	Normal vs. Stage 3 Obesity
Steroidogenesis	3 × 10^−6^	2 × 10^−5^	5 × 10^−8^	5 × 10^−12^	0.477	-	0.004	0.002	0.094	6 × 10^−7^	1 × 10^−6^	1 × 10^−10^
Androstenedione metabolism	4 × 10^−4^	0.013	0.017	0.014	-	0.173	0.113	0.045	0.258	0.011	0.065	6 × 10^−4^
Androgen and estrogen metabolism	9 × 10^−4^	0.011	0.011	0.002	-	-	0.076	0.023	0.042	0.002	0.004	2 × 10^−5^
Aspartate metabolism	0.062	0.047	0.047	0.378	0.409	0.244	0.251	0.338	0.426	0.015	0.191	0.198
Urea cycle	0.085	0.041	0.120	0.669	0.352	0.019	0.429	0.261	0.027	0.035	0.294	0.302
Ammonia recycling	0.119	0.154	0.171	0.514	0.381	0.225	0.21	0.3	0.744	0.061	0.633	0.358
Estrone metabolism	0.131	0.449	0.56	0.536	-	-	0.711	0.57	0.258	0.426	0.204	0.210
Beta-alanine metabolism	0.144	0.733	0.381	0.353	-	0.237	0.829	0.699	0.765	0.322	0.387	0.674
Malate-aspartate shuttle	0.199	0.158	0.106	0.323	0.138	0.075	0.401	0.295	0.343	0.148	0.135	0.139
Glucose-alanine cycle	0.294	0.276	0.471	0.798	-	0.097	0.487	0.365	0.422	0.553	0.587	0.593
Glycine and serine metabolism	0.364	0.005	0.038	0.130	-	0.379	0.338	0.137	0.219	0.010	0.55	0.191
Cysteine metabolism	0.368	0.515	0.376	0.351	-	0.187	0.375	0.600	-	0.491	0.517	0.836
Retinol metabolism	0.369	0.794	0.662	0.426	-	-	0.279	0.73	0.794	0.774	0.219	0.227
Tryptophan metabolism	0.38	0.772	0.662	0.55	0.598	0.384	0.586	0.337	0.228	0.744	0.985	0.778
Glutamate metabolism	0.38	0.034	0.662	0.505	0.523	0.325	0.715	0.826	0.609	0.0617	0.636	0.646
Phenylalanine and tyrosine metabolism	0.415	0.783	0.433	0.645	-	0.0182	0.411	0.248	0.0245	0.177	0.275	0.283
Alanine metabolism	0.419	0.211	0.33	0.877	-	0.384	0.583	0.449	0.512	0.422	0.686	0.692
Lysine degradation	0.461	0.822	0.715	0.692	-	0.212	0.447	0.653	-	0.611	0.873	0.605
Histidine metabolism	0.486	0.585	0.407	0.692	-	0.291	0.363	0.438	0.841	0.379	0.544	0.795
Citric acid cycle	0.505	0.483	0.542	0.734	-	-	0.81	0.677	0.744	0.271	0.350	0.641

^1^ Red font indicates *p* < 0.01; while green font indicates 0.01 < *p* < 0.05.

**Table 3:** Study cohort characteristics.

Group	Body Height (cm)	Body Weight (kg)	Age (Years)	Body Mass Index	Gender (Male/Female)
Normal	173.5 ± 8.2 ^1^	66.9 ± 9.4	31.3 ± 5.5	22.1 ± 1.9	10/10
Overweight	172.1 ± 12.2	82.0 ± 13.1	32.9 ± 6.7	27.5 ± 1.3	10/10
Stage 1 obesity	170.6 ± 11.7	95.1 ± 13.7	29.7 ± 8.0	32.5 ± 11.7	10/10
Stage 2 obesity	171.5 ± 9.4	109.1 ± 13.8	32.8 ± 8.1	36.9 ± 1.3	10/10
Stage 3 obesity	172.3 ± 9.9	141.0 ± 27.4	34.5 ± 6.5	47.3 ± 6.1	10/10

^1^ mean ± standard deviation.

## Supplementary Materials

The following are available online at https://www.mdpi.com/1422-0067/21/2/568/s1.
