# Supplementary material for: Mass Spectrometry-Based Metabolomics Analysis of Obese Patients’ Blood Plasma
Source: Int J Mol Sci. 2020 Jan 15;21(2):568. doi: 10.3390/ijms21020568 (PMC7014187; doi:10.3390/ijms21020568)
Supplement: Supplementary file 1 [file ijms-21-00568-s001.pdf]

Article

Mass spectrometry-based metabolomics analysis of obese patients' blood plasma

(supplementary materials)

Table S1. Study cohort characteristics.

Group	Body height (cm)	Body weight (kg)	Age (years)	BMI	Gender
Normal	162.0	64.0	34.0	24.4	female
	183.0	68.0	39.0	19.0	female
	168.0	62.0	32.0	22.0	female
	164.0	49.0	28.0	18.2	female
	162.0	58.0	32.0	22.1	female
	159.0	53.9	31.0	21.3	female
	176.0	65.0	38.0	21.0	female
	167.0	59.2	23.0	21.2	female
	173.0	68.2	31.0	22.8	female
	170.5	63.3	28.0	21.8	female
	186.0	89.0	36.0	25.7	male
	178.0	69.0	32.0	21.8	male
	179.0	71.0	23.0	22.2	male
	184.0	71.0	25.0	21.0	male
	167.6	67.0	35.0	23.9	male
	174.6	66.0	25.0	21.6	male
	177.8	68.5	43.0	21.7	male
	179.4	79.9	27.0	24.8	male
	184.0	83.4	28.0	24.6	male
	175.1	62.0	36.0	20.2	male
mean	*173.5*	*66.9*	*31.3*	*22.1*	
st. dev.	*8.2*	*9.4*	*5.5*	*1.9*	
Overweight	158.0	72.0	30.0	28.8	female
	165.0	81.0	28.0	29.8	female
	161.8	69.3	32.0	26.5	female
	168.0	74.4	22.0	26.2	female
	176.4	82.0	44.0	26.3	female
	150.0	57.8	26.0	25.7	female
	175.4	84.4	27.0	27.4	female
	162.0	70.3	42.0	26.8	female
	164.0	74.0	44.0	27.5	female
	156.8	64.3	32.0	26.1	female
	172.0	87.4	38.0	29.5	male
	188.0	91.0	40.0	25.7	male
	190.0	105.0	29.0	29.0	male
	173.0	80.0	37.0	26.7	male
	178.6	86.8	42.0	27.2	male
	184.4	90.5	31.0	26.6	male
	182.8	91.4	31.0	27.3	male
	175.0	87.0	28.0	28.4	male
	164.7	77.8	28.0	28.7	male
	196.0	113.3	27.0	29.5	male
mean	*172.1*	*82.0*	*32.9*	*27.5*	

st. dev.	*12.2*	*13.1*	*6.7*	*1.3*	
	165.0	95.0	36.0	34.9	female
	145.0	73.0	40.0	34.7	female
	161.0	82.6	45.0	31.9	female
	162.8	91.2	34.0	34.4	female
	154.0	72.1	42.0	30.4	female
	168.7	96.7	20.0	34.0	female
	159.5	76.8	41.0	30.2	female
	174.0	100.0	27.0	33.0	female
	156.0	82.2	20.0	33.8	female
Stage 1 obesity	171.9	90.1	25.0	30.5	female
	178.2	110.1	29.0	34.7	male
	187.0	119.6	29.0	34.3	male
	179.0	96.5	26.0	30.1	male
	183.2	108.0	18.0	32.2	male
	182.0	110.2	30.0	33.3	male
	172.0	92.0	31.0	31.1	male
	180.0	106.0	32.0	32.7	male
	185.1	109.5	19.0	32.0	male
	165.0	83.4	25.0	30.6	male
	182.1	106.4	25.0	32.1	male
mean	*170.6*	*95.1*	*29.7*	*32.5*	
st. dev.	*11.7*	*13.7*	*8.0*	*1.7*	
	168.0	112.3	26.0	39.8	female
	163.0	96.5	38.0	36.3	female
	166.0	101.1	34.0	36.7	female
	160.0	91.9	18.0	35.9	female
	168.0	106.4	36.0	37.7	female
	168.7	100.0	23.0	35.1	female
	171.0	112.0	45.0	38.3	female
	167.0	104.0	33.0	37.3	female
	164.6	101.7	41.0	37.5	female
Stage 2 obesity	171.8	105.5	33.0	35.8	female
	179.5	122.2	23.0	37.9	male
	187.0	133.8	25.0	38.3	male
	181.2	116.9	29.0	35.6	male
	165.0	98.2	42.0	36.1	male
	165.0	99.3	39.0	36.5	male
	186.8	126.1	43.0	36.1	male
	167.8	100.9	21.0	35.8	male
	158.0	88.9	32.0	35.6	male
	187.0	131.0	33.0	37.5	male
	185.0	133.8	42.0	39.1	male
mean	*171.5*	*109.1*	*32.8*	*36.9*	
st. dev.	*9.4*	*13.8*	*8.1*	*1.3*	
	164.0	113.0	41.0	42.0	female
	162.7	118.5	38.0	44.8	female
	155.0	104.0	39.0	43.3	female
	171.8	120.8	39.0	40.9	female
	152.7	97.5	32.0	41.8	female
	169.1	116.8	36.0	40.9	female
Stage 3 obesity	165.2	114.0	37.0	41.8	female
	167.7	140.9	45.0	50.1	female
	172.2	133.1	31.0	44.9	female
	175.3	175.7	36.0	57.5	female
	176.2	133.9	22.0	43.1	male
	177.0	193.0	36.0	64.2	male
	179.0	151.0	34.0	48.4	male
	184.0	183.0	34.0	54.1	male

	167.0	137.0	28.0	49.1	male
	173.8	159.1	40.0	52.7	male
	186.1	153.2	29.0	44.2	male
	192.6	172.5	41.0	46.5	male
	173.0	137.7	33.0	46.1	male
	181.6	165.6	18.0	50.2	male
mean	*172.3*	*141.0*	*34.5*	*47.3*	
st. dev.	*9.9*	*27.4*	*6.5*	*6.1*	

Table S2. Statistical data for mass peak intensities for altered metabolites in steroidogenesis pathway (data for Normal and obesity stage 3 are presented).

KEGG ID	m/z	Normal		Stage 3 obesity	
		mean	st. dev.	mean	st. dev.
C00280*	287.196	1.69	0.94	0.71	0.52
C00410	309.217	17.65	5.65	8.64	1.72
C00410	345.236	6.91	0.86	3.76	1.48
C00410	101.119	3.31	0.56	1.85	0.42
C00410	339.192	11.30	2.45	6.28	1.21
C00410	322.185	1.15	0.37	0.48	0.20
C00410	310.222	4.22	1.42	2.02	0.25
C00410	324.201	2.09	0.50	1.16	0.45
C00410	140.104	1.01	0.33	0.46	0.16
C00410	321.181	4.55	1.53	2.12	0.30
C00410	327.228	88.11	23.11	50.17	10.84
C00410	329.243	19.85	6.83	9.74	2.21
C00410	341.207	25.28	6.19	13.25	1.65
C00410	355.188	4.86	0.49	2.96	0.68
C00410	315.229	5.16	1.75	2.74	0.59
C00410	323.197	8.49	2.18	4.59	0.80
C00410	330.246	4.77	1.91	2.01	0.39
C00410	326.216	7.07	2.94	3.48	0.99
C00410	328.232	19.58	5.42	10.97	2.22
C00410	342.209	6.05	1.96	2.95	0.31
C00410	325.212	30.88	12.43	16.07	5.21
C00410	100.112	2.47	1.29	0.90	0.46
C00410	122.096	9.95	3.83	5.50	1.62
C00410	235.238	0.36	0.18	0.24	0.07
C00410	86.096	14.27	8.78	4.63	2.06
C00410	123.099	0.96	0.44	0.48	0.16
C00468	283.222	0.29	0.82	0.74	0.94
C00523	313.209	11.24	4.86	3.77	1.10
C00523	359.215	13.45	5.57	5.15	1.20
C00523	273.22	0.79	0.35	0.27	0.33
C00523	387.249	4.39	2.73	0.91	0.56
C00523	364.32	0.63	0.16	0.26	0.36
C00523	357.202	11.58	5.03	6.60	7.89
C00523	291.23	1.27	0.80	0.43	0.53
C00535	361.27	2.85	0.86	1.08	0.79
C00535	285.22	2.37	0.51	1.25	0.39
C00535	288.239	1.83	0.48	1.00	0.24
C00535	135.08	0.94	0.39	0.42	0.10
C00535	345.208	6.90	3.42	2.31	0.76
C00535	287.236	6.32	1.27	3.80	0.72
C00535	233.151	1.48	0.83	0.52	0.22
C00535	289.214	1.53	0.68	0.64	0.26
C00535	289.251	2.05	0.66	1.14	0.34
C00535	290.255	0.56	0.28	0.31	0.12

C00535	275.236	0.48	0.25	0.20	0.13
C00535	286.222	0.67	0.15	0.47	0.17
C00581	118.064	0.71	0.53	0.13	0.11
C00674	289.214	1.53	0.68	0.64	0.26
C00674	311.195	4.01	2.32	1.38	0.68
C00674	311.195	4.01	2.32	1.38	0.68
C00735	290.213	0.58	0.33	0.17	0.09
C00735	330.206	2.06	1.15	0.69	0.33
C00735	211.128	0.78	0.48	0.26	0.11
C00735	391.221	3.21	2.20	0.72	0.71
C00762	361.205	4.22	2.42	1.09	0.95
C00762	479.33	15.17	9.75	4.21	1.89
C00951	281.151	1.55	0.38	0.57	0.44
C00951	307.201	1.99	0.00	1.30	0.00
C00951	311.233	4.86	1.66	2.30	1.33
C00951	295.165	1.71	0.81	0.63	0.32
C00956	112.051	0.35	0.49	0.06	0.15
C01176	353.208	4.34	2.63	1.13	0.61
C01227	289.214	1.53	0.68	0.64	0.26
C01227	311.195	4.01	2.32	1.38	0.68
C01780	361.205	4.22	2.42	1.09	0.95
C01953	895.302	0.17	0.39	0.18	0.15
C02140	313.141	0.85	0.30	0.21	0.27
C02140	283.171	1.92	0.96	0.66	0.40
C02140	361.233	5.25	2.63	1.90	0.63
C02140	333.239	2.27	1.25	0.69	0.44
C02140	347.217	2.86	1.64	0.93	0.75
C03205	353.208	4.34	2.63	1.13	0.61
C03772	289.214	1.53	0.68	0.64	0.26
C03772	311.195	4.01	2.32	1.38	0.68
C03917	313.209	11.24	4.86	3.77	1.10
C03917	291.23	1.27	0.80	0.43	0.53
C04295	313.209	11.24	4.86	3.77	1.10
C04295	291.23	1.27	0.80	0.43	0.53
C04373	313.209	11.24	4.86	3.77	1.10
C04373	291.23	1.27	0.80	0.43	0.53
C04392	666.259	0.71	1.39	0.62	0.27
C05138	451.353	1.99	1.13	0.65	0.45
C05138	491.293	4.42	2.93	0.98	0.66
C05138	348.218	1.15	0.68	0.35	0.35
C05139	305.209	2.27	1.38	0.83	0.41
C05141	335.162	1.99	0.76	0.60	0.57
C05141	336.169	0.72	0.37	0.16	0.22
C05141	284.206	0.51	0.12	0.25	0.16
C05141	111.064	0.30	0.03	0.18	0.09
C05141	437.334	1.78	0.53	0.81	0.69
C05141	283.204	1.64	0.45	0.74	0.67
C05141	297.219	1.17	0.14	0.70	0.44
C05141	280.19	0.53	0.26	0.13	0.20
C05141	313.176	2.09	1.17	0.63	0.60
C05141	293.176	2.07	1.62	0.52	0.63
C05291	305.209	2.27	1.38	0.83	0.41
C05293	313.209	11.24	4.86	3.77	1.10
C05293	291.23	1.27	0.80	0.43	0.53
C05294	305.209	2.27	1.38	0.83	0.41
C05472	368.249	1.73	1.07	0.46	0.23
C05476	183.113	0.27	0.00	0.20	0.00
C05477	347.217	2.86	1.64	0.93	0.75
C05480	341.241	8.58	3.47	2.87	1.53
C05480	342.246	2.20	0.79	1.06	0.71
C05480	360.219	3.78	2.02	1.77	0.34
C05485	333.239	2.27	1.25	0.69	0.44

C05488	347.217	2.86	1.64	0.93	0.75
C05490	345.208	6.90	3.42	2.31	0.76
C05490	355.218	7.92	3.77	2.79	0.62
C05497	347.217	2.86	1.64	0.93	0.75
C05498	353.208	4.34	2.63	1.13	0.61
C13712	341.241	8.58	3.47	2.87	1.53
C13712	342.246	2.20	0.79	1.06	0.71

* Metabolite names

C00280	Androstenedione
C00410	Progesterone
C00468	Estrone
C00523	Androsterone
C00535	Testosterone
C00581	Guanidinoacetate
C00674	Androstanedione
C00735	Cortisol
C00762	Cortisone
C00951	Estradiol
C00956	L-2-Aminoadipate
C01176	17alpha-Hydroxyprogesterone
C01227	Dehydroepiandrosterone
C01780	Aldosterone
C01953	Pregnenolone
C02140	Corticosterone
C03205	11-Deoxycorticosterone
C03772	5beta-Androstane-3,17-dione
C03917	Dihydrotestosterone
C04295	Androstenediol
C04373	Etiocholanolone
C04392	P1,P4-Bis(5'-xanthosyl) tetraphosphate
C05138	17alpha-Hydroxypregnenolone
C05139	16alpha-Hydroxydehydroepiandrosterone
C05141	Estriol
C05291	7alpha-Hydroxytestosterone
C05293	5beta-Dihydrotestosterone
C05294	19-Hydroxytestosterone
C05472	Urocortisol
C05476	Tetrahydrocorticosterone
C05477	21-Hydroxy-5beta-pregnane-3,11,20-trione
C05480	Pregnanolone
C05485	21-Hydroxypregnenolone
C05488	11-Deoxycortisol; Cortodoxone
C05490	11-Dehydrocorticosterone
C05497	21-Deoxycortisol
C05498	11beta-Hydroxyprogesterone
C13712	Allopregnanolone

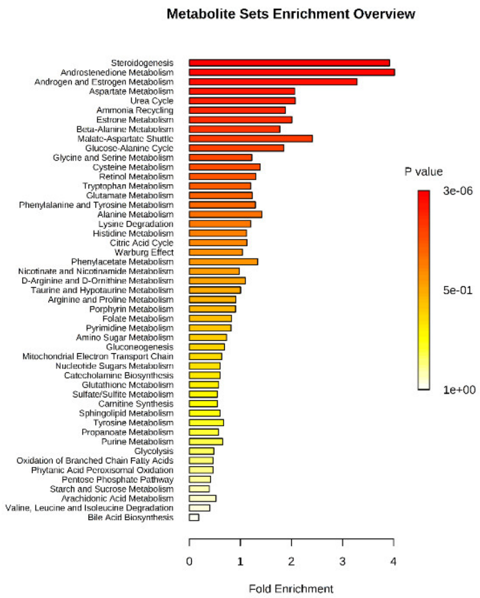

Figure S1. Summary plot for over-representation analysis (ORA) of blood plasma metabolites with an abnormal concentration in overweight males and females. Other ORA related data is presented in Table S3. Supportive information Figures S1-S12 were generated by MetaboAnalyst.

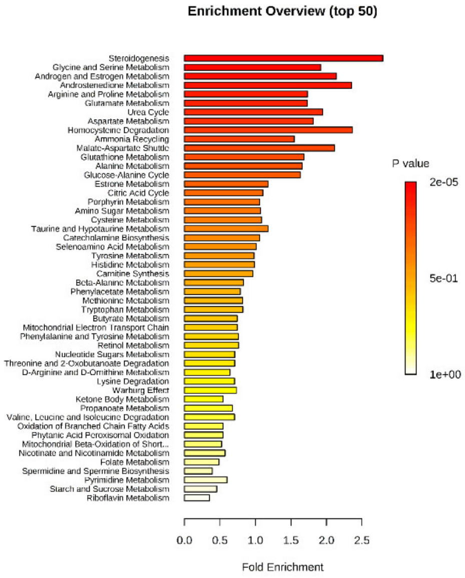

Figure S2. Summary plot for over-representation analysis (ORA) of blood plasma metabolites with an abnormal concentration in obese patients (stage 1 obesity in males and females). Other ORA related data is presented in Table S4.

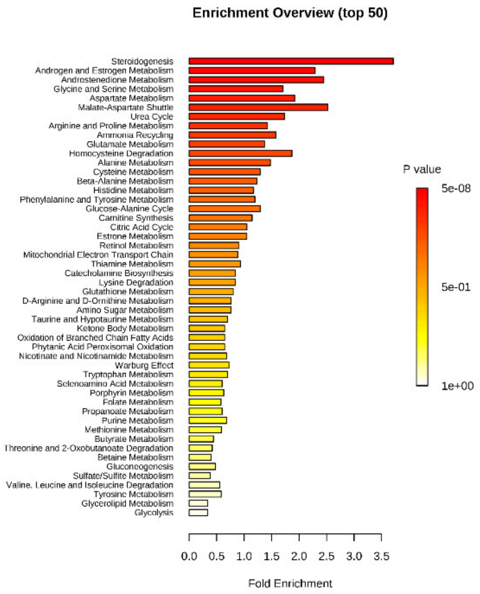

Figure S3. Summary plot for over-representation analysis (ORA) of blood plasma metabolites with an abnormal concentration in obese patients (stage 2 obesity in males and females). Other ORA related data is presented in Table S5.

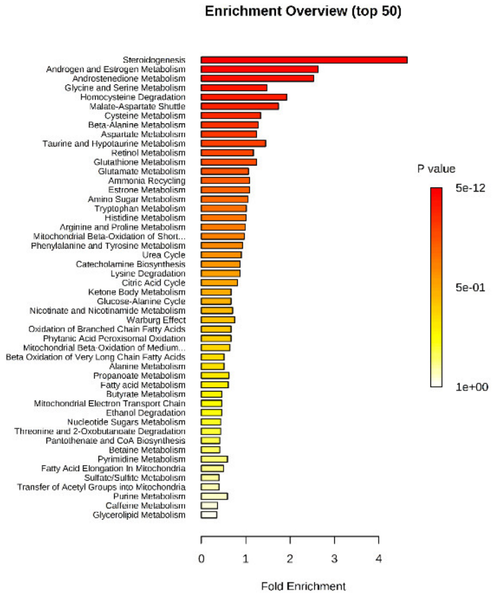

Figure S4. Summary plot for over-representation analysis (ORA) of blood plasma metabolites with an abnormal concentration in obese patients (stage 3 obesity in males and females). Other ORA related data is presented in Table S6.

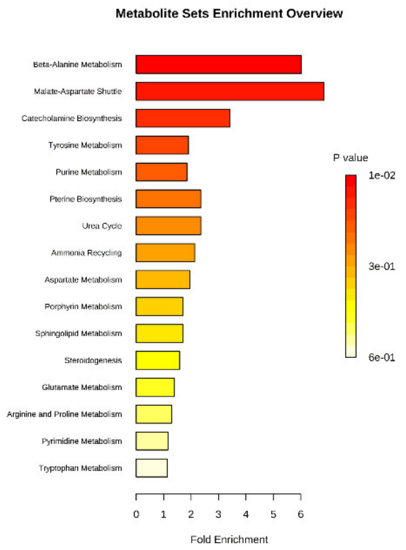

Figure S5. Summary plot for over-representation analysis (ORA) of blood plasma metabolites with an abnormal concentration in overweight males. Other ORA related data is presented in Table S7.

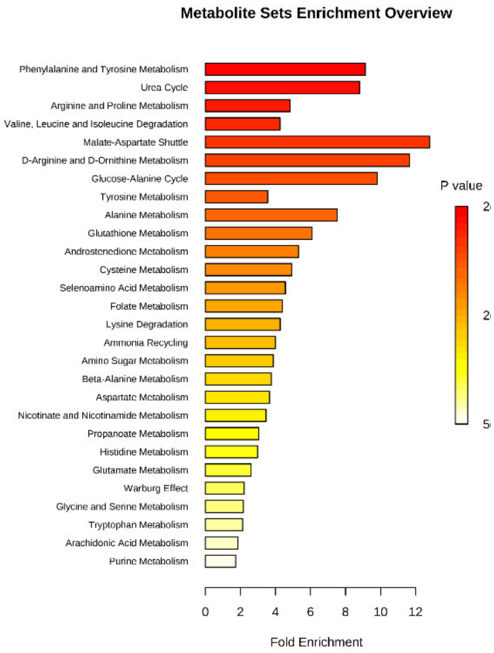

Figure S6. Summary plot for over-representation analysis (ORA) of blood plasma metabolites with an abnormal concentration in obese patients (stage 1 obesity in males). Other ORA related data is presented in Table S8.

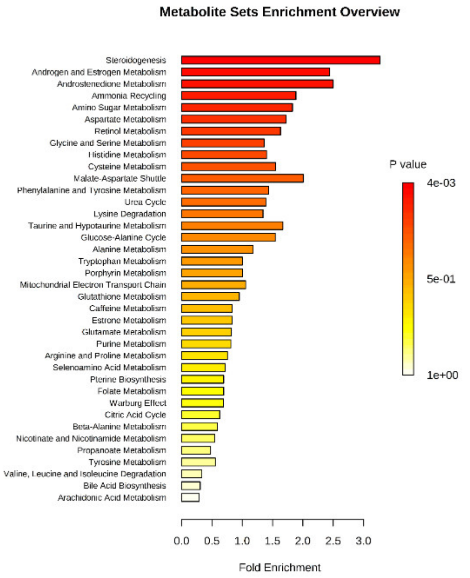

Figure S7. Summary plot for over-representation analysis (ORA) of blood plasma metabolites with an abnormal concentration in obese patients (stage 2 obesity in males). Other ORA related data is presented in Table S9.

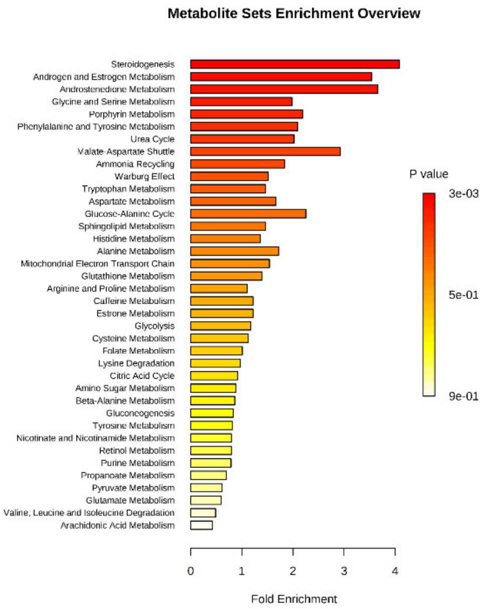

Figure S8. Summary plot for over-representation analysis (ORA) of blood plasma metabolites with an abnormal concentration in obese patients (stage 3 obesity in males). Other ORA related data is presented in Table S10.

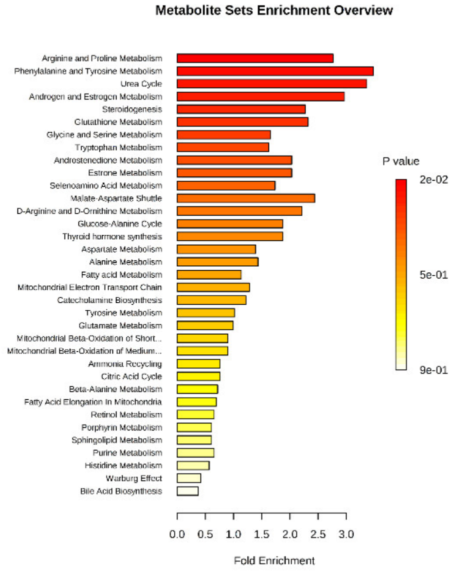

Figure S9. Summary plot for over-representation analysis (ORA) of blood plasma metabolites with an abnormal concentration in overweight females. Other ORA related data is presented in Table S11.

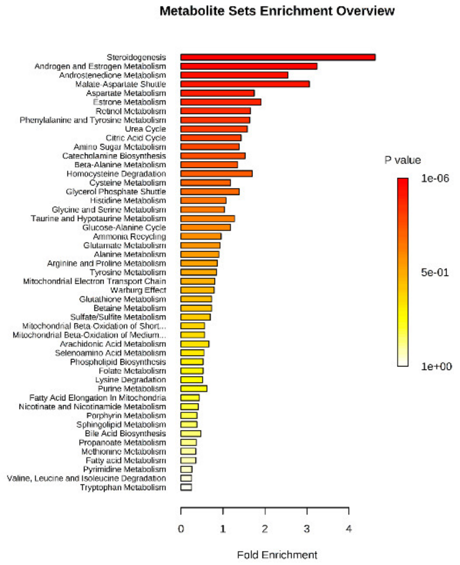

Figure S11. Summary plot for over-representation analysis (ORA) of blood plasma metabolites with an abnormal concentration in obese patients (stage 2 obesity in females). Other ORA related data is presented in Table S13.

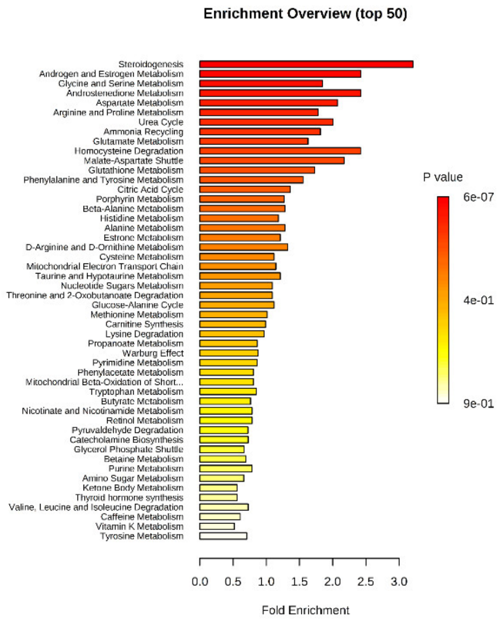

Figure S10. Summary plot for over-representation analysis (ORA) of blood plasma metabolites with an abnormal concentration in obese patients (stage 1 obesity in females). Other ORA related data is presented in Table S12.

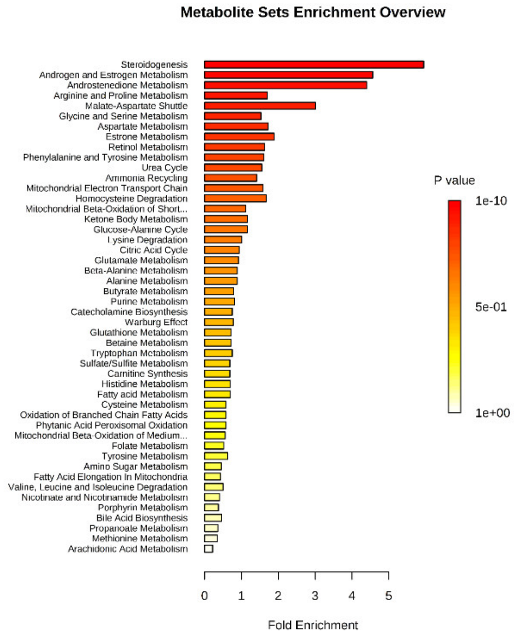

Figure S12. Summary plot for over-representation analysis (ORA) of blood plasma metabolites with an abnormal concentration in obese patients (stage 3 obesity in females). Other ORA related data is presented in Table S14.

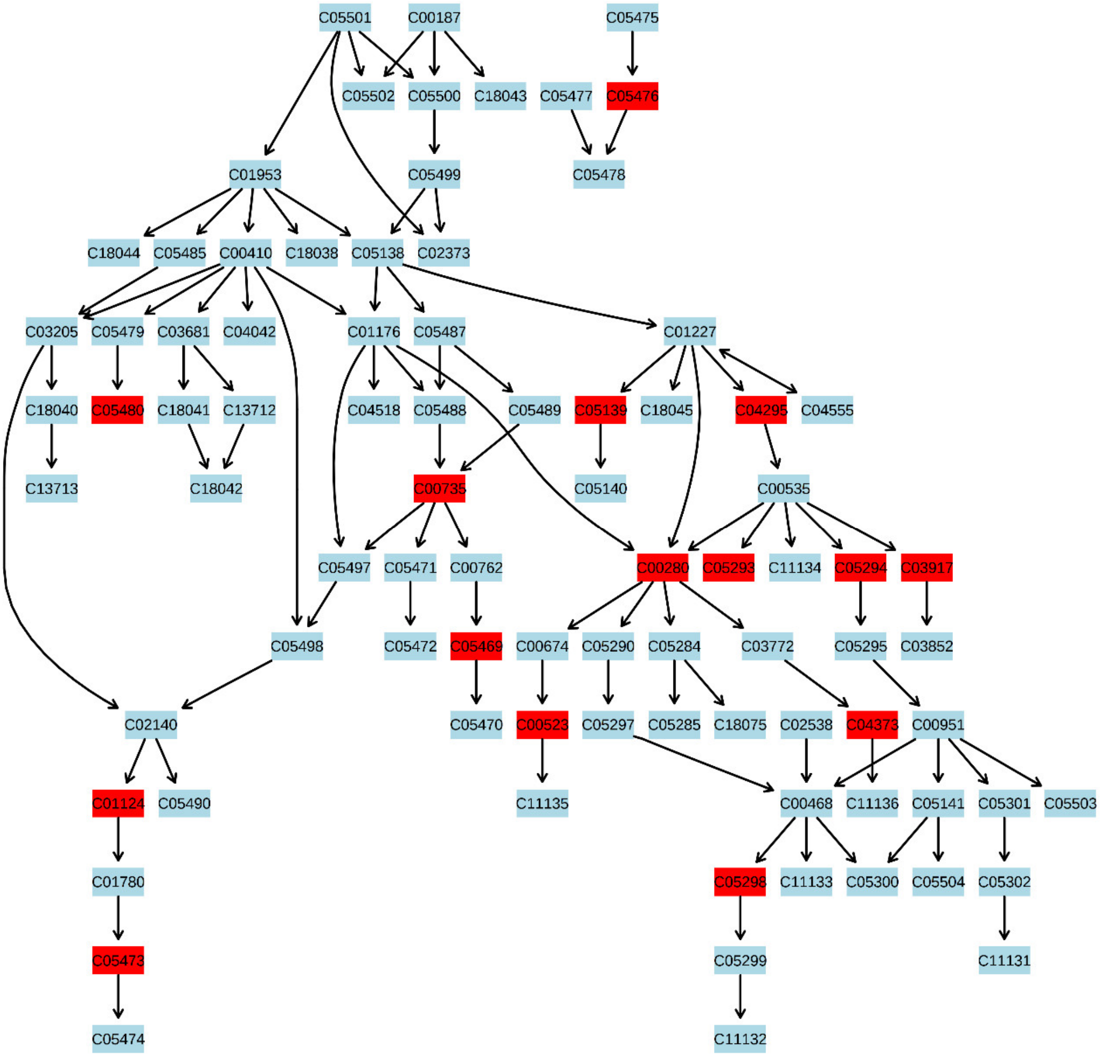

Figure S13. Projection of blood plasma metabolites with an abnormal concentration in males with stage 3 obesity on the steroid hormone biosynthesis pathway. Metabolites are labeled by KEGG identifiers. Altered metabolites are marked by red boxes. The image was generated using MetaboAnalyst software.

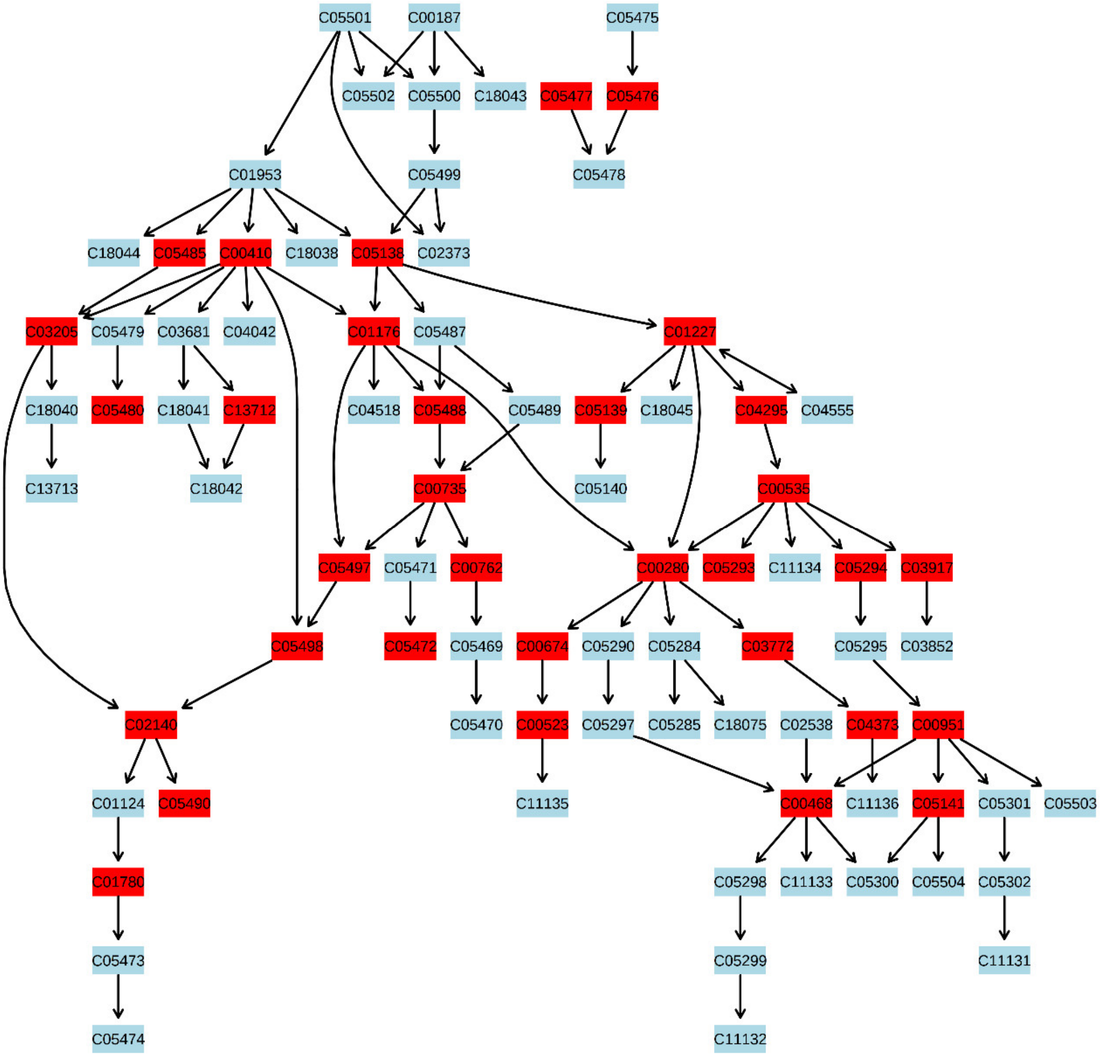

Figure S14. Projection of blood plasma metabolites with an abnormal concentration in females with stage 3 obesity on the steroid hormone biosynthesis pathway. Metabolites are labeled by KEGG identifiers. Altered metabolites are marked by red boxes. The image was generated using MetaboAnalyst software.

Table S3. Data related to the over-representation analysis (ORA) of blood plasma metabolites with an abnormal concentration in overweight males and females, presented in Figure S1.

	total	expected	hits	Raw p	Holm p	FDR
Steroidogenesis	43	3.57	14	2.80E-06	2.74E-04	2.74E-04
Androstenedione Metabolism	24	1.99	8	3.98E-04	3.86E-02	1.95E-02
Androgen and Estrogen Metabolism	33	2.74	9	9.02E-04	8.66E-02	2.95E-02
Aspartate Metabolism	35	2.91	6	6.27E-02	1.00E+00	1.00E+00
Urea Cycle	29	2.41	5	8.50E-02	1.00E+00	1.00E+00
Ammonia Recycling	32	2.66	5	1.19E-01	1.00E+00	1.00E+00
Estrone Metabolism	24	1.99	4	1.31E-01	1.00E+00	1.00E+00
Beta-Alanine Metabolism	34	2.82	5	1.44E-01	1.00E+00	1.00E+00
Malate-Aspartate Shuttle	10	0.83	2	1.99E-01	1.00E+00	1.00E+00
Glucose-Alanine Cycle	13	1.08	2	2.94E-01	1.00E+00	1.00E+00
Glycine and Serine Metabolism	59	4.90	6	3.64E-01	1.00E+00	1.00E+00
Cysteine Metabolism	26	2.16	3	3.68E-01	1.00E+00	1.00E+00
Retinol Metabolism	37	3.07	4	3.69E-01	1.00E+00	1.00E+00
Tryptophan Metabolism	60	4.98	6	3.80E-01	1.00E+00	1.00E+00
Glutamate Metabolism	49	4.07	5	3.85E-01	1.00E+00	1.00E+00
Phenylalanine and Tyrosine Metabolism	28	2.32	3	4.15E-01	1.00E+00	1.00E+00
Alanine Metabolism	17	1.41	2	4.19E-01	1.00E+00	1.00E+00
Lysine Degradation	30	2.49	3	4.61E-01	1.00E+00	1.00E+00
Histidine Metabolism	43	3.57	4	4.86E-01	1.00E+00	1.00E+00
Citric Acid Cycle	32	2.66	3	5.05E-01	1.00E+00	1.00E+00
Warburg Effect	58	4.81	5	5.37E-01	1.00E+00	1.00E+00
Phenylacetate Metabolism	9	0.75	1	5.43E-01	1.00E+00	1.00E+00
Nicotinate and Nicotinamide Metabolism	37	3.07	3	6.07E-01	1.00E+00	1.00E+00
D-Arginine and D-Ornithine Metabolism	11	0.91	1	6.16E-01	1.00E+00	1.00E+00
Taurine and Hypotaurine Metabolism	12	1.00	1	6.49E-01	1.00E+00	1.00E+00
Arginine and Proline Metabolism	53	4.40	4	6.57E-01	1.00E+00	1.00E+00
Porphyrin Metabolism	40	3.32	3	6.61E-01	1.00E+00	1.00E+00
Folate Metabolism	29	2.41	2	7.11E-01	1.00E+00	1.00E+00
Pyrimidine Metabolism	59	4.90	4	7.40E-01	1.00E+00	1.00E+00
Amino Sugar Metabolism	33	2.74	2	7.76E-01	1.00E+00	1.00E+00
Gluconeogenesis	35	2.91	2	8.04E-01	1.00E+00	1.00E+00
Mitochondrial Electron Transport Chain	19	1.58	1	8.10E-01	1.00E+00	1.00E+00
Nucleotide Sugars Metabolism	20	1.66	1	8.26E-01	1.00E+00	1.00E+00
Catecholamine Biosynthesis	20	1.66	1	8.26E-01	1.00E+00	1.00E+00
Glutathione Metabolism	21	1.74	1	8.41E-01	1.00E+00	1.00E+00
Sulfate/Sulfite Metabolism	22	1.83	1	8.54E-01	1.00E+00	1.00E+00
Carnitine Synthesis	22	1.83	1	8.54E-01	1.00E+00	1.00E+00
Sphingolipid Metabolism	40	3.32	2	8.61E-01	1.00E+00	1.00E+00
Tyrosine Metabolism	72	5.98	4	8.68E-01	1.00E+00	1.00E+00
Propanoate Metabolism	42	3.49	2	8.79E-01	1.00E+00	1.00E+00
Purine Metabolism	74	6.14	4	8.81E-01	1.00E+00	1.00E+00
Glycolysis	25	2.08	1	8.88E-01	1.00E+00	1.00E+00
Oxidation of Branched Chain Fatty Acids	26	2.16	1	8.98E-01	1.00E+00	1.00E+00
Phytanic Acid Peroxisomal Oxidation	26	2.16	1	8.98E-01	1.00E+00	1.00E+00
Pentose Phosphate Pathway	29	2.41	1	9.22E-01	1.00E+00	1.00E+00
Starch and Sucrose Metabolism	31	2.57	1	9.35E-01	1.00E+00	1.00E+00
Arachidonic Acid Metabolism	69	5.73	3	9.39E-01	1.00E+00	1.00E+00
Valine, Leucine and Isoleucine Degradation	60	4.98	2	9.68E-01	1.00E+00	1.00E+00
Bile Acid Biosynthesis	65	5.40	1	9.97E-01	1.00E+00	1.00E+00

Tables S3-S14 were generated by MetaboAnalyst.

Table S4. Data related to the over-representation analysis (ORA) of blood plasma metabolites with an abnormal concentration in obese patients (stage 1 obesity in males and females), presented in Figure S2.

	total	expected	hits	Raw p	Holm p	FDR
Steroidogenesis	43	6.09	17	2.37E-05	2.32E-03	2.32E-03
Glycine and Serine Metabolism	59	8.35	16	5.27E-03	5.11E-01	2.58E-01
Androgen and Estrogen Metabolism	33	4.67	10	1.19E-02	1.00E+00	3.24E-01
Androstenedione Metabolism	24	3.40	8	1.32E-02	1.00E+00	3.24E-01
Arginine and Proline Metabolism	53	7.50	13	2.74E-02	1.00E+00	5.36E-01
Glutamate Metabolism	49	6.94	12	3.40E-02	1.00E+00	5.55E-01
Urea Cycle	29	4.11	8	4.15E-02	1.00E+00	5.80E-01
Aspartate Metabolism	35	4.96	9	4.77E-02	1.00E+00	5.84E-01
Homocysteine Degradation	9	1.27	3	1.23E-01	1.00E+00	1.00E+00
Ammonia Recycling	32	4.53	7	1.54E-01	1.00E+00	1.00E+00
Malate-Aspartate Shuttle	10	1.42	3	1.58E-01	1.00E+00	1.00E+00
Glutathione Metabolism	21	2.97	5	1.64E-01	1.00E+00	1.00E+00
Alanine Metabolism	17	2.41	4	2.11E-01	1.00E+00	1.00E+00
Glucose-Alanine Cycle	13	1.84	3	2.76E-01	1.00E+00	1.00E+00
Estrone Metabolism	24	3.40	4	4.49E-01	1.00E+00	1.00E+00
Citric Acid Cycle	32	4.53	5	4.83E-01	1.00E+00	1.00E+00
Porphyrin Metabolism	40	5.66	6	5.09E-01	1.00E+00	1.00E+00
Amino Sugar Metabolism	33	4.67	5	5.12E-01	1.00E+00	1.00E+00
Cysteine Metabolism	26	3.68	4	5.15E-01	1.00E+00	1.00E+00
Taurine and Hypotaurine Metabolism	12	1.70	2	5.24E-01	1.00E+00	1.00E+00
Catecholamine Biosynthesis	20	2.83	3	5.55E-01	1.00E+00	1.00E+00
Selenoamino Acid Metabolism	28	3.96	4	5.77E-01	1.00E+00	1.00E+00
Tyrosine Metabolism	72	10.20	10	5.83E-01	1.00E+00	1.00E+00
Histidine Metabolism	43	6.09	6	5.85E-01	1.00E+00	1.00E+00
Carnitine Synthesis	22	3.12	3	6.23E-01	1.00E+00	1.00E+00
Beta-Alanine Metabolism	34	4.81	4	7.33E-01	1.00E+00	1.00E+00
Phenylacetate Metabolism	9	1.27	1	7.48E-01	1.00E+00	1.00E+00
Methionine Metabolism	43	6.09	5	7.53E-01	1.00E+00	1.00E+00
Tryptophan Metabolism	60	8.50	7	7.72E-01	1.00E+00	1.00E+00
Butyrate Metabolism	19	2.69	2	7.76E-01	1.00E+00	1.00E+00
Mitochondrial Electron Transport Chain	19	2.69	2	7.76E-01	1.00E+00	1.00E+00
Phenylalanine and Tyrosine Metabolism	28	3.96	3	7.83E-01	1.00E+00	1.00E+00
Retinol Metabolism	37	5.24	4	7.94E-01	1.00E+00	1.00E+00
Nucleotide Sugars Metabolism	20	2.83	2	8.00E-01	1.00E+00	1.00E+00
Threonine and 2-Oxobutanoate Degradation	20	2.83	2	8.00E-01	1.00E+00	1.00E+00
D-Arginine and D-Ornithine Metabolism	11	1.56	1	8.15E-01	1.00E+00	1.00E+00
Lysine Degradation	30	4.25	3	8.22E-01	1.00E+00	1.00E+00
Warburg Effect	58	8.21	6	8.55E-01	1.00E+00	1.00E+00
Ketone Body Metabolism	13	1.84	1	8.64E-01	1.00E+00	1.00E+00
Propanoate Metabolism	42	5.95	4	8.69E-01	1.00E+00	1.00E+00
Valine, Leucine and Isoleucine Degradation	60	8.50	6	8.77E-01	1.00E+00	1.00E+00
Oxidation of Branched Chain Fatty Acids	26	3.68	2	9.03E-01	1.00E+00	1.00E+00
Phytanic Acid Peroxisomal Oxidation	26	3.68	2	9.03E-01	1.00E+00	1.00E+00
Mitochondrial Beta-Oxidation of Short Chain Saturated Fatty Acids	27	3.82	2	9.15E-01	1.00E+00	1.00E+00
Nicotinate and Nicotinamide Metabolism	37	5.24	3	9.15E-01	1.00E+00	1.00E+00
Folate Metabolism	29	4.11	2	9.34E-01	1.00E+00	1.00E+00
Spermidine and Spermine Biosynthesis	18	2.55	1	9.38E-01	1.00E+00	1.00E+00
Pyrimidine Metabolism	59	8.35	5	9.39E-01	1.00E+00	1.00E+00
Starch and Sucrose Metabolism	31	4.39	2	9.49E-01	1.00E+00	1.00E+00
Riboflavin Metabolism	20	2.83	1	9.54E-01	1.00E+00	1.00E+00
Lactose Synthesis	20	2.83	1	9.54E-01	1.00E+00	1.00E+00
Pantothenate and CoA Biosynthesis	21	2.97	1	9.61E-01	1.00E+00	1.00E+00
Betaine Metabolism	21	2.97	1	9.61E-01	1.00E+00	1.00E+00
Purine Metabolism	74	10.50	6	9.65E-01	1.00E+00	1.00E+00
Sulfate/Sulfite Metabolism	22	3.12	1	9.67E-01	1.00E+00	1.00E+00
Transfer of Acetyl Groups into Mitochondria	22	3.12	1	9.67E-01	1.00E+00	1.00E+00
Glycerolipid Metabolism	25	3.54	1	9.79E-01	1.00E+00	1.00E+00
Sphingolipid Metabolism	40	5.66	2	9.85E-01	1.00E+00	1.00E+00
Pterine Biosynthesis	29	4.11	1	9.89E-01	1.00E+00	1.00E+00
Pentose Phosphate Pathway	29	4.11	1	9.89E-01	1.00E+00	1.00E+00
Bile Acid Biosynthesis	65	9.20	4	9.89E-01	1.00E+00	1.00E+00
Pyruvate Metabolism	48	6.80	2	9.95E-01	1.00E+00	1.00E+00
Fatty Acid Elongation In Mitochondria	35	4.96	1	9.96E-01	1.00E+00	1.00E+00
Gluconeogenesis	35	4.96	1	9.96E-01	1.00E+00	1.00E+00
Galactose Metabolism	38	5.38	1	9.97E-01	1.00E+00	1.00E+00
Arachidonic Acid Metabolism	69	9.77	3	9.98E-01	1.00E+00	1.00E+00
Fatty acid Metabolism	43	6.09	1	9.99E-01	1.00E+00	1.00E+00

Table S5. Data related to the over-representation analysis (ORA) of blood plasma metabolites with an abnormal concentration in obese patients (stage 2 obesity in males and females) , presented in Figure S3.

	total	expected	hits	Raw p	Holm p	FDR
Steroidogenesis	43	5.12	19	5.05E-08	4.95E-06	4.95E-06
Androgen and Estrogen Metabolism	33	3.93	9	1.15E-02	1.00E+00	5.63E-01
Androstenedione Metabolism	24	2.86	7	1.75E-02	1.00E+00	5.71E-01
Glycine and Serine Metabolism	59	7.03	12	3.87E-02	1.00E+00	9.20E-01
Aspartate Metabolism	35	4.17	8	4.70E-02	1.00E+00	9.20E-01
Malate-Aspartate Shuttle	10	1.19	3	1.06E-01	1.00E+00	1.00E+00
Urea Cycle	29	3.46	6	1.20E-01	1.00E+00	1.00E+00
Arginine and Proline Metabolism	53	6.31	9	1.69E-01	1.00E+00	1.00E+00
Ammonia Recycling	32	3.81	6	1.71E-01	1.00E+00	1.00E+00
Glutamate Metabolism	49	5.84	8	2.19E-01	1.00E+00	1.00E+00
Homocysteine Degradation	9	1.07	2	2.92E-01	1.00E+00	1.00E+00
Alanine Metabolism	17	2.03	3	3.30E-01	1.00E+00	1.00E+00
Cysteine Metabolism	26	3.10	4	3.76E-01	1.00E+00	1.00E+00
Beta-Alanine Metabolism	34	4.05	5	3.81E-01	1.00E+00	1.00E+00
Histidine Metabolism	43	5.12	6	4.07E-01	1.00E+00	1.00E+00
Phenylalanine and Tyrosine Metabolism	28	3.34	4	4.33E-01	1.00E+00	1.00E+00
Glucose-Alanine Cycle	13	1.55	2	4.71E-01	1.00E+00	1.00E+00
Carnitine Synthesis	22	2.62	3	4.98E-01	1.00E+00	1.00E+00
Citric Acid Cycle	32	3.81	4	5.42E-01	1.00E+00	1.00E+00
Estrone Metabolism	24	2.86	3	5.60E-01	1.00E+00	1.00E+00
Retinol Metabolism	37	4.41	4	6.62E-01	1.00E+00	1.00E+00
Mitochondrial Electron Transport Chain	19	2.26	2	6.82E-01	1.00E+00	1.00E+00
Thiamine Metabolism	9	1.07	1	6.82E-01	1.00E+00	1.00E+00
Catecholamine Biosynthesis	20	2.38	2	7.10E-01	1.00E+00	1.00E+00
Lysine Degradation	30	3.57	3	7.15E-01	1.00E+00	1.00E+00
Glutathione Metabolism	21	2.50	2	7.36E-01	1.00E+00	1.00E+00
D-Arginine and D-Ornithine Metabolism	11	1.31	1	7.54E-01	1.00E+00	1.00E+00
Amino Sugar Metabolism	33	3.93	3	7.75E-01	1.00E+00	1.00E+00
Taurine and Hypotaurine Metabolism	12	1.43	1	7.84E-01	1.00E+00	1.00E+00
Ketone Body Metabolism	13	1.55	1	8.10E-01	1.00E+00	1.00E+00
Oxidation of Branched Chain Fatty Acids	26	3.10	2	8.37E-01	1.00E+00	1.00E+00
Phytanic Acid Peroxisomal Oxidation	26	3.10	2	8.37E-01	1.00E+00	1.00E+00
Nicotinate and Nicotinamide Metabolism	37	4.41	3	8.39E-01	1.00E+00	1.00E+00
Warburg Effect	58	6.91	5	8.44E-01	1.00E+00	1.00E+00
Tryptophan Metabolism	60	7.15	5	8.65E-01	1.00E+00	1.00E+00
Selenoamino Acid Metabolism	28	3.34	2	8.66E-01	1.00E+00	1.00E+00
Porphyrin Metabolism	40	4.77	3	8.76E-01	1.00E+00	1.00E+00
Folate Metabolism	29	3.46	2	8.79E-01	1.00E+00	1.00E+00
Propanoate Metabolism	42	5.00	3	8.96E-01	1.00E+00	1.00E+00
Purine Metabolism	74	8.82	6	8.97E-01	1.00E+00	1.00E+00
Methionine Metabolism	43	5.12	3	9.05E-01	1.00E+00	1.00E+00
Butyrate Metabolism	19	2.26	1	9.12E-01	1.00E+00	1.00E+00
Threonine and 2-Oxobutanoate Degradation	20	2.38	1	9.23E-01	1.00E+00	1.00E+00
Betaine Metabolism	21	2.50	1	9.32E-01	1.00E+00	1.00E+00
Gluconeogenesis	35	4.17	2	9.36E-01	1.00E+00	1.00E+00
Sulfate/Sulfite Metabolism	22	2.62	1	9.41E-01	1.00E+00	1.00E+00
Valine, Leucine and Isoleucine Degradation	60	7.15	4	9.43E-01	1.00E+00	1.00E+00
Tyrosine Metabolism	72	8.58	5	9.47E-01	1.00E+00	1.00E+00
Glycerolipid Metabolism	25	2.98	1	9.60E-01	1.00E+00	1.00E+00
Glycolysis	25	2.98	1	9.60E-01	1.00E+00	1.00E+00
Sphingolipid Metabolism	40	4.77	2	9.63E-01	1.00E+00	1.00E+00
Pterine Biosynthesis	29	3.46	1	9.76E-01	1.00E+00	1.00E+00
Pyrimidine Metabolism	59	7.03	3	9.80E-01	1.00E+00	1.00E+00
Bile Acid Biosynthesis	65	7.74	3	9.89E-01	1.00E+00	1.00E+00
Arachidonic Acid Metabolism	69	8.22	3	9.93E-01	1.00E+00	1.00E+00

Table S6. Data related to the over-representation analysis (ORA) of blood plasma metabolites with an abnormal concentration in obese patients (stage 3 obesity in males and females) , presented in Figure S4.

	total	expected	hits	Raw p	Holm p	FDR
Steroidogenesis	43	4.96	23	4.74E-12	4.64E-10	4.64E-10
Androgen and Estrogen Metabolism	33	3.80	10	2.60E-03	2.52E-01	1.28E-01
Androstenedione Metabolism	24	2.77	7	1.46E-02	1.00E+00	4.78E-01
Glycine and Serine Metabolism	59	6.80	10	1.30E-01	1.00E+00	1.00E+00
Homocysteine Degradation	9	1.04	2	2.78E-01	1.00E+00	1.00E+00
Malate-Aspartate Shuttle	10	1.15	2	3.23E-01	1.00E+00	1.00E+00
Cysteine Metabolism	26	3.00	4	3.51E-01	1.00E+00	1.00E+00
Beta-Alanine Metabolism	34	3.92	5	3.53E-01	1.00E+00	1.00E+00
Aspartate Metabolism	35	4.03	5	3.78E-01	1.00E+00	1.00E+00
Taurine and Hypotaurine Metabolism	12	1.38	2	4.11E-01	1.00E+00	1.00E+00
Retinol Metabolism	37	4.26	5	4.26E-01	1.00E+00	1.00E+00
Glutathione Metabolism	21	2.42	3	4.43E-01	1.00E+00	1.00E+00
Glutamate Metabolism	49	5.65	6	5.05E-01	1.00E+00	1.00E+00
Ammonia Recycling	32	3.69	4	5.14E-01	1.00E+00	1.00E+00
Estrone Metabolism	24	2.77	3	5.36E-01	1.00E+00	1.00E+00
Amino Sugar Metabolism	33	3.80	4	5.40E-01	1.00E+00	1.00E+00
Tryptophan Metabolism	60	6.91	7	5.50E-01	1.00E+00	1.00E+00
Histidine Metabolism	43	4.96	5	5.65E-01	1.00E+00	1.00E+00
Arginine and Proline Metabolism	53	6.11	6	5.87E-01	1.00E+00	1.00E+00
Mitochondrial Beta-Oxidation of Short Chain Saturated Fatty Acids	27	3.11	3	6.19E-01	1.00E+00	1.00E+00
Phenylalanine and Tyrosine Metabolism	28	3.23	3	6.45E-01	1.00E+00	1.00E+00
Urea Cycle	29	3.34	3	6.69E-01	1.00E+00	1.00E+00
Catecholamine Biosynthesis	20	2.30	2	6.91E-01	1.00E+00	1.00E+00
Lysine Degradation	30	3.46	3	6.92E-01	1.00E+00	1.00E+00
Citric Acid Cycle	32	3.69	3	7.34E-01	1.00E+00	1.00E+00
Ketone Body Metabolism	13	1.50	1	7.98E-01	1.00E+00	1.00E+00
Glucose-Alanine Cycle	13	1.50	1	7.98E-01	1.00E+00	1.00E+00
Nicotinate and Nicotinamide Metabolism	37	4.26	3	8.21E-01	1.00E+00	1.00E+00
Warburg Effect	58	6.68	5	8.21E-01	1.00E+00	1.00E+00
Oxidation of Branched Chain Fatty Acids	26	3.00	2	8.22E-01	1.00E+00	1.00E+00
Phytanic Acid Peroxisomal Oxidation	26	3.00	2	8.22E-01	1.00E+00	1.00E+00
Mitochondrial Beta-Oxidation of Medium Chain Saturated Fatty Acids	27	3.11	2	8.38E-01	1.00E+00	1.00E+00
Beta Oxidation of Very Long Chain Fatty Acids	17	1.96	1	8.77E-01	1.00E+00	1.00E+00
Alanine Metabolism	17	1.96	1	8.77E-01	1.00E+00	1.00E+00
Propanoate Metabolism	42	4.84	3	8.82E-01	1.00E+00	1.00E+00
Fatty acid Metabolism	43	4.96	3	8.92E-01	1.00E+00	1.00E+00
Butyrate Metabolism	19	2.19	1	9.04E-01	1.00E+00	1.00E+00
Mitochondrial Electron Transport Chain	19	2.19	1	9.04E-01	1.00E+00	1.00E+00
Ethanol Degradation	19	2.19	1	9.04E-01	1.00E+00	1.00E+00
Nucleotide Sugars Metabolism	20	2.30	1	9.16E-01	1.00E+00	1.00E+00
Threonine and 2-Oxobutanoate Degradation	20	2.30	1	9.16E-01	1.00E+00	1.00E+00
Pantothenate and CoA Biosynthesis	21	2.42	1	9.26E-01	1.00E+00	1.00E+00
Betaine Metabolism	21	2.42	1	9.26E-01	1.00E+00	1.00E+00
Pyrimidine Metabolism	59	6.80	4	9.26E-01	1.00E+00	1.00E+00
Fatty Acid Elongation In Mitochondria	35	4.03	2	9.27E-01	1.00E+00	1.00E+00
Sulfate/Sulfite Metabolism	22	2.54	1	9.34E-01	1.00E+00	1.00E+00
Transfer of Acetyl Groups into Mitochondria	22	2.54	1	9.34E-01	1.00E+00	1.00E+00
Purine Metabolism	74	8.53	5	9.45E-01	1.00E+00	1.00E+00
Caffeine Metabolism	24	2.77	1	9.49E-01	1.00E+00	1.00E+00
Glycerolipid Metabolism	25	2.88	1	9.55E-01	1.00E+00	1.00E+00
Glycolysis	25	2.88	1	9.55E-01	1.00E+00	1.00E+00
Porphyrin Metabolism	40	4.61	2	9.57E-01	1.00E+00	1.00E+00
Sphingolipid Metabolism	40	4.61	2	9.57E-01	1.00E+00	1.00E+00
Methionine Metabolism	43	4.96	2	9.68E-01	1.00E+00	1.00E+00
Mitochondrial Beta-Oxidation of Long Chain Saturated Fatty Acids	28	3.23	1	9.69E-01	1.00E+00	1.00E+00
Pentose Phosphate Pathway	29	3.34	1	9.73E-01	1.00E+00	1.00E+00
Folate Metabolism	29	3.34	1	9.73E-01	1.00E+00	1.00E+00
Tyrosine Metabolism	72	8.30	4	9.76E-01	1.00E+00	1.00E+00
Valine, Leucine and Isoleucine Degradation	60	6.91	3	9.78E-01	1.00E+00	1.00E+00
Bile Acid Biosynthesis	65	7.49	3	9.87E-01	1.00E+00	1.00E+00
Gluconeogenesis	35	4.03	1	9.87E-01	1.00E+00	1.00E+00
Fatty Acid Biosynthesis	35	4.03	1	9.87E-01	1.00E+00	1.00E+00
Arachidonic Acid Metabolism	69	7.95	3	9.91E-01	1.00E+00	1.00E+00
Steroid Biosynthesis	48	5.53	1	9.98E-01	1.00E+00	1.00E+00
Pyruvate Metabolism	48	5.53	1	9.98E-01	1.00E+00	1.00E+00

Table S7. Data related to the over-representation analysis (ORA) of blood plasma metabolites with an abnormal concentration in overweight males, presented in Figure S5.

	total	expected	hits	Raw p	Holm p	FDR
Beta-Alanine Metabolism	34	0.50	3	1.16E-02	1.00E+00	1.00E+00
Malate-Aspartate Shuttle	10	0.15	1	1.38E-01	1.00E+00	1.00E+00
Catecholamine Biosynthesis	20	0.29	1	2.58E-01	1.00E+00	1.00E+00
Tyrosine Metabolism	72	1.05	2	2.85E-01	1.00E+00	1.00E+00
Purine Metabolism	74	1.08	2	2.96E-01	1.00E+00	1.00E+00
Pterine Biosynthesis	29	0.42	1	3.52E-01	1.00E+00	1.00E+00
Urea Cycle	29	0.42	1	3.52E-01	1.00E+00	1.00E+00
Ammonia Recycling	32	0.47	1	3.81E-01	1.00E+00	1.00E+00
Aspartate Metabolism	35	0.51	1	4.09E-01	1.00E+00	1.00E+00
Porphyrin Metabolism	40	0.59	1	4.52E-01	1.00E+00	1.00E+00
Sphingolipid Metabolism	40	0.59	1	4.52E-01	1.00E+00	1.00E+00
Steroidogenesis	43	0.63	1	4.77E-01	1.00E+00	1.00E+00
Glutamate Metabolism	49	0.72	1	5.23E-01	1.00E+00	1.00E+00
Arginine and Proline Metabolism	53	0.78	1	5.52E-01	1.00E+00	1.00E+00
Pyrimidine Metabolism	59	0.86	1	5.92E-01	1.00E+00	1.00E+00
Tryptophan Metabolism	60	0.88	1	5.98E-01	1.00E+00	1.00E+00

Table S8. Data related to the over-representation analysis (ORA) of blood plasma metabolites with an abnormal concentration in obese patients (stage 1 obesity in males), presented in Figure S6.

	total	expected	hits	Raw p	Holm p	FDR
Phenylalanine and Tyrosine Metabolism	28	0.22	2	1.82E-02	1.00E+00	9.56E-01
Urea Cycle	29	0.23	2	1.95E-02	1.00E+00	9.56E-01
Arginine and Proline Metabolism	53	0.41	2	6.02E-02	1.00E+00	1.00E+00
Valine, Leucine and Isoleucine Degradation	60	0.47	2	7.53E-02	1.00E+00	1.00E+00
Malate-Aspartate Shuttle	10	0.08	1	7.58E-02	1.00E+00	1.00E+00
D-Arginine and D-Ornithine Metabolism	11	0.09	1	8.30E-02	1.00E+00	1.00E+00
Glucose-Alanine Cycle	13	0.10	1	9.75E-02	1.00E+00	1.00E+00
Tyrosine Metabolism	72	0.56	2	1.04E-01	1.00E+00	1.00E+00
Alanine Metabolism	17	0.13	1	1.26E-01	1.00E+00	1.00E+00
Glutathione Metabolism	21	0.16	1	1.53E-01	1.00E+00	1.00E+00
Androstenedione Metabolism	24	0.19	1	1.73E-01	1.00E+00	1.00E+00
Cysteine Metabolism	26	0.20	1	1.87E-01	1.00E+00	1.00E+00
Selenoamino Acid Metabolism	28	0.22	1	2.00E-01	1.00E+00	1.00E+00
Folate Metabolism	29	0.23	1	2.06E-01	1.00E+00	1.00E+00
Lysine Degradation	30	0.23	1	2.12E-01	1.00E+00	1.00E+00
Ammonia Recycling	32	0.25	1	2.25E-01	1.00E+00	1.00E+00
Amino Sugar Metabolism	33	0.26	1	2.31E-01	1.00E+00	1.00E+00
Beta-Alanine Metabolism	34	0.27	1	2.37E-01	1.00E+00	1.00E+00
Aspartate Metabolism	35	0.27	1	2.44E-01	1.00E+00	1.00E+00
Nicotinate and Nicotinamide Metabolism	37	0.29	1	2.56E-01	1.00E+00	1.00E+00
Propanoate Metabolism	42	0.33	1	2.86E-01	1.00E+00	1.00E+00
Histidine Metabolism	43	0.34	1	2.91E-01	1.00E+00	1.00E+00
Glutamate Metabolism	49	0.38	1	3.25E-01	1.00E+00	1.00E+00
Warburg Effect	58	0.45	1	3.74E-01	1.00E+00	1.00E+00
Glycine and Serine Metabolism	59	0.46	1	3.79E-01	1.00E+00	1.00E+00
Tryptophan Metabolism	60	0.47	1	3.84E-01	1.00E+00	1.00E+00
Arachidonic Acid Metabolism	69	0.54	1	4.29E-01	1.00E+00	1.00E+00
Purine Metabolism	74	0.58	1	4.52E-01	1.00E+00	1.00E+00

Table S9. Data related to the over-representation analysis (ORA) of blood plasma metabolites with an abnormal concentration in obese patients (stage 2 obesity in males), presented in Figure S7.

	total	expected	hits	Raw p	Holm p	FDR
Steroidogenesis	43	2.14	7	4.03E-03	3.95E-01	3.95E-01
Androgen and Estrogen Metabolism	33	1.64	4	7.67E-02	1.00E+00	1.00E+00
Androstenedione Metabolism	24	1.20	3	1.13E-01	1.00E+00	1.00E+00
Ammonia Recycling	32	1.59	3	2.10E-01	1.00E+00	1.00E+00
Amino Sugar Metabolism	33	1.64	3	2.24E-01	1.00E+00	1.00E+00
Aspartate Metabolism	35	1.74	3	2.51E-01	1.00E+00	1.00E+00
Retinol Metabolism	37	1.84	3	2.79E-01	1.00E+00	1.00E+00
Glycine and Serine Metabolism	59	2.94	4	3.38E-01	1.00E+00	1.00E+00
Histidine Metabolism	43	2.14	3	3.63E-01	1.00E+00	1.00E+00
Cysteine Metabolism	26	1.29	2	3.75E-01	1.00E+00	1.00E+00
Malate-Aspartate Shuttle	10	0.50	1	4.01E-01	1.00E+00	1.00E+00
Phenylalanine and Tyrosine Metabolism	28	1.39	2	4.11E-01	1.00E+00	1.00E+00
Urea Cycle	29	1.44	2	4.29E-01	1.00E+00	1.00E+00
Lysine Degradation	30	1.49	2	4.47E-01	1.00E+00	1.00E+00
Taurine and Hypotaurine Metabolism	12	0.60	1	4.60E-01	1.00E+00	1.00E+00
Glucose-Alanine Cycle	13	0.65	1	4.87E-01	1.00E+00	1.00E+00
Alanine Metabolism	17	0.85	1	5.83E-01	1.00E+00	1.00E+00
Tryptophan Metabolism	60	2.99	3	5.86E-01	1.00E+00	1.00E+00
Porphyrin Metabolism	40	1.99	2	6.04E-01	1.00E+00	1.00E+00
Mitochondrial Electron Transport Chain	19	0.95	1	6.25E-01	1.00E+00	1.00E+00
Glutathione Metabolism	21	1.05	1	6.62E-01	1.00E+00	1.00E+00
Caffeine Metabolism	24	1.20	1	7.11E-01	1.00E+00	1.00E+00
Estrone Metabolism	24	1.20	1	7.11E-01	1.00E+00	1.00E+00
Glutamate Metabolism	49	2.44	2	7.15E-01	1.00E+00	1.00E+00
Purine Metabolism	74	3.69	3	7.30E-01	1.00E+00	1.00E+00
Arginine and Proline Metabolism	53	2.64	2	7.56E-01	1.00E+00	1.00E+00
Selenoamino Acid Metabolism	28	1.39	1	7.65E-01	1.00E+00	1.00E+00
Pterine Biosynthesis	29	1.44	1	7.77E-01	1.00E+00	1.00E+00
Folate Metabolism	29	1.44	1	7.77E-01	1.00E+00	1.00E+00
Warburg Effect	58	2.89	2	8.00E-01	1.00E+00	1.00E+00
Citric Acid Cycle	32	1.59	1	8.10E-01	1.00E+00	1.00E+00
Beta-Alanine Metabolism	34	1.69	1	8.29E-01	1.00E+00	1.00E+00
Nicotinate and Nicotinamide Metabolism	37	1.84	1	8.54E-01	1.00E+00	1.00E+00
Propanoate Metabolism	42	2.09	1	8.88E-01	1.00E+00	1.00E+00
Tyrosine Metabolism	72	3.59	2	8.88E-01	1.00E+00	1.00E+00
Valine, Leucine and Isoleucine Degradation	60	2.99	1	9.58E-01	1.00E+00	1.00E+00
Bile Acid Biosynthesis	65	3.24	1	9.68E-01	1.00E+00	1.00E+00
Arachidonic Acid Metabolism	69	3.44	1	9.74E-01	1.00E+00	1.00E+00

Table S10. Data related to the over-representation analysis (ORA) of blood plasma metabolites with an abnormal concentration in obese patients (stage 3 obesity in males), presented in Figure S8.

	total	expected	hits	Raw p	Holm p	FDR
Steroidogenesis	43	1.47	6	2.50E-03	2.45E-01	2.45E-01
Androgen and Estrogen Metabolism	33	1.13	4	2.31E-02	1.00E+00	1.00E+00
Androstenedione Metabolism	24	0.82	3	4.53E-02	1.00E+00	1.00E+00
Glycine and Serine Metabolism	59	2.02	4	1.37E-01	1.00E+00	1.00E+00
Porphyrin Metabolism	40	1.37	3	1.53E-01	1.00E+00	1.00E+00
Phenylalanine and Tyrosine Metabolism	28	0.96	2	2.48E-01	1.00E+00	1.00E+00
Urea Cycle	29	0.99	2	2.61E-01	1.00E+00	1.00E+00
Malate-Aspartate Shuttle	10	0.34	1	2.95E-01	1.00E+00	1.00E+00
Ammonia Recycling	32	1.09	2	3.00E-01	1.00E+00	1.00E+00
Warburg Effect	58	1.98	3	3.18E-01	1.00E+00	1.00E+00
Tryptophan Metabolism	60	2.05	3	3.37E-01	1.00E+00	1.00E+00
Aspartate Metabolism	35	1.20	2	3.38E-01	1.00E+00	1.00E+00
Glucose-Alanine Cycle	13	0.44	1	3.65E-01	1.00E+00	1.00E+00
Sphingolipid Metabolism	40	1.37	2	4.01E-01	1.00E+00	1.00E+00
Histidine Metabolism	43	1.47	2	4.38E-01	1.00E+00	1.00E+00
Alanine Metabolism	17	0.58	1	4.49E-01	1.00E+00	1.00E+00
Mitochondrial Electron Transport Chain	19	0.65	1	4.87E-01	1.00E+00	1.00E+00
Glutathione Metabolism	21	0.72	1	5.22E-01	1.00E+00	1.00E+00
Arginine and Proline Metabolism	53	1.81	2	5.51E-01	1.00E+00	1.00E+00
Caffeine Metabolism	24	0.82	1	5.70E-01	1.00E+00	1.00E+00
Estrone Metabolism	24	0.82	1	5.70E-01	1.00E+00	1.00E+00
Glycolysis	25	0.85	1	5.85E-01	1.00E+00	1.00E+00
Cysteine Metabolism	26	0.89	1	6.00E-01	1.00E+00	1.00E+00
Folate Metabolism	29	0.99	1	6.40E-01	1.00E+00	1.00E+00
Lysine Degradation	30	1.03	1	6.53E-01	1.00E+00	1.00E+00
Citric Acid Cycle	32	1.09	1	6.77E-01	1.00E+00	1.00E+00
Amino Sugar Metabolism	33	1.13	1	6.88E-01	1.00E+00	1.00E+00
Beta-Alanine Metabolism	34	1.16	1	6.99E-01	1.00E+00	1.00E+00
Gluconeogenesis	35	1.20	1	7.10E-01	1.00E+00	1.00E+00
Tyrosine Metabolism	72	2.46	2	7.21E-01	1.00E+00	1.00E+00
Nicotinate and Nicotinamide Metabolism	37	1.26	1	7.30E-01	1.00E+00	1.00E+00
Retinol Metabolism	37	1.26	1	7.30E-01	1.00E+00	1.00E+00
Purine Metabolism	74	2.53	2	7.35E-01	1.00E+00	1.00E+00
Propanoate Metabolism	42	1.44	1	7.75E-01	1.00E+00	1.00E+00
Pyruvate Metabolism	48	1.64	1	8.19E-01	1.00E+00	1.00E+00
Glutamate Metabolism	49	1.67	1	8.26E-01	1.00E+00	1.00E+00
Valine, Leucine and Isoleucine Degradation	60	2.05	1	8.84E-01	1.00E+00	1.00E+00
Arachidonic Acid Metabolism	69	2.36	1	9.17E-01	1.00E+00	1.00E+00

Table S11. Data related to the over-representation analysis (ORA) of blood plasma metabolites with an abnormal concentration in overweight females, presented in Figure S9.

	total	expected	hits	Raw p	Holm p	FDR
Arginine and Proline Metabolism	53	2.17	6	1.79E-02	1.00E+00	9.00E-01
Phenylalanine and Tyrosine Metabolism	28	1.15	4	2.45E-02	1.00E+00	9.00E-01
Urea Cycle	29	1.19	4	2.75E-02	1.00E+00	9.00E-01
Androgen and Estrogen Metabolism	33	1.35	4	4.21E-02	1.00E+00	1.00E+00
Steroidogenesis	43	1.76	4	9.45E-02	1.00E+00	1.00E+00
Glutathione Metabolism	21	0.86	2	2.12E-01	1.00E+00	1.00E+00
Glycine and Serine Metabolism	59	2.42	4	2.19E-01	1.00E+00	1.00E+00
Tryptophan Metabolism	60	2.46	4	2.28E-01	1.00E+00	1.00E+00
Androstenedione Metabolism	24	0.98	2	2.58E-01	1.00E+00	1.00E+00
Estrone Metabolism	24	0.98	2	2.58E-01	1.00E+00	1.00E+00
Selenoamino Acid Metabolism	28	1.15	2	3.20E-01	1.00E+00	1.00E+00
Malate-Aspartate Shuttle	10	0.41	1	3.43E-01	1.00E+00	1.00E+00
D-Arginine and D-Ornithine Metabolism	11	0.45	1	3.71E-01	1.00E+00	1.00E+00
Glucose-Alanine Cycle	13	0.53	1	4.22E-01	1.00E+00	1.00E+00
Thyroid hormone synthesis	13	0.53	1	4.22E-01	1.00E+00	1.00E+00
Aspartate Metabolism	35	1.44	2	4.26E-01	1.00E+00	1.00E+00
Alanine Metabolism	17	0.70	1	5.12E-01	1.00E+00	1.00E+00
Fatty acid Metabolism	43	1.76	2	5.36E-01	1.00E+00	1.00E+00
Mitochondrial Electron Transport Chain	19	0.78	1	5.52E-01	1.00E+00	1.00E+00
Catecholamine Biosynthesis	20	0.82	1	5.71E-01	1.00E+00	1.00E+00
Tyrosine Metabolism	72	2.95	3	5.79E-01	1.00E+00	1.00E+00
Glutamate Metabolism	49	2.01	2	6.09E-01	1.00E+00	1.00E+00
Mitochondrial Beta-Oxidation of Short Chain Saturated Fatty Acids	27	1.11	1	6.82E-01	1.00E+00	1.00E+00
Mitochondrial Beta-Oxidation of Medium Chain Saturated Fatty Acids	27	1.11	1	6.82E-01	1.00E+00	1.00E+00
Ammonia Recycling	32	1.31	1	7.44E-01	1.00E+00	1.00E+00
Citric Acid Cycle	32	1.31	1	7.44E-01	1.00E+00	1.00E+00
Beta-Alanine Metabolism	34	1.39	1	7.65E-01	1.00E+00	1.00E+00
Fatty Acid Elongation In Mitochondria	35	1.44	1	7.75E-01	1.00E+00	1.00E+00
Retinol Metabolism	37	1.52	1	7.94E-01	1.00E+00	1.00E+00
Porphyrin Metabolism	40	1.64	1	8.19E-01	1.00E+00	1.00E+00
Sphingolipid Metabolism	40	1.64	1	8.19E-01	1.00E+00	1.00E+00
Purine Metabolism	74	3.04	2	8.23E-01	1.00E+00	1.00E+00
Histidine Metabolism	43	1.76	1	8.41E-01	1.00E+00	1.00E+00
Warburg Effect	58	2.38	1	9.18E-01	1.00E+00	1.00E+00
Bile Acid Biosynthesis	65	2.67	1	9.40E-01	1.00E+00	1.00E+00

Table S12. Data related to the over-representation analysis (ORA) of blood plasma metabolites with an abnormal concentration in obese patients (stage 1 obesity in females), presented in Figure S10.

	total	expected	hits	Raw p	Holm p	FDR
Steroidogenesis	43	5.92	19	6.00E-07	5.88E-05	5.88E-05
Androgen and Estrogen Metabolism	33	4.54	11	2.96E-03	2.87E-01	1.45E-01
Glycine and Serine Metabolism	59	8.12	15	1.00E-02	9.63E-01	2.74E-01
Androstenedione Metabolism	24	3.30	8	1.12E-02	1.00E+00	2.74E-01
Aspartate Metabolism	35	4.82	10	1.52E-02	1.00E+00	2.98E-01
Arginine and Proline Metabolism	53	7.30	13	2.20E-02	1.00E+00	3.59E-01
Urea Cycle	29	3.99	8	3.57E-02	1.00E+00	5.00E-01
Ammonia Recycling	32	4.41	8	6.10E-02	1.00E+00	6.72E-01
Glutamate Metabolism	49	6.75	11	6.17E-02	1.00E+00	6.72E-01
Homocysteine Degradation	9	1.24	3	1.15E-01	1.00E+00	1.00E+00
Malate-Aspartate Shuttle	10	1.38	3	1.48E-01	1.00E+00	1.00E+00
Glutathione Metabolism	21	2.89	5	1.51E-01	1.00E+00	1.00E+00
Phenylalanine and Tyrosine Metabolism	28	3.86	6	1.77E-01	1.00E+00	1.00E+00
Citric Acid Cycle	32	4.41	6	2.71E-01	1.00E+00	1.00E+00
Porphyrin Metabolism	40	5.51	7	3.07E-01	1.00E+00	1.00E+00
Beta-Alanine Metabolism	34	4.68	6	3.22E-01	1.00E+00	1.00E+00
Histidine Metabolism	43	5.92	7	3.79E-01	1.00E+00	1.00E+00
Alanine Metabolism	17	2.34	3	4.22E-01	1.00E+00	1.00E+00
Estrone Metabolism	24	3.30	4	4.26E-01	1.00E+00	1.00E+00
D-Arginine and D-Ornithine Metabolism	11	1.51	2	4.60E-01	1.00E+00	1.00E+00
Cysteine Metabolism	26	3.58	4	4.91E-01	1.00E+00	1.00E+00
Mitochondrial Electron Transport Chain	19	2.62	3	4.98E-01	1.00E+00	1.00E+00
Taurine and Hypotaurine Metabolism	12	1.65	2	5.08E-01	1.00E+00	1.00E+00
Nucleotide Sugars Metabolism	20	2.75	3	5.35E-01	1.00E+00	1.00E+00
Threonine and 2-Oxobutanoate Degradation	20	2.75	3	5.35E-01	1.00E+00	1.00E+00
Glucose-Alanine Cycle	13	1.79	2	5.53E-01	1.00E+00	1.00E+00
Methionine Metabolism	43	5.92	6	5.56E-01	1.00E+00	1.00E+00
Carnitine Synthesis	22	3.03	3	6.03E-01	1.00E+00	1.00E+00
Lysine Degradation	30	4.13	4	6.11E-01	1.00E+00	1.00E+00
Propanoate Metabolism	42	5.78	5	7.09E-01	1.00E+00	1.00E+00
Warburg Effect	58	7.99	7	7.10E-01	1.00E+00	1.00E+00
Pyrimidine Metabolism	59	8.12	7	7.28E-01	1.00E+00	1.00E+00
Phenylacetate Metabolism	9	1.24	1	7.38E-01	1.00E+00	1.00E+00
Mitochondrial Beta-Oxidation of Short Chain Saturated Fatty Acids	27	3.72	3	7.42E-01	1.00E+00	1.00E+00
Tryptophan Metabolism	60	8.26	7	7.44E-01	1.00E+00	1.00E+00
Butyrate Metabolism	19	2.62	2	7.61E-01	1.00E+00	1.00E+00
Nicotinate and Nicotinamide Metabolism	37	5.09	4	7.74E-01	1.00E+00	1.00E+00
Retinol Metabolism	37	5.09	4	7.74E-01	1.00E+00	1.00E+00
Pyruvaldehyde Degradation	10	1.38	1	7.74E-01	1.00E+00	1.00E+00
Catecholamine Biosynthesis	20	2.75	2	7.86E-01	1.00E+00	1.00E+00
Glycerol Phosphate Shuttle	11	1.51	1	8.06E-01	1.00E+00	1.00E+00
Betaine Metabolism	21	2.89	2	8.09E-01	1.00E+00	1.00E+00
Purine Metabolism	74	10.20	8	8.26E-01	1.00E+00	1.00E+00
Amino Sugar Metabolism	33	4.54	3	8.56E-01	1.00E+00	1.00E+00
Ketone Body Metabolism	13	1.79	1	8.56E-01	1.00E+00	1.00E+00
Thyroid hormone synthesis	13	1.79	1	8.56E-01	1.00E+00	1.00E+00
Valine, Leucine and Isoleucine Degradation	60	8.26	6	8.59E-01	1.00E+00	1.00E+00
Caffeine Metabolism	24	3.30	2	8.65E-01	1.00E+00	1.00E+00
Vitamin K Metabolism	14	1.93	1	8.76E-01	1.00E+00	1.00E+00
Tyrosine Metabolism	72	9.91	7	8.91E-01	1.00E+00	1.00E+00
Oxidation of Branched Chain Fatty Acids	26	3.58	2	8.94E-01	1.00E+00	1.00E+00
Phytanic Acid Peroxisomal Oxidation	26	3.58	2	8.94E-01	1.00E+00	1.00E+00
Mitochondrial Beta-Oxidation of Medium Chain Saturated Fatty Acids	27	3.72	2	9.06E-01	1.00E+00	1.00E+00
Selenoamino Acid Metabolism	28	3.86	2	9.17E-01	1.00E+00	1.00E+00
Folate Metabolism	29	3.99	2	9.26E-01	1.00E+00	1.00E+00
Spermidine and Spermine Biosynthesis	18	2.48	1	9.32E-01	1.00E+00	1.00E+00
Arachidonic Acid Metabolism	69	9.50	6	9.34E-01	1.00E+00	1.00E+00
Starch and Sucrose Metabolism	31	4.27	2	9.42E-01	1.00E+00	1.00E+00
Vitamin B6 Metabolism	20	2.75	1	9.50E-01	1.00E+00	1.00E+00
Riboflavin Metabolism	20	2.75	1	9.50E-01	1.00E+00	1.00E+00
Lactose Synthesis	20	2.75	1	9.50E-01	1.00E+00	1.00E+00
Pantothenate and CoA Biosynthesis	21	2.89	1	9.57E-01	1.00E+00	1.00E+00
Bile Acid Biosynthesis	65	8.95	5	9.60E-01	1.00E+00	1.00E+00
Sulfate/Sulfite Metabolism	22	3.03	1	9.63E-01	1.00E+00	1.00E+00
Transfer of Acetyl Groups into Mitochondria	22	3.03	1	9.63E-01	1.00E+00	1.00E+00
Pyruvate Metabolism	48	6.61	3	9.72E-01	1.00E+00	1.00E+00
Glycerolipid Metabolism	25	3.44	1	9.77E-01	1.00E+00	1.00E+00
Plasmalogen Synthesis	26	3.58	1	9.80E-01	1.00E+00	1.00E+00
Sphingolipid Metabolism	40	5.51	2	9.82E-01	1.00E+00	1.00E+00
Mitochondrial Beta-Oxidation of Long Chain Saturated Fatty Acids	28	3.86	1	9.85E-01	1.00E+00	1.00E+00
Phospholipid Biosynthesis	29	3.99	1	9.87E-01	1.00E+00	1.00E+00
Pentose Phosphate Pathway	29	3.99	1	9.87E-01	1.00E+00	1.00E+00
Fatty acid Metabolism	43	5.92	2	9.88E-01	1.00E+00	1.00E+00
Fatty Acid Elongation In Mitochondria	35	4.82	1	9.95E-01	1.00E+00	1.00E+00
Gluconeogenesis	35	4.82	1	9.95E-01	1.00E+00	1.00E+00
Galactose Metabolism	38	5.23	1	9.97E-01	1.00E+00	1.00E+00
Steroid Biosynthesis	48	6.61	1	9.99E-01	1.00E+00	1.00E+00

Table S13. Data related to the over-representation analysis (ORA) of blood plasma metabolites with an abnormal concentration in obese patients (stage 2 obesity in females), presented in Figure S11.

	total	expected	hits	Raw p	Holm p	FDR
Steroidogenesis	43	2.81	13	9.82E-07	9.62E-05	9.62E-05
Androgen and Estrogen Metabolism	33	2.16	7	4.09E-03	3.96E-01	2.00E-01
Androstenedione Metabolism	24	1.57	4	6.59E-02	1.00E+00	1.00E+00
Malate-Aspartate Shuttle	10	0.65	2	1.35E-01	1.00E+00	1.00E+00
Aspartate Metabolism	35	2.29	4	1.91E-01	1.00E+00	1.00E+00
Estrone Metabolism	24	1.57	3	2.04E-01	1.00E+00	1.00E+00
Retinol Metabolism	37	2.42	4	2.19E-01	1.00E+00	1.00E+00
Phenylalanine and Tyrosine Metabolism	28	1.83	3	2.75E-01	1.00E+00	1.00E+00
Urea Cycle	29	1.90	3	2.94E-01	1.00E+00	1.00E+00
Citric Acid Cycle	32	2.09	3	3.50E-01	1.00E+00	1.00E+00
Amino Sugar Metabolism	33	2.16	3	3.68E-01	1.00E+00	1.00E+00
Catecholamine Biosynthesis	20	1.31	2	3.81E-01	1.00E+00	1.00E+00
Beta-Alanine Metabolism	34	2.22	3	3.87E-01	1.00E+00	1.00E+00
Homocysteine Degradation	9	0.59	1	4.57E-01	1.00E+00	1.00E+00
Cysteine Metabolism	26	1.70	2	5.17E-01	1.00E+00	1.00E+00
Glycerol Phosphate Shuttle	11	0.72	1	5.27E-01	1.00E+00	1.00E+00
Histidine Metabolism	43	2.81	3	5.44E-01	1.00E+00	1.00E+00
Glycine and Serine Metabolism	59	3.86	4	5.50E-01	1.00E+00	1.00E+00
Taurine and Hypotaurine Metabolism	12	0.79	1	5.58E-01	1.00E+00	1.00E+00
Glucose-Alanine Cycle	13	0.85	1	5.87E-01	1.00E+00	1.00E+00
Ammonia Recycling	32	2.09	2	6.33E-01	1.00E+00	1.00E+00
Glutamate Metabolism	49	3.21	3	6.36E-01	1.00E+00	1.00E+00
Alanine Metabolism	17	1.11	1	6.86E-01	1.00E+00	1.00E+00
Arginine and Proline Metabolism	53	3.47	3	6.90E-01	1.00E+00	1.00E+00
Tyrosine Metabolism	72	4.71	4	7.11E-01	1.00E+00	1.00E+00
Mitochondrial Electron Transport Chain	19	1.24	1	7.27E-01	1.00E+00	1.00E+00
Warburg Effect	58	3.79	3	7.48E-01	1.00E+00	1.00E+00
Glutathione Metabolism	21	1.37	1	7.62E-01	1.00E+00	1.00E+00
Betaine Metabolism	21	1.37	1	7.62E-01	1.00E+00	1.00E+00
Sulfate/Sulfite Metabolism	22	1.44	1	7.78E-01	1.00E+00	1.00E+00
Mitochondrial Beta-Oxidation of Short Chain Saturated Fatty Acids	27	1.77	1	8.43E-01	1.00E+00	1.00E+00
Mitochondrial Beta-Oxidation of Medium Chain Saturated Fatty Acids	27	1.77	1	8.43E-01	1.00E+00	1.00E+00
Arachidonic Acid Metabolism	69	4.51	3	8.47E-01	1.00E+00	1.00E+00
Selenoamino Acid Metabolism	28	1.83	1	8.54E-01	1.00E+00	1.00E+00
Phospholipid Biosynthesis	29	1.90	1	8.63E-01	1.00E+00	1.00E+00
Folate Metabolism	29	1.90	1	8.63E-01	1.00E+00	1.00E+00
Lysine Degradation	30	1.96	1	8.73E-01	1.00E+00	1.00E+00
Purine Metabolism	74	4.84	3	8.80E-01	1.00E+00	1.00E+00
Fatty Acid Elongation In Mitochondria	35	2.29	1	9.10E-01	1.00E+00	1.00E+00
Nicotinate and Nicotinamide Metabolism	37	2.42	1	9.22E-01	1.00E+00	1.00E+00
Porphyrin Metabolism	40	2.62	1	9.37E-01	1.00E+00	1.00E+00
Sphingolipid Metabolism	40	2.62	1	9.37E-01	1.00E+00	1.00E+00
Bile Acid Biosynthesis	65	4.25	2	9.38E-01	1.00E+00	1.00E+00
Propanoate Metabolism	42	2.75	1	9.45E-01	1.00E+00	1.00E+00
Methionine Metabolism	43	2.81	1	9.49E-01	1.00E+00	1.00E+00
Fatty acid Metabolism	43	2.81	1	9.49E-01	1.00E+00	1.00E+00
Pyrimidine Metabolism	59	3.86	1	9.84E-01	1.00E+00	1.00E+00
Valine, Leucine and Isoleucine Degradation	60	3.93	1	9.85E-01	1.00E+00	1.00E+00
Tryptophan Metabolism	60	3.93	1	9.85E-01	1.00E+00	1.00E+00

Table S14. Data related to the over-representation analysis (ORA) of blood plasma metabolites with an abnormal concentration in obese patients (stage 3 obesity in females), presented in Figure S12.

	total	expected	hits	Raw p	Holm p	FDR
Steroidogenesis	43	2.86	17	1.42E-10	1.39E-08	1.39E-08
Androgen and Estrogen Metabolism	33	2.19	10	2.34E-05	2.27E-03	1.15E-03
Androstenedione Metabolism	24	1.59	7	5.85E-04	5.62E-02	1.91E-02
Arginine and Proline Metabolism	53	3.52	6	1.33E-01	1.00E+00	1.00E+00
Malate-Aspartate Shuttle	10	0.66	2	1.39E-01	1.00E+00	1.00E+00
Glycine and Serine Metabolism	59	3.92	6	1.91E-01	1.00E+00	1.00E+00
Aspartate Metabolism	35	2.32	4	1.98E-01	1.00E+00	1.00E+00
Estrone Metabolism	24	1.59	3	2.10E-01	1.00E+00	1.00E+00
Retinol Metabolism	37	2.46	4	2.27E-01	1.00E+00	1.00E+00
Phenylalanine and Tyrosine Metabolism	28	1.86	3	2.83E-01	1.00E+00	1.00E+00
Urea Cycle	29	1.93	3	3.02E-01	1.00E+00	1.00E+00
Ammonia Recycling	32	2.12	3	3.58E-01	1.00E+00	1.00E+00
Mitochondrial Electron Transport Chain	19	1.26	2	3.63E-01	1.00E+00	1.00E+00
Homocysteine Degradation	9	0.60	1	4.63E-01	1.00E+00	1.00E+00
Mitochondrial Beta-Oxidation of Short Chain Saturated Fatty Acids	27	1.79	2	5.46E-01	1.00E+00	1.00E+00
Ketone Body Metabolism	13	0.86	1	5.93E-01	1.00E+00	1.00E+00
Glucose-Alanine Cycle	13	0.86	1	5.93E-01	1.00E+00	1.00E+00
Lysine Degradation	30	1.99	2	6.05E-01	1.00E+00	1.00E+00
Citric Acid Cycle	32	2.12	2	6.41E-01	1.00E+00	1.00E+00
Glutamate Metabolism	49	3.25	3	6.46E-01	1.00E+00	1.00E+00
Beta-Alanine Metabolism	34	2.26	2	6.74E-01	1.00E+00	1.00E+00
Alanine Metabolism	17	1.13	1	6.92E-01	1.00E+00	1.00E+00
Butyrate Metabolism	19	1.26	1	7.32E-01	1.00E+00	1.00E+00
Purine Metabolism	74	4.91	4	7.43E-01	1.00E+00	1.00E+00
Catecholamine Biosynthesis	20	1.33	1	7.50E-01	1.00E+00	1.00E+00
Warburg Effect	58	3.85	3	7.58E-01	1.00E+00	1.00E+00
Glutathione Metabolism	21	1.39	1	7.67E-01	1.00E+00	1.00E+00
Betaine Metabolism	21	1.39	1	7.67E-01	1.00E+00	1.00E+00
Tryptophan Metabolism	60	3.98	3	7.78E-01	1.00E+00	1.00E+00
Sulfate/Sulfite Metabolism	22	1.46	1	7.83E-01	1.00E+00	1.00E+00
Carnitine Synthesis	22	1.46	1	7.83E-01	1.00E+00	1.00E+00
Histidine Metabolism	43	2.86	2	7.95E-01	1.00E+00	1.00E+00
Fatty acid Metabolism	43	2.86	2	7.95E-01	1.00E+00	1.00E+00
Cysteine Metabolism	26	1.73	1	8.36E-01	1.00E+00	1.00E+00
Oxidation of Branched Chain Fatty Acids	26	1.73	1	8.36E-01	1.00E+00	1.00E+00
Phytanic Acid Peroxisomal Oxidation	26	1.73	1	8.36E-01	1.00E+00	1.00E+00
Mitochondrial Beta-Oxidation of Medium Chain Saturated Fatty Acids	27	1.79	1	8.47E-01	1.00E+00	1.00E+00
Folate Metabolism	29	1.93	1	8.68E-01	1.00E+00	1.00E+00
Tyrosine Metabolism	72	4.78	3	8.74E-01	1.00E+00	1.00E+00
Amino Sugar Metabolism	33	2.19	1	9.00E-01	1.00E+00	1.00E+00
Fatty Acid Elongation In Mitochondria	35	2.32	1	9.13E-01	1.00E+00	1.00E+00
Valine, Leucine and Isoleucine Degradation	60	3.98	2	9.21E-01	1.00E+00	1.00E+00
Nicotinate and Nicotinamide Metabolism	37	2.46	1	9.25E-01	1.00E+00	1.00E+00
Porphyrin Metabolism	40	2.66	1	9.39E-01	1.00E+00	1.00E+00
Bile Acid Biosynthesis	65	4.32	2	9.41E-01	1.00E+00	1.00E+00
Propanoate Metabolism	42	2.79	1	9.48E-01	1.00E+00	1.00E+00
Methionine Metabolism	43	2.86	1	9.51E-01	1.00E+00	1.00E+00
Arachidonic Acid Metabolism	69	4.58	1	9.93E-01	1.00E+00	1.00E+00

Table S15. Context support (Z scores calculated by annotation algorithm) for annotating candidate compounds.

m/z	KEGG ID	Z-score	Metabolite name
83.059	C05827	-2.22	Methylimidazole acetaldehyde; 1-Methylimidazole-4-acetaldehyde; Methylimidazoleacetaldehyde
84.044	C01879	-3.08	5-Oxoproline; Pidolic acid; Pyroglutamic acid; 5-Pyrrolidone-2-carboxylic acid; Pyroglutamate; 5-Oxo-L-proline
84.081	C05936	-2.69	N4-Acetylaminobutanal; 4-Acetamidobutanal
84.959	C01563	-2.78	Carbamate; Carbamic acid; Aminoformic acid
84.991	C01353	-2.23	Carbonic acid; Dihydrogen carbonate; H2CO3
85.029	C00086	-4.27	Urea; Carbamide
85.963	C01563	-2.7	Carbamate; Carbamic acid; Aminoformic acid
86.096	C00410	-4.84	Progesterone; 4-Pregnene-3,20-dione
86.956	C01563	-3.58	Carbamate; Carbamic acid; Aminoformic acid
86.963	C01563	-2.62	Carbamate; Carbamic acid; Aminoformic acid
86.993	C01353	-3.35	Carbonic acid; Dihydrogen carbonate; H2CO3
87.994	C05828	-3.54	Methylimidazoleacetic acid; Tele-methylimidazoleacetic acid; 1-Methyl-4-imidazoleacetic acid
88.076	C00555	-2.36	4-Aminobutyraldehyde; 4-Aminobutanal
88.952	C01563	-3.76	Carbamate; Carbamic acid; Aminoformic acid
88.981	C00555	-3.62	4-Aminobutyraldehyde; 4-Aminobutanal
90.976	C00197	-2.36	3-Phospho-D-glycerate; D-Glycerate 3-phosphate; 3-Phospho-(R)-glycerate; 3-Phosphoglycerate
91.054	C00041	-1.91	L-Alanine; L-2-Aminopropionic acid; L-alpha-Alanine
91.054	C05688	-1.94	L-Selenocysteine
91.054	C05688	-2.11	L-Selenocysteine
92.049	C00037	-1.78	Glycine; Aminoacetic acid; Gly
92.949	C01563	-3.72	Carbamate; Carbamic acid; Aminoformic acid
93.012	C15604	-2.02	Prenal
93.045	C00042	-3.28	Succinate; Succinic acid; Butanedionic acid; Ethylenesuccinic acid
93.045	C00256	-1.87	(R)-Lactate; D-Lactate; D-Lactic acid; D-2-Hydroxypropanoic acid; D-2-Hydroxypropionic acid
93.045	C05670	-4.53	3-Aminopropiononitrile; beta-Aminopropionitrile
93.057	C00062	-2.96	L-Arginine; (S)-2-Amino-5-guanidinovaleric acid; L-Arg
96.014	C00546	-3.43	Methylglyoxal; Pyruvaldehyde; Pyruvic aldehyde; 2-Ketopropionaldehyde; 2-Oxopropanal
96.044	C00931	-2.4	Porphobilinogen
96.044	C05665	-1.76	3-Aminopropanal; beta-Aminopropion aldehyde
96.078	C05335	-2.45	L-Selenomethionine
96.958	C00152	-3.25	L-Asparagine; 2-Aminosuccinamic acid
97.028	C00937	-3.35	(R)-Lactaldehyde; D-Lactaldehyde; D-2-Hydroxypropionaldehyde
97.028	C05999	-3.14	Lactaldehyde; 2-Hydroxypropionaldehyde; 2-Hydroxypropanal
97.076	C00986	-3.36	1,3-Diaminopropane; Trimethylenediamine; 1,3-Propanediamine; Propane-1,3-diamine
97.969	C04540	-1.81	N4-(Acetyl-beta-D-glucosaminyl)asparagine; N4-(beta-N-Acetyl-D-glucosaminyl)-L-asparagine
97.991	C00122	-1.77	Fumarate; Fumaric acid; trans-Butenedioic acid
98.949	C00337	-1.75	(S)-Dihydroorotate; (S)-4,5-Dihydroorotate; L-Dihydroorotate; L-Dihydroorotic acid; Dihydro-L-orotic acid
98.975	C00122	-2.29	Fumarate; Fumaric acid; trans-Butenedioic acid
98.995	C00086	-2.67	Urea; Carbamide
99.029	C00037	-3.42	Glycine; Aminoacetic acid; Gly
99.029	C00151	-2.54	L-Amino acid; L-2-Amino acid
99.029	C00405	-2.44	D-Amino acid
99.029	C00937	-2.56	(R)-Lactaldehyde; D-Lactaldehyde; D-2-Hydroxypropionaldehyde
99.029	C05167	-2.58	alpha-Amino acid
99.029	C05999	-2.76	Lactaldehyde; 2-Hydroxypropionaldehyde; 2-Hydroxypropanal
99.043	C00583	-2.5	Propane-1,2-diol; 1,2-Propanediol; Propylene glycol
99.08	C02055	-1.67	N-Acylglycine
99.98	C01563	-4.19	Carbamate; Carbamic acid; Aminoformic acid
99.98	C01563	-4.5	Carbamate; Carbamic acid; Aminoformic acid
100.03	C00037	-3.43	Glycine; Aminoacetic acid; Gly
100.03	C00151	-2.76	L-Amino acid; L-2-Amino acid
100.03	C00405	-2.74	D-Amino acid
100.03	C05167	-2.94	alpha-Amino acid
100.05	C00583	-3.28	Propane-1,2-diol; 1,2-Propanediol; Propylene glycol
100.112	C00410	-6.76	Progesterone; 4-Pregnene-3,20-dione
100.981	C01563	-3.43	Carbamate; Carbamic acid; Aminoformic acid
100.981	C01563	-3.56	Carbamate; Carbamic acid; Aminoformic acid
100.981	C01563	-3.58	Carbamate; Carbamic acid; Aminoformic acid
100.981	C01563	-3.74	Carbamate; Carbamic acid; Aminoformic acid
100.991	C00086	-4.19	Urea; Carbamide
101.007	C01353	-1.68	Carbonic acid; Dihydrogen carbonate; H2CO3
101.048	C00583	-3.55	Propane-1,2-diol; 1,2-Propanediol; Propylene glycol
101.059	C00931	-2.15	Porphobilinogen
101.119	C00410	-6.2	Progesterone; 4-Pregnene-3,20-dione
101.977	C01563	-3.36	Carbamate; Carbamic acid; Aminoformic acid
101.977	C01563	-3.47	Carbamate; Carbamic acid; Aminoformic acid
102.055	C00188	-2.87	L-Threonine; 2-Amino-3-hydroxybutyric acid
102.055	C17234	-2.78	2-Aminobut-2-enoate; (2Z)-2-Aminobut-2-enoic acid; 2-Ammoniobut-2-enoate
102.97	C01563	-3.32	Carbamate; Carbamic acid; Aminoformic acid
103.039	C00109	-2.7	2-Oxobutanoate; 2-Ketobutyric acid; 2-Oxobutyric acid; 2-Oxobutyrate; 2-Oxobutanoic acid
103.039	C00232	-1.74	Succinate semialdehyde; Succinic semialdehyde; 4-Oxobutanoate
103.075	C00931	-1.73	Porphobilinogen
103.084	C00152	-1.91	L-Asparagine; 2-Aminosuccinamic acid
104.03	C05642	-2.23	Formyl-N-acetyl-5-methoxykynurenamine
104.053	C00073	-2.33	L-Methionine; Methionine; L-2-Amino-4methylthiobutyric acid
104.07	C02356	-2.72	(S)-2-Aminobutanoate; (S)-2-Aminobutanoic acid; (S)-2-Aminobutyric acid

104.07	C05698	-3.28	Selenohomocysteine
104.973	C05172	-2.86	Selenophosphoric acid; Selenophosphate
105.017	C00168	-2.44	Hydroxypyruvate; Hydroxypyruvic acid; 3-Hydroxypyruvate; 3-Hydroxypyruvic acid
105.017	C01146	-2.48	2-Hydroxy-3-oxopropanoate; Tartronate semialdehyde
105.034	C02055	-2.17	N-Acylglycine
105.034	C02055	-2.19	N-Acylglycine
106.049	C00065	-2.98	L-Serine; L-2-Amino-3-hydroxypropionic acid; L-3-Hydroxy-alanine; Serine
106.049	C00740	-3.2	D-Serine
108.039	C00258	-3.23	D-Glycerate; Glycerate; (R)-Glycerate; Glyceric acid
108.987	C02166	-1.85	Leukotriene C4; LTC4
110.008	C00022	-2.59	Pyruvate; Pyruvic acid; 2-Oxopropanoate; 2-Oxopropanoic acid; Pyroracemic acid
110.049	C05130	-5.65	Imidazole-4-acetaldehyde; Imidazole acetaldehyde
110.103	C00956	-3.49	L-2-Aminoadipate; L-alpha-Aminoadipate; L-alpha-Aminoadipic acid; L-2-Aminoadipic acid
110.975	C00086	-3.2	Urea; Carbamide
111.01	C00022	-2.25	Pyruvate; Pyruvic acid; 2-Oxopropanoate; 2-Oxopropanoic acid; Pyroracemic acid
111.042	C05130	-5.29	Imidazole-4-acetaldehyde; Imidazole acetaldehyde
111.053	C00956	-2.71	L-2-Aminoadipate; L-alpha-Aminoadipate; L-alpha-Aminoadipic acid; L-2-Aminoadipic acid
111.053	C05130	-3.6	Imidazole-4-acetaldehyde; Imidazole acetaldehyde
111.064	C05141	-3.84	Estriol; 1,3,5(10)-Estratriene-3,16-alpha,17beta-triol
111.079	C00233	-2.22	4-Methyl-2-oxopentanoate; 2-Oxoisocaproate
111.091	C00986	-2.24	1,3-Diaminopropane; Trimethylenediamine; 1,3-Propanediamine; Propane-1,3-diamine
111.978	C05527	-3.18	3-Sulfinylpyruvate; 3-Sulfinopyruvate
112.006	C00022	-2.05	Pyruvate; Pyruvic acid; 2-Oxopropanoate; 2-Oxopropanoic acid; Pyroracemic acid
112.036	C00099	-2.04	beta-Alanine; 3-Aminopropionic acid; 3-Aminopropanoate
112.036	C00213	-2.88	Sarcosine; N-Methylglycine
112.036	C00956	-1.69	L-2-Aminoadipate; L-alpha-Aminoadipate; L-alpha-Aminoadipic acid; L-2-Aminoadipic acid
112.051	C00956	-3.07	L-2-Aminoadipate; L-alpha-Aminoadipate; L-alpha-Aminoadipic acid; L-2-Aminoadipic acid
112.051	C05130	-3.55	Imidazole-4-acetaldehyde; Imidazole acetaldehyde
112.086	C00134	-3.17	Putrescine; 1,4-Butanediamine; 1,4-Diaminobutane; Tetramethylenediamine; Butane-1,4-diamine
112.086	C00388	-3.74	Histamine; 1H-Imidazole-4-ethanamine; 2-(4-Imidazolyl)ethylamine
112.99	C00785	-4.09	Urocanate; Urocanic acid
113.009	C00022	-2.87	Pyruvate; Pyruvic acid; 2-Oxopropanoate; 2-Oxopropanoic acid; Pyroracemic acid
113.009	C00222	-1.72	3-Oxopropanoate; Malonate semialdehyde
113.034	C00099	-1.78	beta-Alanine; 3-Aminopropionic acid; 3-Aminopropanoate
113.034	C00213	-2.27	Sarcosine; N-Methylglycine
113.034	C02055	-1.66	N-Acylglycine
113.038	C00213	-2.49	Sarcosine; N-Methylglycine
113.038	C00213	-2.66	Sarcosine; N-Methylglycine
113.059	C05130	-2.09	Imidazole-4-acetaldehyde; Imidazole acetaldehyde
114.031	C00106	-1.92	Uracil
114.04	C00099	-2.25	beta-Alanine; 3-Aminopropionic acid; 3-Aminopropanoate
114.04	C00213	-2.35	Sarcosine; N-Methylglycine
114.066	C00791	-2.74	Creatinine; 1-Methylglycocyamidine
114.954	C00160	-3.69	Glycolate; Glycolic acid; Hydroxyacetic acid
114.968	C00161	-2.09	2-Oxo acid; 2-Oxocarboxylate; 2-Oxocarboxylic acid; 2-Oxo carboxylate
115.07	C00791	-3.43	Creatinine; 1-Methylglycocyamidine
115.111	C01888	-2.25	Aminoacetone; 1-Amino-2-propanone
115.957	C00785	-3.6	Urocanate; Urocanic acid
115.978	C00160	-3.02	Glycolate; Glycolic acid; Hydroxyacetic acid
115.978	C00160	-3.05	Glycolate; Glycolic acid; Hydroxyacetic acid
115.978	C02929	-4.34	2-Hydroxy carboxylate
115.978	C02929	-4.51	2-Hydroxy carboxylate
115.978	C15565	-2.33	(S)-2-Hydroxyacid; (S)-2-Hydroxy acid; (S)-2-Hydroxyalkanoic acid; (S)-2-Hydroxycarboxylic acid
115.978	C15565	-2.35	(S)-2-Hydroxyacid; (S)-2-Hydroxy acid; (S)-2-Hydroxyalkanoic acid; (S)-2-Hydroxycarboxylic acid
116.034	C00152	-2.94	L-Asparagine; 2-Aminosuccinamic acid
116.048	C00429	-2.03	5,6-Dihydrouracil; Dihydro-2,4(1H,3H)-pyrimidinedione; Dihydrouracile; Dihydrouracil
116.048	C02512	-3.57	3-Cyano-L-alanine; L-3-Cyanoalanine; L-beta-Cyanoalanine
116.96	C00160	-2.93	Glycolate; Glycolic acid; Hydroxyacetic acid
116.975	C00160	-2.77	Glycolate; Glycolic acid; Hydroxyacetic acid
116.975	C02929	-3.47	2-Hydroxy carboxylate
116.975	C15565	-2.71	(S)-2-Hydroxyacid; (S)-2-Hydroxy acid; (S)-2-Hydroxyalkanoic acid; (S)-2-Hydroxycarboxylic acid
116.994	C00433	-3.57	2,5-Dioxopentanoate; 2-Oxoglutarate semialdehyde
117.021	C00122	-2.26	Fumarate; Fumaric acid; trans-Butenedioic acid
117.021	C02119	-1.76	3-Acylpyruvate
117.954	C00108	-1.94	Anthranilate; Anthranilic acid; o-Aminobenzoic acid; Vitamin L1; 2-Aminobenzoate
118.064	C00079	-2.82	L-Phenylalanine; (S)-alpha-Amino-beta-phenylpropionic acid
118.064	C00581	-3.4	Guanidinoacetate; Guanidinoacetic acid; Glycocyamine; N-Amidinoglycine; Guanidoacetic acid
118.086	C05335	-2.32	L-Selenomethionine
118.947	C00160	-3.5	Glycolate; Glycolic acid; Hydroxyacetic acid
118.971	C05823	-2.8	3-Mercaptolactate; L-3-Mercaptolactate; (R)-3-Mercaptolactate
118.991	C01563	-2.89	Carbamate; Carbamic acid; Aminoformic acid
119.017	C00122	-2.2	Fumarate; Fumaric acid; trans-Butenedioic acid
119.036	C00042	-1.9	Succinate; Succinic acid; Butanedionic acid; Ethylenesuccinic acid
119.048	C00166	-4.49	Phenylpyruvate; Phenylpyruvic acid; alpha-Ketohydrocinnamic acid; keto-Phenylpyruvate
119.951	C00160	-4.47	Glycolate; Glycolic acid; Hydroxyacetic acid
119.96	C00168	-2.21	Hydroxypyruvate; Hydroxypyruvic acid; 3-Hydroxypyruvate; 3-Hydroxypyruvic acid
119.975	C00160	-2.46	Glycolate; Glycolic acid; Hydroxyacetic acid
119.987	C01563	-2.92	Carbamate; Carbamic acid; Aminoformic acid
120.003	C01879	-2.58	5-Oxoproline; Pidolic acid; Pyroglutamic acid; 5-Pyrrolidone-2-carboxylic acid; Pyroglutamate; 5-Oxo-L-proline
120.052	C00300	-2.17	Creatine; alpha-Methylguanidino acetic acid; Methylglycocyamine
120.065	C00188	-3.31	L-Threonine; 2-Amino-3-hydroxybutyric acid
120.065	C00581	-3.89	Guanidinoacetate; Guanidinoacetic acid; Glycocyamine; N-Amidinoglycine; Guanidoacetic acid
121.029	C08276	-3.03	3-(Methylthio)propanoate; 3-(Methylthio)propionic acid; 3-Methylthiopropionate; 3-(Methylsulfanyl)propanoate

121.039	C00042	-2.5	Succinate; Succinic acid; Butanedionic acid; Ethylenesuccinic acid
121.966	C04540	-2.2	N4-(Acetyl-beta-D-glucosaminyl)asparagine; N4-(beta-N-Acetyl-D-glucosaminyl)-L-asparagine
121.985	C01563	-2.92	Carbamate; Carbamic acid; Aminoformic acid
122.026	C00097	-1.83	L-Cysteine; L-2-Amino-3-mercaptopropionic acid
122.096	C00410	-6.23	Progesterone; 4-Pregnene-3,20-dione
123.016	C00064	-2.87	L-Glutamine; L-2-Aminoglutaramic acid
123.016	C05823	-1.8	3-Mercaptolactate; L-3-Mercaptolactate; (R)-3-Mercaptolactate
123.024	C00097	-1.7	L-Cysteine; L-2-Amino-3-mercaptopropionic acid
123.024	C08276	-2.17	3-(Methylthio)propanoate; 3-(Methylthio)propionic acid; 3-Methylthiopropionate; 3-(Methylsulfanyl)propanoate
123.043	C00166	-3.99	Phenylpyruvate; Phenylpyruvic acid; alpha-Ketohydrocinnamic acid; keto-Phenylpyruvate
123.082	C05341	-1.84	beta-Alanyl-L-lysine
123.099	C00410	-5.73	Progesterone; 4-Pregnene-3,20-dione
123.927	C00155	-2.23	L-Homocysteine; L-2-Amino-4-mercaptobutyric acid; Homocysteine
124.024	C00097	-1.88	L-Cysteine; L-2-Amino-3-mercaptopropionic acid
124.024	C05726	-1.72	S-Substituted L-cysteine; R-S-Cysteine; L-Cysteine S-conjugate
124.024	C15604	-1.72	Prenal
124.037	C17234	-1.89	2-Aminobut-2-enoate; (2Z)-2-Aminobut-2-enoic acid; 2-Ammoniobut-2-enoate
124.046	C00378	-2.06	Thiamine; Thiamin; Vitamin B1; Aneurin; Antiberiberi factor
124.974	C00547	-2.28	L-Noradrenaline; Noradrenaline; Norepinephrine; Arterenol; 4-[(1R)-2-Amino-1-hydroxyethyl]-1,2-benzenediol
125.02	C00232	-2.02	Succinate semialdehyde; Succinic semialdehyde; 4-Oxobutanoate
125.02	C00232	-2.34	Succinate semialdehyde; Succinic semialdehyde; 4-Oxobutanoate
125.069	C05827	-3.08	Methylimidazole acetaldehyde; 1-Methylimidazole-4-acetaldehyde; Methylimidazoleacetaldehyde
125.986	C01563	-2.8	Carbamate; Carbamic acid; Aminoformic acid
126.036	C00555	-3.26	4-Aminobutyraldehyde; 4-Aminobutanal
126.053	C00334	-2.34	4-Aminobutanoate; 4-Aminobutanoic acid; 4-Aminobutyrate; 4-Aminobutyric acid; gamma-Aminobutyric acid
126.053	C02356	-2.63	(S)-2-Aminobutanoate; (S)-2-Aminobutanoic acid; (S)-2-Aminobutyric acid
126.921	C00547	-1.74	L-Noradrenaline; Noradrenaline; Norepinephrine; Arterenol; 4-[(1R)-2-Amino-1-hydroxyethyl]-1,2-benzenediol
126.968	C05823	-2.66	3-Mercaptolactate; L-3-Mercaptolactate; (R)-3-Mercaptolactate
126.989	C01563	-3.14	Carbamate; Carbamic acid; Aminoformic acid
127.023	C00109	-3.2	2-Oxobutanoate; 2-Ketobutyric acid; 2-Oxobutyric acid; 2-Oxobutyrate; 2-Oxobutanoic acid; alpha-Ketobutyric acid
127.023	C00232	-3.97	Succinate semialdehyde; Succinic semialdehyde; 4-Oxobutanoate
127.023	C15604	-3.16	Prenal
127.072	C05827	-5.26	Methylimidazole acetaldehyde; 1-Methylimidazole-4-acetaldehyde; Methylimidazoleacetaldehyde
127.121	C00881	-2.48	Deoxycytidine; 2'-Deoxycytidine
128.019	C00258	-2.31	D-Glycerate; Glycerate; (R)-Glycerate; Glyceric acid
128.068	C05130	-3.63	Imidazole-4-acetaldehyde; Imidazole acetaldehyde
128.104	C00956	-2.83	L-2-Aminoadipate; L-alpha-Aminoadipate; L-alpha-Aminoadipic acid; L-2-Aminoadipic acid
128.95	C04540	-2.83	N4-(Acetyl-beta-D-glucosaminyl)asparagine; N4-(beta-N-Acetyl-D-glucosaminyl)-L-asparagine
128.962	C05823	-2.63	3-Mercaptolactate; L-3-Mercaptolactate; (R)-3-Mercaptolactate
128.985	C00086	-4.36	Urea; Carbamide
129.02	C00258	-2.51	D-Glycerate; Glycerate; (R)-Glycerate; Glyceric acid
129.088	C05130	-3.29	Imidazole-4-acetaldehyde; Imidazole acetaldehyde
130.016	C00258	-2.84	D-Glycerate; Glycerate; (R)-Glycerate; Glyceric acid
130.049	C01879	-1.69	5-Oxoproline; Pidolic acid; Pyroglutamic acid; 5-Pyrrolidone-2-carboxylic acid; Pyroglutamate; 5-Oxo-L-proline
130.049	C01879	-1.71	5-Oxoproline; Pidolic acid; Pyroglutamic acid; 5-Pyrrolidone-2-carboxylic acid; Pyroglutamate; 5-Oxo-L-proline
130.085	C00062	-2.68	L-Arginine; (S)-2-Amino-5-guanidinovaleric acid; L-Arg
130.085	C05936	-3.66	N4-Acetylaminobutanal; 4-Acetamidobutanal
131.019	C00258	-2.6	D-Glycerate; Glycerate; (R)-Glycerate; Glyceric acid
131.051	C00064	-1.71	L-Glutamine; L-2-Aminoglutaramic acid
131.068	C00956	-1.83	L-2-Aminoadipate; L-alpha-Aminoadipate; L-alpha-Aminoadipic acid; L-2-Aminoadipic acid; L-2-
131.085	C05936	-3.15	N4-Acetylaminobutanal; 4-Acetamidobutanal
131.085	C05936	-3.22	N4-Acetylaminobutanal; 4-Acetamidobutanal
131.962	C00160	-1.87	Glycolate; Glycolic acid; Hydroxyacetic acid
131.973	C00988	-4.35	2-Phosphoglycolate; Phosphoglycolic acid
132.076	C05842	-1.73	N1-Methyl-2-pyridone-5-carboxamide; N'-Methyl-2-pyridone-5-carboxamide; 1-Methyl-5-carboxylamide-2-pyridone
132.101	C00123	-2.01	L-Leucine; 2-Amino-4-methylvaleric acid; (2S)-alpha-2-Amino-4-methylvaleric acid; (2S)-alpha-Leucine
132.972	C01353	-3.45	Carbonic acid; Dihydrogen carbonate; H2CO3
133.016	C00036	-2.1	Oxaloacetate; Oxalacetic acid; Oxaloacetic acid; 2-Oxobutanedioic acid; 2-Oxosuccinic acid; keto-Oxaloacetate
133.032	C00134	-2.05	Putrescine; 1,4-Butanediamine; 1,4-Diaminobutane; Tetramethylenediamine; Butane-1,4-diamine
133.06	C00152	-4.62	L-Asparagine; 2-Aminosuccinamic acid
133.06	C02642	-3.02	3-Ureidopropionate; 3-Ureidopropanoate; beta-Ureidopropionic acid; N-Carbamoyl-beta-alanine
133.085	C06425	-2.07	Icosanoic acid; Eicosanoic acid; Arachidic acid
133.968	C03508	-3.16	L-2-Amino-3-oxobutanoic acid; L-2-Amino-3-oxobutanoate; L-2-Amino-acetoacetate; (S)-2-Amino-3-oxobutanoic acid
134.044	C04377	-1.68	5,10-Methylenetetrahydromethanopterin; N5,N10-Methylenetetrahydromethanopterin
134.063	C02642	-3.57	3-Ureidopropionate; 3-Ureidopropanoate; beta-Ureidopropionic acid; N-Carbamoyl-beta-alanine
134.067	C02642	-1.94	3-Ureidopropionate; 3-Ureidopropanoate; beta-Ureidopropionic acid; N-Carbamoyl-beta-alanine
134.067	C05130	-2.12	Imidazole-4-acetaldehyde; Imidazole acetaldehyde
134.089	C06425	-2.08	Icosanoic acid; Eicosanoic acid; Arachidic acid
135.002	C00117	-2.62	D-Ribose 5-phosphate; Ribose 5-phosphate
135.049	C00049	-2.33	L-Aspartate; L-Aspartic acid; 2-Aminosuccinic acid; L-Asp
135.049	C00402	-2.57	D-Aspartate; D-Aspartic acid
135.08	C00535	-5.68	Testosterone; 17beta-Hydroxy-4-androsten-3-one
135.965	C00433	-2.26	2,5-Dioxopentanoate; 2-Oxoglutarate semialdehyde
135.977	C00062	-3	L-Arginine; (S)-2-Amino-5-guanidinovaleric acid; L-Arg
136.002	C00149	-2.54	(S)-Malate; L-Malate; L-Apple acid; L-Malic acid; L-2-Hydroxybutanedioic acid; Malate; Malic acid
136.047	C00049	-1.98	L-Aspartate; L-Aspartic acid; 2-Aminosuccinic acid; L-Asp
136.047	C00155	-2.98	L-Homocysteine; L-2-Amino-4-mercaptobutyric acid; Homocysteine
136.047	C00791	-3.1	Creatinine; 1-Methylglycocyamidine
136.075	C00166	-3.5	Phenylpyruvate; Phenylpyruvic acid; alpha-Ketohydrocinnamic acid; keto-Phenylpyruvate
137.002	C00433	-2.1	2,5-Dioxopentanoate; 2-Oxoglutarate semialdehyde
137.048	C00155	-1.78	L-Homocysteine; L-2-Amino-4-mercaptobutyric acid; Homocysteine
137.048	C00791	-2.24	Creatinine; 1-Methylglycocyamidine
137.048	C00791	-2.31	Creatinine; 1-Methylglycocyamidine

137.076	C00931	-2.2	Porphobilinogen
137.106	C03406	-2.9	N-(L-Arginino)succinate; 2-(Nomega-L-Arginino)succinate; L-Argininosuccinate; L-Argininosuccinic acid
137.997	C01563	-3.38	Carbamate; Carbamic acid; Aminoformic acid
138.015	C00064	-2.6	L-Glutamine; L-2-Aminoglutaramic acid
138.054	C00148	-3.43	L-Proline; 2-Pyrrolidinecarboxylic acid
138.054	C00791	-3.36	Creatinine; 1-Methylglycocyamidine
138.998	C00122	-4.16	Fumarate; Fumaric acid; trans-Butenedioic acid
138.998	C02119	-2.43	3-Acylpyruvate
139.05	C00148	-2.68	L-Proline; 2-Pyrrolidinecarboxylic acid
139.05	C00785	-2.32	Urocanate; Urocanic acid
139.073	C00379	-1.77	Xylitol
139.962	C04540	-1.79	N4-(Acetyl-beta-D-glucosaminyl)asparagine; N4-(beta-N-Acetyl-D-glucosaminyl)-L-asparagine
140.001	C00122	-1.92	Fumarate; Fumaric acid; trans-Butenedioic acid
140.032	C03508	-2.91	L-2-Amino-3-oxobutanoic acid; L-2-Amino-3-oxobutanoate; L-2-Amino-acetoacetate; (S)-2-Amino-3-oxobutanoic acid
140.067	C05335	-2.55	L-Selenomethionine
140.067	C05828	-3.55	Methylimidazoleacetic acid; Tele-methylimidazoleacetic acid; 1-Methyl-4-imidazoleacetic acid
140.104	C00410	-7.33	Progesterone; 4-Pregnene-3,20-dione
140.903	C00031	-2.86	D-Glucose; Grape sugar; Dextrose; Glucose; D-Glucopyranose
140.959	C04540	-1.78	N4-(Acetyl-beta-D-glucosaminyl)asparagine; N4-(beta-N-Acetyl-D-glucosaminyl)-L-asparagine
140.983	C04540	-2.31	N4-(Acetyl-beta-D-glucosaminyl)asparagine; N4-(beta-N-Acetyl-D-glucosaminyl)-L-asparagine
141.005	C00122	-2	Fumarate; Fumaric acid; trans-Butenedioic acid
141.005	C02119	-1.97	3-Acylpyruvate
141.005	C17234	-4.51	2-Aminobut-2-enoate; (2Z)-2-Aminobut-2-enoic acid; 2-Ammoniobut-2-enoate
141.054	C00785	-3.43	Urocanate; Urocanic acid
141.070	C05335	-2.12	L-Selenomethionine
141.070	C05335	-2.14	L-Selenomethionine
141.070	C05828	-2.56	Methylimidazoleacetic acid; Tele-methylimidazoleacetic acid; 1-Methyl-4-imidazoleacetic acid
141.138	C00642	-2.43	4-Hydroxyphenylacetate; 4-Hydroxyphenylacetic acid
141.958	C00160	-2.16	Glycolate; Glycolic acid; Hydroxyacetic acid
141.983	C00988	-2.2	2-Phosphoglycolate; Phosphoglycolic acid
142.008	C02305	-4.53	Phosphocreatine; N-Phosphocreatine; Creatine phosphate
142.008	C17234	-4.22	2-Aminobut-2-enoate; (2Z)-2-Aminobut-2-enoic acid; 2-Ammoniobut-2-enoate
142.034	C03508	-2.38	L-2-Amino-3-oxobutanoic acid; L-2-Amino-3-oxobutanoate; L-2-Amino-acetoacetate; (S)-2-Amino-3-oxobutanoic acid
142.048	C00295	-1.77	Orotate; Orotic acid; Uracil-6-carboxylic acid
142.097	C00921	-3	Dihydropteroate; 7,8-Dihydropteroate
142.900	C00031	-2.72	D-Glucose; Grape sugar; Dextrose; Glucose; D-Glucopyranose
142.962	C00160	-3.75	Glycolate; Glycolic acid; Hydroxyacetic acid
142.976	C00168	-2.84	Hydroxypyruvate; Hydroxypyruvic acid; 3-Hydroxypyruvate; 3-Hydroxypyruvic acid
142.976	C00957	-2.16	Mercaptopyruvate; 3-Mercaptopyruvic acid; 3-Mercaptopyruvate
142.976	C01146	-2.7	2-Hydroxy-3-oxopropanoate; Tartronate semialdehyde
143.001	C01353	-2.5	Carbonic acid; Dihydrogen carbonate; H2CO3
143.001	C17234	-2.22	2-Aminobut-2-enoate; (2Z)-2-Aminobut-2-enoic acid; 2-Ammoniobut-2-enoate
143.053	C00188	-1.86	L-Threonine; 2-Amino-3-hydroxybutyric acid
143.053	C03824	-3.39	2-Aminomuconate semialdehyde; 2-Aminomuconate 6-semialdehyde
143.068	C00134	-2.58	Putrescine; 1,4-Butanediamine; 1,4-Diaminobutane; Tetramethylenediamine; Butane-1,4-diamine
143.068	C05828	-2.15	Methylimidazoleacetic acid; Tele-methylimidazoleacetic acid; 1-Methyl-4-imidazoleacetic acid
143.996	C01563	-3	Carbamate; Carbamic acid; Aminoformic acid
144.048	C00188	-3.05	L-Threonine; 2-Amino-3-hydroxybutyric acid
144.063	C00188	-2.98	L-Threonine; 2-Amino-3-hydroxybutyric acid
144.943	C00320	-2.39	Thiosulfate; Hyposulfite
144.981	C03508	-2.58	L-2-Amino-3-oxobutanoic acid; L-2-Amino-3-oxobutanoate; L-2-Amino-acetoacetate; (S)-2-Amino-3-oxobutanoic acid
144.992	C00258	-2.34	D-Glycerate; Glycerate; (R)-Glycerate; Glyceric acid
144.992	C05823	-2.62	3-Mercaptolactate; L-3-Mercaptolactate; (R)-3-Mercaptolactate
145.034	C00109	-3.04	2-Oxobutanoate; 2-Ketobutyric acid; 2-Oxobutyric acid; 2-Oxobutyrate; 2-Oxobutanoic acid; alpha-Ketobutyric acid
146.030	C00026	-3.29	2-Oxoglutarate; Oxoglutaric acid; 2-Ketoglutaric acid; alpha-Ketoglutaric acid
146.059	C00581	-2.99	Guanidinoacetate; Guanidinoacetic acid; Glycocyamine; N-Amidinoglycine; Guanidoacetic acid
146.080	C02946	-3.06	4-Acetamidobutanoate; N4-Acetylaminobutanoate
146.080	C04076	-3.16	L-2-Aminoadipate 6-semialdehyde; 2-Aminoadipate 6-semialdehyde; L-Allysine; Allysine
146.090	C01035	-4.86	4-Guanidinobutanoate; 4-Guanidinobutyric acid
146.996	C00086	-2.94	Urea; Carbamide
147.031	C00026	-3.82	2-Oxoglutarate; Oxoglutaric acid; 2-Ketoglutaric acid; alpha-Ketoglutaric acid
147.075	C04076	-2.23	L-2-Aminoadipate 6-semialdehyde; 2-Aminoadipate 6-semialdehyde; L-Allysine; Allysine
147.952	C00160	-2.51	Glycolate; Glycolic acid; Hydroxyacetic acid
147.989	C01563	-3.97	Carbamate; Carbamic acid; Aminoformic acid
148.027	C00258	-2.57	D-Glycerate; Glycerate; (R)-Glycerate; Glyceric acid
148.058	C00025	-3	L-Glutamate; L-Glutamic acid; L-Glutaminic acid; Glutamate
148.058	C00302	-2.86	Glutamate; Glutaminic acid; 2-Aminoglutaric acid
148.078	C00064	-2.21	L-Glutamine; L-2-Aminoglutaramic acid
148.078	C00064	-2.28	L-Glutamine; L-2-Aminoglutaramic acid
148.096	C00152	-2.49	L-Asparagine; 2-Aminosuccinamic acid
148.096	C01035	-2.67	4-Guanidinobutanoate; 4-Guanidinobutyric acid
148.111	C00047	-2.72	L-Lysine; Lysine acid; 2,6-Diaminohexanoic acid
148.935	C00279	-2.21	D-Erythrose 4-phosphate
149.077	C00064	-2.19	L-Glutamine; L-2-Aminoglutaramic acid
150.027	C00612	-1.77	N1-Acetylspermidine
150.058	C00073	-2.23	L-Methionine; Methionine; L-2-Amino-4methylthiobutyric acid
150.058	C05578	-2.73	5,6-Dihydroxyindole; DHI
150.931	C00197	-2.38	3-Phospho-D-glycerate; D-Glycerate 3-phosphate; 3-Phospho-(R)-glycerate; 3-Phosphoglycerate
151.035	C00258	-2.7	D-Glycerate; Glycerate; (R)-Glycerate; Glyceric acid
151.982	C02305	-2.69	Phosphocreatine; N-Phosphocreatine; Creatine phosphate
152.081	C00152	-4.36	L-Asparagine; 2-Aminosuccinamic acid
152.106	C00233	-1.87	4-Methyl-2-oxopentanoate; 2-Oxoisocaproate
152.144	C01051	-2.1	Uroporphyrinogen III

153.016	C00433	-1.78	2,5-Dioxopentanoate; 2-Oxoglutarate semialdehyde
153.016	C00791	-2.04	Creatinine; 1-Methylglycocyamidine
153.032	C00022	-2.6	Pyruvate; Pyruvic acid; 2-Oxopropanoate; 2-Oxopropanoic acid; Pyroracemic acid
153.032	C01879	-3.35	5-Oxoproline; Pidolic acid; Pyroglutamic acid; 5-Pyrrolidone-2-carboxylic acid; Pyroglutamate; 5-Oxo-L-proline
153.032	C01879	-3.4	5-Oxoproline; Pidolic acid; Pyroglutamic acid; 5-Pyrrolidone-2-carboxylic acid; Pyroglutamate; 5-Oxo-L-proline
153.032	C04281	-3.22	L-1-Pyrroline-3-hydroxy-5-carboxylate; 3-Hydroxy-L-1-pyrroline-5-carboxylate
153.032	C04281	-3.42	L-1-Pyrroline-3-hydroxy-5-carboxylate; 3-Hydroxy-L-1-pyrroline-5-carboxylate
153.050	C00233	-1.72	4-Methyl-2-oxopentanoate; 2-Oxoisocaproate
153.050	C04043	-2.06	3,4-Dihydroxyphenylacetaldehyde; Protocatechuatealdehyde
153.099	C02714	-3.04	N-Acetylputrescine
154.033	C01879	-3.96	5-Oxoproline; Pidolic acid; Pyroglutamic acid; 5-Pyrrolidone-2-carboxylic acid; Pyroglutamate; 5-Oxo-L-proline
154.033	C04281	-2.67	L-1-Pyrroline-3-hydroxy-5-carboxylate; 3-Hydroxy-L-1-pyrroline-5-carboxylate
154.058	C00300	-2.24	Creatine; alpha-Methylguanidino acetic acid; Methylglycocyamine
154.058	C00300	-2.26	Creatine; alpha-Methylguanidino acetic acid; Methylglycocyamine
154.058	C05842	-2.21	N1-Methyl-2-pyridone-5-carboxamide; N'-Methyl-2-pyridone-5-carboxamide; 1-Methyl-5-carboxylamide-2-pyridone
154.058	C05843	-2.33	N1-Methyl-4-pyridone-5-carboxamide; N'-Methyl-4-pyridone-5-carboxamide; 1-Methyl-4-pyridone-3-carboximide
154.083	C03758	-1.96	Dopamine; 4-(2-Aminoethyl)-1,2-benzenediol; 4-(2-Aminoethyl)benzene-1,2-diol; 3,4-Dihydroxyphenethylamine
155.013	C00433	-2.45	2,5-Dioxopentanoate; 2-Oxoglutarate semialdehyde
155.013	C00606	-2.29	3-Sulfino-L-alanine; L-Cysteinesulfinic acid; 3-Sulphino-L-alanine; 3-Sulfinoalanine
155.013	C00791	-2.58	Creatinine; 1-Methylglycocyamidine
155.027	C00791	-4.05	Creatinine; 1-Methylglycocyamidine
155.042	C00152	-2.8	L-Asparagine; 2-Aminosuccinamic acid
155.053	C00152	-1.84	L-Asparagine; 2-Aminosuccinamic acid
155.053	C00300	-2.43	Creatine; alpha-Methylguanidino acetic acid; Methylglycocyamine
155.066	C00300	-1.67	Creatine; alpha-Methylguanidino acetic acid; Methylglycocyamine
155.08	C00077	-2.74	L-Ornithine; (S)-2,5-Diaminovaleric acid; (S)-2,5-Diaminopentanoic acid; (S)-2,5-Diaminopentanoate
155.099	C06425	-2.3	Icosanoic acid; Eicosanoic acid; Arachidic acid
155.978	C02119	-1.89	3-Acylpyruvate
156.001	C00433	-2.44	2,5-Dioxopentanoate; 2-Oxoglutarate semialdehyde
156.001	C03508	-3.07	L-2-Amino-3-oxobutanoic acid; L-2-Amino-3-oxobutanoate; L-2-Amino-acetoacetate; (S)-2-Amino-3-oxobutanoic acid
156.076	C00077	-2.32	L-Ornithine; (S)-2,5-Diaminovaleric acid; (S)-2,5-Diaminopentanoic acid; (S)-2,5-Diaminopentanoate
156.076	C00135	-3.01	L-Histidine; (S)-alpha-Amino-1H-imidazole-4-propionic acid
157.014	C00149	-2.34	(S)-Malate; L-Malate; L-Apple acid; L-Malic acid; L-2-Hydroxybutanedioic acid; Malate; Malic acid
157.014	C00581	-3.02	Guanidinoacetate; Guanidinoacetic acid; Glycocyamine; N-Amidinoglycine; Guanidoacetic acid
157.014	C00581	-3.33	Guanidinoacetate; Guanidinoacetic acid; Glycocyamine; N-Amidinoglycine; Guanidoacetic acid
157.014	C03508	-2.96	L-2-Amino-3-oxobutanoic acid; L-2-Amino-3-oxobutanoate; L-2-Amino-acetoacetate; (S)-2-Amino-3-oxobutanoic acid
157.027	C00295	-2.72	Orotate; Orotic acid; Uracil-6-carboxylic acid
157.083	C00077	-2.19	L-Ornithine; (S)-2,5-Diaminovaleric acid; (S)-2,5-Diaminopentanoic acid; (S)-2,5-Diaminopentanoate
157.083	C00135	-2.16	L-Histidine; (S)-alpha-Amino-1H-imidazole-4-propionic acid
157.120	C00931	-2.42	Porphobilinogen
158.021	C00155	-3.85	L-Homocysteine; L-2-Amino-4-mercaptobutyric acid; Homocysteine
158.021	C00188	-4.05	L-Threonine; 2-Amino-3-hydroxybutyric acid
158.021	C00295	-2.16	Orotate; Orotic acid; Uracil-6-carboxylic acid
158.021	C00581	-3.85	Guanidinoacetate; Guanidinoacetic acid; Glycocyamine; N-Amidinoglycine; Guanidoacetic acid
158.021	C00581	-4.03	Guanidinoacetate; Guanidinoacetic acid; Glycocyamine; N-Amidinoglycine; Guanidoacetic acid
158.093	C00062	-2.13	L-Arginine; (S)-2-Amino-5-guanidinovaleric acid; L-Arg
158.996	C00168	-2.7	Hydroxypyruvate; Hydroxypyruvic acid; 3-Hydroxypyruvate; 3-Hydroxypyruvic acid
158.996	C03508	-3.49	L-2-Amino-3-oxobutanoic acid; L-2-Amino-3-oxobutanoate; L-2-Amino-acetoacetate; (S)-2-Amino-3-oxobutanoic acid
159.014	C00149	-2.42	(S)-Malate; L-Malate; L-Apple acid; L-Malic acid; L-2-Hydroxybutanedioic acid; Malate; Malic acid
159.014	C00188	-3.9	L-Threonine; 2-Amino-3-hydroxybutyric acid
159.048	C00134	-2.53	Putrescine; 1,4-Butanediamine; 1,4-Diaminobutane; Tetramethylenediamine; Butane-1,4-diamine
159.048	C00183	-1.9	L-Valine; 2-Amino-3-methylbutyric acid
159.048	C00183	-1.91	L-Valine; 2-Amino-3-methylbutyric acid
159.048	C05335	-2.26	L-Selenomethionine
159.048	C05335	-2.33	L-Selenomethionine
159.075	C00064	-3.55	L-Glutamine; L-2-Aminoglutaramic acid
160.017	C00155	-3.3	L-Homocysteine; L-2-Amino-4-mercaptobutyric acid; Homocysteine
160.017	C00188	-2.87	L-Threonine; 2-Amino-3-hydroxybutyric acid
160.060	C00049	-2.03	L-Aspartate; L-Aspartic acid; 2-Aminosuccinic acid; L-Asp
160.969	C05823	-3.08	3-Mercaptolactate; L-3-Mercaptolactate; (R)-3-Mercaptolactate
160.991	C00108	-1.92	Anthranilate; Anthranilic acid; o-Aminobenzoic acid; Vitamin L1; 2-Aminobenzoate
161.012	C00188	-2.44	L-Threonine; 2-Amino-3-hydroxybutyric acid
161.012	C01353	-1.96	Carbonic acid; Dihydrogen carbonate; H2CO3
161.969	C00169	-3.84	Carbamoyl phosphate
161.969	C05823	-3.27	3-Mercaptolactate; L-3-Mercaptolactate; (R)-3-Mercaptolactate
161.969	C05823	-3.28	3-Mercaptolactate; L-3-Mercaptolactate; (R)-3-Mercaptolactate
162.007	C00258	-2.64	D-Glycerate; Glycerate; (R)-Glycerate; Glyceric acid
162.073	C05130	-2.68	Imidazole-4-acetaldehyde; Imidazole acetaldehyde
163.002	C00258	-2.19	D-Glycerate; Glycerate; (R)-Glycerate; Glyceric acid
163.044	C05828	-3.9	Methylimidazoleacetic acid; Tele-methylimidazoleacetic acid; 1-Methyl-4-imidazoleacetic acid
163.044	C15606	-1.7	1,2-Dihydroxy-5-(methylthio)pent-1-en-3-one; 1,2-Dihydroxy-5-(methylsulfanyl)pent-1-en-3-one
163.073	C01035	-1.76	4-Guanidinobutanoate; 4-Guanidinobutyric acid
163.115	C00109	-3.42	2-Oxobutanoate; 2-Ketobutyric acid; 2-Oxobutyric acid; 2-Oxobutyrate; 2-Oxobutanoic acid; alpha-Ketobutyric acid
163.967	C00169	-3.33	Carbamoyl phosphate
163.967	C05823	-2.36	3-Mercaptolactate; L-3-Mercaptolactate; (R)-3-Mercaptolactate
163.967	C05823	-2.44	3-Mercaptolactate; L-3-Mercaptolactate; (R)-3-Mercaptolactate
164.029	C00295	-3.43	Orotate; Orotic acid; Uracil-6-carboxylic acid
164.029	C05827	-2.41	Methylimidazole acetaldehyde; 1-Methylimidazole-4-acetaldehyde; Methylimidazoleacetaldehyde
164.029	C05827	-2.56	Methylimidazole acetaldehyde; 1-Methylimidazole-4-acetaldehyde; Methylimidazoleacetaldehyde
164.046	C05828	-4.25	Methylimidazoleacetic acid; Tele-methylimidazoleacetic acid; 1-Methyl-4-imidazoleacetic acid
164.046	C15606	-1.69	1,2-Dihydroxy-5-(methylthio)pent-1-en-3-one; 1,2-Dihydroxy-5-(methylsulfanyl)pent-1-en-3-one
165.011	C00279	-3.13	D-Erythrose 4-phosphate
165.053	C00166	-3.18	Phenylpyruvate; Phenylpyruvic acid; alpha-Ketohydrocinnamic acid; keto-Phenylpyruvate

165.053	C02763	-3.38	2-Hydroxy-3-phenylpropenoate; enol-Phenylpyruvate; enol-Phenylpyruvic acid; enol-alpha-Ketohydrocinnamic acid
165.111	C00956	-2.12	L-2-Aminoadipate; L-alpha-Aminoadipate; L-alpha-Aminoadipic acid; L-2-Aminoadipic acid
165.962	C04540	-1.9	N4-(Acetyl-beta-D-glucosaminyl)asparagine; N4-(beta-N-Acetyl-D-glucosaminyl)-L-asparagine
165.965	C00049	-2.46	L-Aspartate; L-Aspartic acid; 2-Aminosuccinic acid; L-Asp
166.993	C05828	-2.03	Methylimidazoleacetic acid; Tele-methylimidazoleacetic acid; 1-Methyl-4-imidazoleacetic acid
167.012	C01563	-2.31	Carbamate; Carbamic acid; Aminoformic acid
167.012	C02835	-2.04	Imidazole-4-acetate; Imidazoleacetic acid; 4-Imidazoleacetate
167.051	C15999	-2.01	L-Methionine (S)-S-oxide
167.066	C05576	-2.6	3,4-Dihydroxyphenylethyleneglycol
168.05	C00025	-4.08	L-Glutamate; L-Glutamic acid; L-Glutaminic acid; Glutamate
168.05	C15999	-4.22	L-Methionine (S)-S-oxide
168.992	C00074	-1.98	Phosphoenolpyruvate; Phosphoenolpyruvic acid; PEP
168.992	C00433	-1.77	2,5-Dioxopentanoate; 2-Oxoglutarate semialdehyde
169.015	C00026	-3.33	2-Oxoglutarate; Oxoglutaric acid; 2-Ketoglutaric acid; alpha-Ketoglutaric acid
169.147	C00315	-1.75	Spermidine; N-(3-Aminopropyl)-1,4-butane-diamine
169.147	C00315	-1.76	Spermidine; N-(3-Aminopropyl)-1,4-butane-diamine
169.998	C00433	-1.69	2,5-Dioxopentanoate; 2-Oxoglutarate semialdehyde
170.012	C00026	-2.56	2-Oxoglutarate; Oxoglutaric acid; 2-Ketoglutaric acid; alpha-Ketoglutaric acid
170.033	C00300	-2.08	Creatine; alpha-Methylguanidino acetic acid; Methylglycocyamine
170.047	C00025	-3.61	L-Glutamate; L-Glutamic acid; L-Glutaminic acid; Glutamate
170.047	C00302	-3.61	Glutamate; Glutaminic acid; 2-Aminoglutaric acid
170.047	C05938	-4.5	L-4-Hydroxyglutamate semialdehyde
170.058	C00064	-2.92	L-Glutamine; L-2-Aminoglutaramic acid
170.058	C00064	-2.99	L-Glutamine; L-2-Aminoglutaramic acid
170.086	C00179	-2.67	Agmatine; (4-Aminobutyl) guanidine
170.086	C00179	-2.83	Agmatine; (4-Aminobutyl) guanidine
170.086	C00547	-2.34	L-Noradrenaline; Noradrenaline; Norepinephrine; Arterenol; 4-[(1R)-2-Amino-1-hydroxyethyl]-1,2-benzenediol
170.927	C03232	-2.74	3-Phosphonooxypyruvate; 3-Phosphonooxypyruvic acid; 3-Phosphohydroxypyruvate
170.993	C00433	-2.01	2,5-Dioxopentanoate; 2-Oxoglutarate semialdehyde
170.993	C00433	-2.02	2,5-Dioxopentanoate; 2-Oxoglutarate semialdehyde
170.993	C01353	-1.81	Carbonic acid; Dihydrogen carbonate; H2CO3
170.993	C02362	-1.68	2-Oxosuccinamate; 2-Oxosuccinamic acid; gamma-Aminooxaloacetate; Oxaloacetamide
171.008	C00062	-2.97	L-Arginine; (S)-2-Amino-5-guanidinovaleric acid; L-Arg
171.008	C01180	-3.41	4-Methylthio-2-oxobutanoic acid; 4-Methylthio-2-oxobutanoate; 4-(Methylsulfanyl)-2-oxobutanoate
171.032	C00300	-2.49	Creatine; alpha-Methylguanidino acetic acid; Methylglycocyamine
171.032	C00300	-2.76	Creatine; alpha-Methylguanidino acetic acid; Methylglycocyamine
171.032	C00300	-2.78	Creatine; alpha-Methylguanidino acetic acid; Methylglycocyamine
171.042	C00025	-3.55	L-Glutamate; L-Glutamic acid; L-Glutaminic acid; Glutamate
171.042	C00025	-3.58	L-Glutamate; L-Glutamic acid; L-Glutaminic acid; Glutamate
171.042	C00302	-3.15	Glutamate; Glutaminic acid; 2-Aminoglutaric acid
171.042	C00302	-3.33	Glutamate; Glutaminic acid; 2-Aminoglutaric acid
171.042	C05938	-2.8	L-4-Hydroxyglutamate semialdehyde
171.042	C05938	-3.24	L-4-Hydroxyglutamate semialdehyde
171.063	C05576	-1.78	3,4-Dihydroxyphenylethyleneglycol
171.075	C00547	-1.89	L-Noradrenaline; Noradrenaline; Norepinephrine; Arterenol; 4-[(1R)-2-Amino-1-hydroxyethyl]-1,2-benzenediol
171.991	C00975	-2.45	Dihydroxyfumarate; Dihydroxyfumaric acid
171.991	C03459	-2.67	2-Hydroxy-3-oxosuccinate; Oxaloglycolate
172.012	C05922	-1.66	Formamidopyrimidine nucleoside triphosphate
172.029	C00300	-2.57	Creatine; alpha-Methylguanidino acetic acid; Methylglycocyamine
172.029	C00300	-2.58	Creatine; alpha-Methylguanidino acetic acid; Methylglycocyamine
172.042	C00025	-2.57	L-Glutamate; L-Glutamic acid; L-Glutaminic acid; Glutamate
173.012	C01180	-3.54	4-Methylthio-2-oxobutanoic acid; 4-Methylthio-2-oxobutanoate; 4-(Methylsulfanyl)-2-oxobutanoate
173.03	C00300	-3.71	Creatine; alpha-Methylguanidino acetic acid; Methylglycocyamine
173.03	C00300	-3.92	Creatine; alpha-Methylguanidino acetic acid; Methylglycocyamine
173.041	C00073	-2.36	L-Methionine; Methionine; L-2-Amino-4methylthiobutyric acid
173.041	C00073	-2.48	L-Methionine; Methionine; L-2-Amino-4methylthiobutyric acid
173.041	C00300	-3.4	Creatine; alpha-Methylguanidino acetic acid; Methylglycocyamine
173.116	C05341	-3.14	beta-Alanyl-L-lysine
174.059	C00123	-2.55	L-Leucine; 2-Amino-4-methylvaleric acid; (2S)-alpha-2-Amino-4-methylvaleric acid; (2S)-alpha-Leucine
174.11	C00931	-2.02	Porphobilinogen
174.127	C00931	-1.84	Porphobilinogen
174.967	C05527	-1.83	3-Sulfinylpyruvate; 3-Sulfinopyruvate
175.057	C00300	-3.36	Creatine; alpha-Methylguanidino acetic acid; Methylglycocyamine
175.117	C00062	-2.87	L-Arginine; (S)-2-Amino-5-guanidinovaleric acid; L-Arg
175.995	C00606	-3.17	3-Sulfino-L-alanine; L-Cysteinesulfinic acid; 3-Sulphino-L-alanine; 3-Sulfinoalanine
176.011	C00108	-1.7	Anthranilate; Anthranilic acid; o-Aminobenzoic acid; Vitamin L1; 2-Aminobenzoate
176.101	C00327	-2.56	L-Citrulline; 2-Amino-5-ureidovaleric acid; Citrulline
176.123	C00062	-2.2	L-Arginine; (S)-2-Amino-5-guanidinovaleric acid; L-Arg
176.974	C05527	-2.13	3-Sulfinylpyruvate; 3-Sulfinopyruvate
177.007	C00606	-1.98	3-Sulfino-L-alanine; L-Cysteinesulfinic acid; 3-Sulphino-L-alanine; 3-Sulfinoalanine
177.007	C00785	-3.05	Urocanate; Urocanic acid
177.007	C03508	-2.8	L-2-Amino-3-oxobutanoic acid; L-2-Amino-3-oxobutanoate; L-2-Amino-acetoacetate; (S)-2-Amino-3-oxobutanoic acid
177.064	C00499	-2.74	Allantoate; Allantoic acid
177.064	C03758	-2.62	Dopamine; 4-(2-Aminoethyl)-1,2-benzenediol; 4-(2-Aminoethyl)benzene-1,2-diol; 3,4-Dihydroxyphenethylamine
177.111	C06425	-1.68	Icosanoic acid; Eicosanoic acid; Arachidic acid
177.979	C05823	-3.34	3-Mercaptolactate; L-3-Mercaptolactate; (R)-3-Mercaptolactate
178.007	C00606	-1.94	3-Sulfino-L-alanine; L-Cysteinesulfinic acid; 3-Sulphino-L-alanine; 3-Sulfinoalanine
178.007	C00785	-2.08	Urocanate; Urocanic acid
178.007	C00785	-2.18	Urocanate; Urocanic acid
178.007	C00785	-2.24	Urocanate; Urocanic acid
178.027	C00155	-3.71	L-Homocysteine; L-2-Amino-4-mercaptobutyric acid; Homocysteine
178.074	C03758	-2.34	Dopamine; 4-(2-Aminoethyl)-1,2-benzenediol; 4-(2-Aminoethyl)benzene-1,2-diol; 3,4-Dihydroxyphenethylamine
178.968	C00988	-3.77	2-Phosphoglycolate; Phosphoglycolic acid

179.002	C00785	-3.98	Urocanate; Urocanic acid
179.002	C00785	-4.31	Urocanate; Urocanic acid
179.002	C03508	-3.05	L-2-Amino-3-oxobutanoic acid; L-2-Amino-3-oxobutanoate; L-2-Amino-acetoacetate; (S)-2-Amino-3-oxobutanoic acid
179.022	C00188	-2.11	L-Threonine; 2-Amino-3-hydroxybutyric acid
179.022	C05828	-4.06	Methylimidazoleacetic acid; Tele-methylimidazoleacetic acid; 1-Methyl-4-imidazoleacetic acid
179.063	C00499	-1.89	Allantoate; Allantoic acid
179.083	C05828	-2.97	Methylimidazoleacetic acid; Tele-methylimidazoleacetic acid; 1-Methyl-4-imidazoleacetic acid
179.105	C05642	-2.73	Formyl-N-acetyl-5-methoxykynurenamine
179.98	C05823	-2.35	3-Mercaptolactate; L-3-Mercaptolactate; (R)-3-Mercaptolactate
180.003	C00295	-3.13	Orotate; Orotic acid; Uracil-6-carboxylic acid
180.003	C00785	-3.69	Urocanate; Urocanic acid
180.003	C00785	-3.73	Urocanate; Urocanic acid
180.003	C03824	-2.27	2-Aminomuconate semialdehyde; 2-Aminomuconate 6-semialdehyde
180.022	C05828	-2.72	Methylimidazoleacetic acid; Tele-methylimidazoleacetic acid; 1-Methyl-4-imidazoleacetic acid
180.022	C05828	-2.78	Methylimidazoleacetic acid; Tele-methylimidazoleacetic acid; 1-Methyl-4-imidazoleacetic acid
180.022	C05828	-2.83	Methylimidazoleacetic acid; Tele-methylimidazoleacetic acid; 1-Methyl-4-imidazoleacetic acid
180.043	C03680	-2.25	4-Imidazolone-5-propanoate; 4-Imidazolone-5-propionic acid; 4,5-Dihydro-4-oxo-5-imidazolepropanoate
180.043	C03680	-2.41	4-Imidazolone-5-propanoate; 4-Imidazolone-5-propionic acid; 4,5-Dihydro-4-oxo-5-imidazolepropanoate
181.011	C00295	-2.13	Orotate; Orotic acid; Uracil-6-carboxylic acid
181.011	C00785	-3.07	Urocanate; Urocanic acid
181.011	C03824	-2.98	2-Aminomuconate semialdehyde; 2-Aminomuconate 6-semialdehyde
181.011	C03824	-3.08	2-Aminomuconate semialdehyde; 2-Aminomuconate 6-semialdehyde
181.029	C05828	-2.62	Methylimidazoleacetic acid; Tele-methylimidazoleacetic acid; 1-Methyl-4-imidazoleacetic acid
181.029	C05828	-2.65	Methylimidazoleacetic acid; Tele-methylimidazoleacetic acid; 1-Methyl-4-imidazoleacetic acid
182.008	C03824	-1.9	2-Aminomuconate semialdehyde; 2-Aminomuconate 6-semialdehyde
182.081	C00082	-3.1	L-Tyrosine; (S)-3-(p-Hydroxyphenyl)alanine; (S)-2-Amino-3-(p-hydroxyphenyl)propionic acid; Tyrosine
182.993	C00160	-2.62	Glycolate; Glycolic acid; Hydroxyacetic acid
183.017	C00025	-2.58	L-Glutamate; L-Glutamic acid; L-Glutaminic acid; Glutamate
183.062	C05582	-2.06	Homovanillate; Homovanillic acid; 3-Methoxy-4-hydroxyphenylacetate
183.062	C05583	-3.27	3-Methoxy-4-hydroxyphenylglycolaldehyde
183.113	C05476	-5.49	Tetrahydrocorticosterone
183.976	C03232	-3.16	3-Phosphonooxypyruvate; 3-Phosphonooxypyruvic acid; 3-Phosphohydroxypyruvate
184.002	C00940	-3.94	2-Oxoglutaramate; 2-Oxoglutaramic acid; alpha-Ketoglutaramate; 2-Ketoglutaramate
184.002	C05572	-3.93	4-Oxoglutaramate; 4-Ketoglutaramate
184.094	C00109	-2.28	2-Oxobutanoate; 2-Ketobutyric acid; 2-Oxobutyric acid; 2-Oxobutyrate; 2-Oxobutanoic acid
185.063	C00152	-2.21	L-Asparagine; 2-Aminosuccinamic acid
185.096	C00788	-2.55	L-Adrenaline; (R)-(-)-Adrenaline; (R)-(-)-Epinephrine; (R)-(-)-Epirenamine; (R)-(-)-Adnephrine
185.096	C00788	-2.74	L-Adrenaline; (R)-(-)-Adrenaline; (R)-(-)-Epinephrine; (R)-(-)-Epirenamine; (R)-(-)-Adnephrine
185.096	C05589	-1.98	L-Normetanephrine
185.096	C05589	-2.23	L-Normetanephrine
185.153	C05665	-2.51	3-Aminopropanal; beta-Aminopropion aldehyde
185.177	C00064	-1.99	L-Glutamine; L-2-Aminoglutaramic acid
185.942	C03232	-3.71	3-Phosphonooxypyruvate; 3-Phosphonooxypyruvic acid; 3-Phosphohydroxypyruvate
186.014	C00025	-1.86	L-Glutamate; L-Glutamic acid; L-Glutaminic acid; Glutamate
186.097	C01035	-4.22	4-Guanidinobutanoate; 4-Guanidinobutyric acid
186.965	C00975	-4.31	Dihydroxyfumarate; Dihydroxyfumaric acid
186.965	C03459	-4.6	2-Hydroxy-3-oxosuccinate; Oxaloglycolate
186.993	C00117	-2.95	D-Ribose 5-phosphate; Ribose 5-phosphate
187.008	C00940	-2.6	2-Oxoglutaramate; 2-Oxoglutaramic acid; alpha-Ketoglutaramate; 2-Ketoglutaramate
187.008	C05572	-2.17	4-Oxoglutaramate; 4-Ketoglutaramate
187.948	C00152	-3.12	L-Asparagine; 2-Aminosuccinamic acid
187.971	C00975	-2.31	Dihydroxyfumarate; Dihydroxyfumaric acid
187.971	C03459	-2.8	2-Hydroxy-3-oxosuccinate; Oxaloglycolate
188.023	C00025	-3.01	L-Glutamate; L-Glutamic acid; L-Glutaminic acid; Glutamate
188.023	C00302	-2.21	Glutamate; Glutaminic acid; 2-Aminoglutaric acid
188.023	C01005	-1.97	O-Phospho-L-serine; L-O-Phosphoserine; 3-Phosphoserine; Dexfosfoserine; 3-Phospho-L-serine
188.023	C02532	-1.98	O-Phospho-D-serine; D-O-Phosphoserine
188.07	C00079	-2.37	L-Phenylalanine; (S)-alpha-Amino-beta-phenylpropionic acid
188.102	C06425	-1.85	Icosanoic acid; Eicosanoic acid; Arachidic acid
188.162	C00956	-1.89	L-2-Aminoadipate; L-alpha-Aminoadipate; L-alpha-Aminoadipic acid; L-2-Aminoadipic acid
188.18	C00612	-2.66	N1-Acetylspermidine
188.967	C00975	-2.3	Dihydroxyfumarate; Dihydroxyfumaric acid
188.967	C00975	-2.43	Dihydroxyfumarate; Dihydroxyfumaric acid
188.967	C03459	-2.3	2-Hydroxy-3-oxosuccinate; Oxaloglycolate
188.967	C03459	-2.48	2-Hydroxy-3-oxosuccinate; Oxaloglycolate
189.074	C00079	-2.85	L-Phenylalanine; (S)-alpha-Amino-beta-phenylpropionic acid
189.110	C00337	-1.88	(S)-Dihydroorotate; (S)-4,5-Dihydroorotate; L-Dihydroorotate; L-Dihydroorotic acid; Dihydro-L-orotic acid
190.052	C01717	-2.77	4-Hydroxy-2-quinolinecarboxylic acid; Kynurenic acid; Kynurenate
190.075	C00079	-1.85	L-Phenylalanine; (S)-alpha-Amino-beta-phenylpropionic acid
190.075	C00624	-2.41	N-Acetyl-L-glutamate; N-Acetyl-L-glutamic acid
190.124	C05711	-1.92	gamma-Glutamyl-beta-cyanoalanine
190.965	C00975	-1.86	Dihydroxyfumarate; Dihydroxyfumaric acid
190.965	C03459	-2.14	2-Hydroxy-3-oxosuccinate; Oxaloglycolate
190.987	C00606	-2.39	3-Sulfino-L-alanine; L-Cysteinesulfinic acid; 3-Sulphino-L-alanine; 3-Sulfinoalanine
191.009	C00062	-2.75	L-Arginine; (S)-2-Amino-5-guanidinovaleric acid; L-Arg
191.04	C01236	-1.91	D-Glucono-1,5-lactone 6-phosphate; 6-Phospho-D-glucono-1,5-lactone
191.072	C00624	-2.45	N-Acetyl-L-glutamate; N-Acetyl-L-glutamic acid
191.072	C00624	-2.79	N-Acetyl-L-glutamate; N-Acetyl-L-glutamic acid
191.104	C00233	-2.26	4-Methyl-2-oxopentanoate; 2-Oxoisocaproate
191.104	C05699	-2.47	L-Selenocystathionine
191.125	C00956	-2.14	L-2-Aminoadipate; L-alpha-Aminoadipate; L-alpha-Aminoadipic acid; L-2-Aminoadipic acid
191.977	C00606	-3.11	3-Sulfino-L-alanine; L-Cysteinesulfinic acid; 3-Sulphino-L-alanine; 3-Sulfinoalanine
192.061	C00547	-2.71	L-Noradrenaline; Noradrenaline; Norepinephrine; Arterenol; 4-[(1R)-2-Amino-1-hydroxyethyl]-1,2-benzenediol

192.977	C00606	-2.48	3-Sulfino-L-alanine; L-Cysteinesulfinic acid; 3-Sulphino-L-alanine; 3-Sulfinoalanine
192.977	C00606	-2.48	3-Sulfino-L-alanine; L-Cysteinesulfinic acid; 3-Sulphino-L-alanine; 3-Sulfinoalanine
192.977	C03232	-1.85	3-Phosphonooxypyruvate; 3-Phosphonooxypyruvic acid; 3-Phosphohydroxypyruvate
193.001	C00086	-5.18	Urea; Carbamide
193.043	C03758	-2.02	Dopamine; 4-(2-Aminoethyl)-1,2-benzenediol; 4-(2-Aminoethyl)benzene-1,2-diol
193.043	C03758	-2.13	Dopamine; 4-(2-Aminoethyl)-1,2-benzenediol; 4-(2-Aminoethyl)benzene-1,2-diol
193.043	C03758	-2.16	Dopamine; 4-(2-Aminoethyl)-1,2-benzenediol; 4-(2-Aminoethyl)benzene-1,2-diol
193.043	C05576	-2.76	3,4-Dihydroxyphenylethyleneglycol
193.158	C12287	-2.32	beta-Ionone; trans-beta-Ionone
194.042	C01693	-3.64	L-Dopachrome; 2-L-Carboxy-2,3-dihydroindole-5,6-quinone
194.042	C03758	-3.04	Dopamine; 4-(2-Aminoethyl)-1,2-benzenediol; 4-(2-Aminoethyl)benzene-1,2-diol
194.042	C03758	-3.19	Dopamine; 4-(2-Aminoethyl)-1,2-benzenediol; 4-(2-Aminoethyl)benzene-1,2-diol
194.042	C03758	-3.32	Dopamine; 4-(2-Aminoethyl)-1,2-benzenediol; 4-(2-Aminoethyl)benzene-1,2-diol
194.042	C03758	-3.4	Dopamine; 4-(2-Aminoethyl)-1,2-benzenediol; 4-(2-Aminoethyl)benzene-1,2-diol
194.042	C04185	-3.49	5,6-Dihydroxyindole-2-carboxylate; DHICA
194.042	C15566	-3.38	D-Dopachrome
194.091	C05298	-1.8	2-Hydroxyestrone
194.925	C03232	-3.1	3-Phosphonooxypyruvate; 3-Phosphonooxypyruvic acid; 3-Phosphohydroxypyruvate
194.962	C00417	-2.58	cis-Aconitate; cis-Aconitic acid
195.018	C03680	-2.6	4-Imidazolone-5-propanoate; 4-Imidazolone-5-propionic acid; 4,5-Dihydro-4-oxo-5-imidazolepropanoate
195.043	C01693	-2.47	L-Dopachrome; 2-L-Carboxy-2,3-dihydroindole-5,6-quinone
195.043	C01693	-2.78	L-Dopachrome; 2-L-Carboxy-2,3-dihydroindole-5,6-quinone
195.043	C03758	-2.95	Dopamine; 4-(2-Aminoethyl)-1,2-benzenediol; 4-(2-Aminoethyl)benzene-1,2-diol
195.043	C03758	-2.96	Dopamine; 4-(2-Aminoethyl)-1,2-benzenediol; 4-(2-Aminoethyl)benzene-1,2-diol
195.043	C04185	-2.73	5,6-Dihydroxyindole-2-carboxylate; DHICA
195.043	C15566	-2.74	D-Dopachrome
195.133	C05642	-3.24	Formyl-N-acetyl-5-methoxykynurenamine
195.967	C00062	-3.66	L-Arginine; (S)-2-Amino-5-guanidinovaleric acid; L-Arg
195.967	C00606	-2.88	3-Sulfino-L-alanine; L-Cysteinesulfinic acid; 3-Sulphino-L-alanine; 3-Sulfinoalanine
196.017	C03680	-4.79	4-Imidazolone-5-propanoate; 4-Imidazolone-5-propionic acid; 4,5-Dihydro-4-oxo-5-imidazolepropanoate
196.017	C03680	-5.01	4-Imidazolone-5-propanoate; 4-Imidazolone-5-propionic acid; 4,5-Dihydro-4-oxo-5-imidazolepropanoate
196.017	C03680	-5.03	4-Imidazolone-5-propanoate; 4-Imidazolone-5-propionic acid; 4,5-Dihydro-4-oxo-5-imidazolepropanoate
196.040	C03758	-2.97	Dopamine; 4-(2-Aminoethyl)-1,2-benzenediol; 4-(2-Aminoethyl)benzene-1,2-diol; 3,4-Dihydroxyphenethylamine
196.916	C00024	-1.92	Acetyl-CoA; Acetyl coenzyme A
196.973	C00417	-2.41	cis-Aconitate; cis-Aconitic acid
196.994	C00337	-2.43	(S)-Dihydroorotate; (S)-4,5-Dihydroorotate; L-Dihydroorotate; L-Dihydroorotic acid; Dihydro-L-orotic acid
197.012	C00026	-3.58	2-Oxoglutarate; Oxoglutaric acid; 2-Ketoglutaric acid; alpha-Ketoglutaric acid
197.012	C03680	-4.21	4-Imidazolone-5-propanoate; 4-Imidazolone-5-propionic acid; 4,5-Dihydro-4-oxo-5-imidazolepropanoate
197.012	C03680	-4.61	4-Imidazolone-5-propanoate; 4-Imidazolone-5-propionic acid; 4,5-Dihydro-4-oxo-5-imidazolepropanoate
197.057	C00439	-1.66	N-Formimino-L-glutamate; N-Formimidoyl-L-glutamate
197.057	C16674	-1.8	Formylisoglutamine; DL-Formylisoglutamine
197.096	C00062	-3.23	L-Arginine; (S)-2-Amino-5-guanidinovaleric acid; L-Arg
197.979	C03406	-1.85	N-(L-Arginino)succinate; 2-(Nomega-L-Arginino)succinate; L-Argininosuccinate; L-Argininosuccinic acid
198.001	C00337	-2.91	(S)-Dihydroorotate; (S)-4,5-Dihydroorotate; L-Dihydroorotate; L-Dihydroorotic acid; Dihydro-L-orotic acid
198.001	C00337	-2.98	(S)-Dihydroorotate; (S)-4,5-Dihydroorotate; L-Dihydroorotate; L-Dihydroorotic acid; Dihydro-L-orotic acid
198.012	C03680	-3.78	4-Imidazolone-5-propanoate; 4-Imidazolone-5-propionic acid; 4,5-Dihydro-4-oxo-5-imidazolepropanoate
198.042	C01042	-1.88	N-Acetyl-L-aspartate; N-Acetyl-L-aspartic acid
198.042	C01045	-3.44	N-Formyl-L-glutamate
198.084	C00327	-2.72	L-Citrulline; 2-Amino-5-ureidovaleric acid; Citrulline
198.127	C00931	-1.74	Porphobilinogen
198.990	C00337	-3.11	(S)-Dihydroorotate; (S)-4,5-Dihydroorotate; L-Dihydroorotate; L-Dihydroorotic acid; Dihydro-L-orotic acid
198.990	C00337	-3.4	(S)-Dihydroorotate; (S)-4,5-Dihydroorotate; L-Dihydroorotate; L-Dihydroorotic acid; Dihydro-L-orotic acid
199.031	C00438	-1.94	N-Carbamoyl-L-aspartate
199.031	C01045	-3.28	N-Formyl-L-glutamate
199.048	C00499	-3.79	Allantoate; Allantoic acid
199.069	C05382	-1.72	Sedoheptulose 7-phosphate; D-Sedoheptulose 7-phosphate; D-altro-Heptulose 7-phosphate
199.145	C05476	-5.09	Tetrahydrocorticosterone
199.170	C05665	-2.11	3-Aminopropanal; beta-Aminopropion aldehyde
199.989	C00337	-2.1	(S)-Dihydroorotate; (S)-4,5-Dihydroorotate; L-Dihydroorotate; L-Dihydroorotic acid; Dihydro-L-orotic acid
200.043	C00499	-3.48	Allantoate; Allantoic acid
200.043	C00499	-3.54	Allantoate; Allantoic acid
200.043	C01042	-2.08	N-Acetyl-L-aspartate; N-Acetyl-L-aspartic acid
200.043	C01045	-1.75	N-Formyl-L-glutamate
200.068	C00109	-2.22	2-Oxobutanoate; 2-Ketobutyric acid; 2-Oxobutyric acid; 2-Oxobutyrate; 2-Oxobutanoic acid; alpha-Ketobutyric acid
201.045	C00499	-2.25	Allantoate; Allantoic acid
201.086	C00750	-2.07	Spermine; N,N'-Bis(3-aminopropyl)-1,4-butanediamine
201.141	C00637	-1.76	Indole-3-acetaldehyde; 2-(Indol-3-yl)acetaldehyde; Indoleacetaldehyde
202.066	C00233	-1.66	4-Methyl-2-oxopentanoate; 2-Oxoisocaproate
202.088	C00078	-1.92	L-Tryptophan; Tryptophan; (S)-alpha-Amino-beta-(3-indolyl)-propionic acid
202.117	C05642	-2.91	Formyl-N-acetyl-5-methoxykynurenamine
203.052	C00031	-2.95	D-Glucose; Grape sugar; Dextrose; Glucose; D-Glucopyranose
203.052	C00095	-4.46	D-Fructose; Levulose; Fruit sugar; D-arabino-Hexulose
203.052	C00124	-2.31	D-Galactose; D-Galactopyranose
203.052	C00137	-1.85	myo-Inositol; D-myo-Inositol; 1D-myo-Inositol; L-myo-Inositol; 1L-myo-Inositol; meso-Inositol; Inositol
203.052	C00159	-3.5	D-Mannose; Mannose; Seminose; Carubinose; D-Mannopyranose
203.052	C00221	-3.14	beta-D-Glucose; beta-D-Glucopyranose
203.052	C00267	-3.24	alpha-D-Glucose
203.052	C00984	-2.01	alpha-D-Galactose
203.052	C01798	-2.73	D-Glucoside
203.052	C02336	-3.51	beta-D-Fructose; beta-Fruit sugar; beta-D-arabino-Hexulose; beta-Levulose; Fructose
203.052	C02478	-3.04	beta-D-Mannoside
204.018	C00166	-2.34	Phenylpyruvate; Phenylpyruvic acid; alpha-Ketohydrocinnamic acid; keto-Phenylpyruvate
204.056	C00031	-2.06	D-Glucose; Grape sugar; Dextrose; Glucose; D-Glucopyranose

204.056	C00095	-3.27	D-Fructose; Levulose; Fruit sugar; D-arabino-Hexulose
204.056	C00159	-2.25	D-Mannose; Mannose; Seminose; Carubinose; D-Mannopyranose
204.056	C00221	-1.84	beta-D-Glucose; beta-D-Glucopyranose
204.056	C00267	-2.05	alpha-D-Glucose
204.056	C01798	-1.75	D-Glucoside
204.056	C02336	-2.38	beta-D-Fructose; beta-Fruit sugar; beta-D-arabino-Hexulose; beta-Levulose; Fructose
204.056	C02478	-2.05	beta-D-Mannoside
205.001	C00785	-2.45	Urocanate; Urocanic acid
205.057	C00031	-2.85	D-Glucose; Grape sugar; Dextrose; Glucose; D-Glucopyranose
205.057	C00095	-4.3	D-Fructose; Levulose; Fruit sugar; D-arabino-Hexulose
205.057	C00124	-1.9	D-Galactose; D-Galactopyranose
205.057	C00137	-2.02	myo-Inositol; D-myo-Inositol; 1D-myo-Inositol; L-myo-Inositol; 1L-myo-Inositol; meso-Inositol; Inositol
205.057	C00159	-3.29	D-Mannose; Mannose; Seminose; Carubinose; D-Mannopyranose
205.057	C00221	-3.18	beta-D-Glucose; beta-D-Glucopyranose
205.057	C00267	-3.23	alpha-D-Glucose
205.057	C00984	-1.75	alpha-D-Galactose
205.057	C01798	-2.94	D-Glucoside
205.057	C02336	-4.01	beta-D-Fructose; beta-Fruit sugar; beta-D-arabino-Hexulose; beta-Levulose; Fructose
205.057	C02478	-3.01	beta-D-Mannoside
205.095	C00078	-2.61	L-Tryptophan; Tryptophan; (S)-alpha-Amino-beta-(3-indolyl)-propionic acid
205.968	C00940	-2.63	2-Oxoglutaramate; 2-Oxoglutaramic acid; alpha-Ketoglutaramate; 2-Ketoglutaramate
206.005	C03508	-2.48	L-2-Amino-3-oxobutanoic acid; L-2-Amino-3-oxobutanoate; L-2-Amino-acetoacetate; (S)-2-Amino-3-oxobutanoic acid
206.033	C00025	-3.38	L-Glutamate; L-Glutamic acid; L-Glutaminic acid; Glutamate
206.068	C01693	-3.69	L-Dopachrome; 2-L-Carboxy-2,3-dihydroindole-5,6-quinone
206.068	C02051	-4.53	Lipoylprotein; H-Protein-lipoyllysine; [GCSH]-N6-lipoyl-L-lysine; [GcvH]-N6-lipoyl-L-lysine
206.068	C15972	-2.2	Enzyme N6-(lipoyl)lysine; Lipoamide-E; [E2 protein]-N6-lipoyl-L-lysine
206.098	C00078	-1.96	L-Tryptophan; Tryptophan; (S)-alpha-Amino-beta-(3-indolyl)-propionic acid
206.098	C00078	-2.07	L-Tryptophan; Tryptophan; (S)-alpha-Amino-beta-(3-indolyl)-propionic acid
206.151	C00612	-1.76	N1-Acetylspermidine
206.19	C05648	-1.69	5-Hydroxy-N-formylkynurenine; 5-Hydroxy-N-formyl-L-kynurenine
207.953	C00160	-3.7	Glycolate; Glycolic acid; Hydroxyacetic acid
207.988	C03406	-2.65	N-(L-Arginino)succinate; 2-(Nomega-L-Arginino)succinate; L-Argininosuccinate; L-Argininosuccinic acid
207.988	C03722	-2.68	Quinolinate; Pyridine-2,3-dicarboxylate; Quinolinic acid; 2,3-Pyridinedicarboxylic acid
207.988	C03722	-2.71	Quinolinate; Pyridine-2,3-dicarboxylate; Quinolinic acid; 2,3-Pyridinedicarboxylic acid
207.988	C03722	-2.88	Quinolinate; Pyridine-2,3-dicarboxylate; Quinolinic acid; 2,3-Pyridinedicarboxylic acid
208.11	C03852	-4.87	Androstan-3alpha,17beta-diol; 5alpha-Androstan-3alpha,17beta-diol
208.818	C00062	-2.65	L-Arginine; (S)-2-Amino-5-guanidinovaleric acid; L-Arg
208.978	C00197	-3.5	3-Phospho-D-glycerate; D-Glycerate 3-phosphate; 3-Phospho-(R)-glycerate; 3-Phosphoglycerate
208.978	C00631	-3.51	2-Phospho-D-glycerate; D-Glycerate 2-phosphate; 2-Phospho-(R)-glycerate
209.033	C15650	-3.64	2,3-Diketo-5-methylthiopentyl-1-phosphate; 5-(Methylthio)-2,3-dioxopentyl phosphate
209.048	C00499	-3.71	Allantoate; Allantoic acid
209.078	C00931	-1.81	Porphobilinogen
209.995	C00062	-3.15	L-Arginine; (S)-2-Amino-5-guanidinovaleric acid; L-Arg
210.05	C00279	-2.7	D-Erythrose 4-phosphate
210.078	C00931	-2.32	Porphobilinogen
210.115	C06425	-2.99	Icosanoic acid; Eicosanoic acid; Arachidic acid
210.973	C03680	-2.51	4-Imidazolone-5-propanoate; 4-Imidazolone-5-propionic acid; 4,5-Dihydro-4-oxo-5-imidazolepropanoate
211.011	C05565	-3.82	Hydantoin-5-propionate; Hydantoin-propionate
211.128	C00735	-4.59	Cortisol; Hydrocortisone; 11beta,17alpha,21-Trihydroxy-4-pregnene-3,20-dione; Kendall's compound F
212.085	C03758	-2.37	Dopamine; 4-(2-Aminoethyl)-1,2-benzenediol; 4-(2-Aminoethyl)benzene-1,2-diol; 3,4-Dihydroxyphenethylamine
212.949	C00975	-4.07	Dihydroxyfumarate; Dihydroxyfumaric acid
212.975	C00417	-3.08	cis-Aconitate; cis-Aconitic acid
212.975	C00785	-4.51	Urocanate; Urocanic acid
213.046	C00624	-2	N-Acetyl-L-glutamate; N-Acetyl-L-glutamic acid
213.077	C06213	-2.35	N-Methyltryptamine; N-Methylindoleethylamine; 1-Methyl-2-(3-indolyl)ethylamine
213.081	C05699	-2.99	L-Selenocystathionine
213.081	C06213	-2.02	N-Methyltryptamine; N-Methylindoleethylamine; 1-Methyl-2-(3-indolyl)ethylamine
213.081	C06213	-2.13	N-Methyltryptamine; N-Methylindoleethylamine; 1-Methyl-2-(3-indolyl)ethylamine
213.975	C00417	-3.17	cis-Aconitate; cis-Aconitic acid
214.073	C00062	-2.33	L-Arginine; (S)-2-Amino-5-guanidinovaleric acid; L-Arg
214.073	C00062	-2.36	L-Arginine; (S)-2-Amino-5-guanidinovaleric acid; L-Arg
214.073	C06751	-2.05	Prenyl-L-cysteine; S-Prenyl-L-cysteine
214.089	C05699	-2.57	L-Selenocystathionine
214.113	C00499	-2.71	Allantoate; Allantoic acid
214.984	C00417	-3.02	cis-Aconitate; cis-Aconitic acid
214.984	C00417	-3.26	cis-Aconitate; cis-Aconitic acid
215.069	C00062	-4.65	L-Arginine; (S)-2-Amino-5-guanidinovaleric acid; L-Arg
215.069	C00062	-4.85	L-Arginine; (S)-2-Amino-5-guanidinovaleric acid; L-Arg
215.099	C02140	-3	Corticosterone; 11beta,21-Dihydroxy-4-pregnene-3,20-dione; Kendall's compound B; Reichstein's substance H
215.205	C01888	-2	Aminoacetone; 1-Amino-2-propanone
215.991	C04409	-2.86	2-Amino-3-carboxymuconate semialdehyde; 2-Amino-3-(3-oxoprop-1-enyl)-but-2-enedioate
216.056	C00327	-2.06	L-Citrulline; 2-Amino-5-ureidovaleric acid; Citrulline
216.084	C00062	-1.74	L-Arginine; (S)-2-Amino-5-guanidinovaleric acid; L-Arg
216.088	C05576	-3.03	3,4-Dihydroxyphenylethyleneglycol
217.002	C01419	-1.87	Cys-Gly; L-Cysteinylglycine
217.002	C05729	-1.73	R-S-Cysteinylglycine
217.955	C00160	-3.31	Glycolate; Glycolic acid; Hydroxyacetic acid
218.009	C01419	-2.58	Cys-Gly; L-Cysteinylglycine
218.009	C01419	-2.74	Cys-Gly; L-Cysteinylglycine
218.009	C05729	-2.45	R-S-Cysteinylglycine
218.009	C05729	-2.48	R-S-Cysteinylglycine
218.919	C00197	-2.21	3-Phospho-D-glycerate; D-Glycerate 3-phosphate; 3-Phospho-(R)-glycerate; 3-Phosphoglycerate
219.098	C00791	-3.76	Creatinine; 1-Methylglycocyamidine

219.957	C00160	-3.21	Glycolate; Glycolic acid; Hydroxyacetic acid
220.029	C00064	-1.68	L-Glutamine; L-2-Aminoglutaramic acid
220.064	C00134	-2.15	Putrescine; 1,4-Butanediamine; 1,4-Diaminobutane; Tetramethylenediamine; Butane-1,4-diamine
220.979	C03372	-1.69	Acylglycerone phosphate; Dihydroxyacetone phosphate acyl ester; 1-Acyl-glycerone 3-phosphate
220.998	C00188	-1.88	L-Threonine; 2-Amino-3-hydroxybutyric acid
221.024	C05583	-2.74	3-Methoxy-4-hydroxyphenylglycolaldehyde
221.173	C00239	-3.15	dCMP; Deoxycytidylic acid; Deoxycytidine monophosphate; Deoxycytidylate; 2'-Deoxycytidine 5'-monophosphate
222.007	C00109	-2	2-Oxobutanoate; 2-Ketobutyric acid; 2-Oxobutyric acid; 2-Oxobutyrate; 2-Oxobutanoic acid; alpha-Ketobutyric acid
222.093	C00931	-2.89	Porphobilinogen
222.941	C03232	-2.31	3-Phosphonooxypyruvate; 3-Phosphonooxypyruvic acid; 3-Phosphohydroxypyruvate
222.967	C03671	-4.14	2-Pyrone-4,6-dicarboxylate
223.012	C03508	-2.45	L-2-Amino-3-oxobutanoic acid; L-2-Amino-3-oxobutanoate; L-2-Amino-acetoacetate; (S)-2-Amino-3-oxobutanoic acid
223.205	C00931	-1.72	Porphobilinogen
223.943	C03232	-2.75	3-Phosphonooxypyruvate; 3-Phosphonooxypyruvic acid; 3-Phosphohydroxypyruvate
223.943	C03232	-2.82	3-Phosphonooxypyruvate; 3-Phosphonooxypyruvic acid; 3-Phosphohydroxypyruvate
223.998	C04409	-2.37	2-Amino-3-carboxymuconate semialdehyde; 2-Amino-3-(3-oxoprop-1-enyl)-but-2-enedioate
224.095	C05576	-2.06	3,4-Dihydroxyphenylethyleneglycol
224.202	C00233	-2.24	4-Methyl-2-oxopentanoate; 2-Oxoisocaproate
225.034	C00279	-1.72	D-Erythrose 4-phosphate
225.965	C00160	-4.17	Glycolate; Glycolic acid; Hydroxyacetic acid
225.998	C04409	-2.29	2-Amino-3-carboxymuconate semialdehyde; 2-Amino-3-(3-oxoprop-1-enyl)-but-2-enedioate
225.998	C04409	-2.32	2-Amino-3-carboxymuconate semialdehyde; 2-Amino-3-(3-oxoprop-1-enyl)-but-2-enedioate
225.998	C04409	-2.56	2-Amino-3-carboxymuconate semialdehyde; 2-Amino-3-(3-oxoprop-1-enyl)-but-2-enedioate
226.042	C00279	-2.26	D-Erythrose 4-phosphate
226.07	C00956	-2.33	L-2-Aminoadipate; L-alpha-Aminoadipate; L-alpha-Aminoadipic acid; L-2-Aminoadipic acid
226.951	C00197	-2.24	3-Phospho-D-glycerate; D-Glycerate 3-phosphate; 3-Phospho-(R)-glycerate; 3-Phosphoglycerate
227.100	C00931	-2.95	Porphobilinogen
227.176	C05298	-3.41	2-Hydroxyestrone
227.234	C00233	-2.14	4-Methyl-2-oxopentanoate; 2-Oxoisocaproate
227.954	C00197	-1.96	3-Phospho-D-glycerate; D-Glycerate 3-phosphate; 3-Phospho-(R)-glycerate; 3-Phosphoglycerate
228.023	C00624	-3.24	N-Acetyl-L-glutamate; N-Acetyl-L-glutamic acid
228.023	C02470	-1.81	Xanthurenic acid; Xanthurenate
228.023	C03287	-3.32	L-Glutamyl 5-phosphate; L-Glutamate 5-phosphate
228.082	C00642	-1.83	4-Hydroxyphenylacetate; 4-Hydroxyphenylacetic acid
228.102	C00499	-2.55	Allantoate; Allantoic acid
228.127	C00612	-1.99	N1-Acetylspermidine
228.173	C05141	-3.51	Estriol; 1,3,5(10)-Estratriene-3,16-alpha,17beta-triol
228.955	C00119	-1.69	5-Phospho-alpha-D-ribose 1-diphosphate; 5-Phosphoribosyl diphosphate; 5-Phosphoribosyl 1-pyrophosphate; PRPP
229.029	C00624	-3.02	N-Acetyl-L-glutamate; N-Acetyl-L-glutamic acid
229.029	C00624	-3.03	N-Acetyl-L-glutamate; N-Acetyl-L-glutamic acid
229.029	C03287	-2.95	L-Glutamyl 5-phosphate; L-Glutamate 5-phosphate
229.98	C05379	-2.13	Oxalosuccinate; Oxalosuccinic acid
230.033	C00624	-2.86	N-Acetyl-L-glutamate; N-Acetyl-L-glutamic acid
230.033	C00624	-3.15	N-Acetyl-L-glutamate; N-Acetyl-L-glutamic acid
230.033	C03287	-3.3	L-Glutamyl 5-phosphate; L-Glutamate 5-phosphate
230.067	C02972	-1.7	Dihydrolipoylprotein; [H protein]-dihydrolipoyllysine; [GCSH]-N6-[(R)-dihydrolipoyl]-L-lysine
230.959	C00279	-2.54	D-Erythrose 4-phosphate
231.17	C05642	-2.57	Formyl-N-acetyl-5-methoxykynurenamine
232.054	C03680	-2.31	4-Imidazolone-5-propanoate; 4-Imidazolone-5-propionic acid; 4,5-Dihydro-4-oxo-5-imidazolepropanoate
232.129	C06425	-2.78	Icosanoic acid; Eicosanoic acid; Arachidic acid
232.927	C03232	-2.45	3-Phosphonooxypyruvate; 3-Phosphonooxypyruvic acid; 3-Phosphohydroxypyruvate
232.97	C00785	-2.16	Urocanate; Urocanic acid
233.151	C00535	-4.56	Testosterone; 17beta-Hydroxy-4-androsten-3-one
234.026	C02305	-2.79	Phosphocreatine; N-Phosphocreatine; Creatine phosphate
234.151	C05141	-4.36	Estriol; 1,3,5(10)-Estratriene-3,16-alpha,17beta-triol
234.181	C00931	-2.22	Porphobilinogen
235.129	C00523	-2.3	Androsterone; 3alpha-Hydroxy-5alpha-androstan-17-one
235.238	C00410	-7.79	Progesterone; 4-Pregnene-3,20-dione
236.041	C00233	-1.89	4-Methyl-2-oxopentanoate; 2-Oxoisocaproate
236.13	C00931	-1.82	Porphobilinogen
236.998	C03406	-2.28	N-(L-Arginino)succinate; 2-(Nomega-L-Arginino)succinate; L-Argininosuccinate; L-Argininosuccinic acid
237.181	C00931	-2.29	Porphobilinogen
238.079	C00931	-2.09	Porphobilinogen
238.148	C01598	-2.71	Melatonin; N-Acetyl-5-methoxytryptamine
238.971	C00160	-1.93	Glycolate; Glycolic acid; Hydroxyacetic acid
238.993	C01061	-1.66	4-Fumarylacetoacetate; 4-Fumarylacetoacetic acid; Fumarylacetoacetate
239.194	C05648	-2.29	5-Hydroxy-N-formylkynurenine; 5-Hydroxy-N-formyl-L-kynurenine
239.968	C00279	-2.48	D-Erythrose 4-phosphate
239.994	C01036	-2.07	4-Maleylacetoacetate; 4-Maleylacetoacetic acid
240.053	C06114	-3.43	gamma-Glutamyl-beta-aminopropiononitrile; gamma-Glutamyl-3-aminopropiononitrile
240.053	C06114	-3.53	gamma-Glutamyl-beta-aminopropiononitrile; gamma-Glutamyl-3-aminopropiononitrile
240.065	C06114	-2.25	gamma-Glutamyl-beta-aminopropiononitrile; gamma-Glutamyl-3-aminopropiononitrile
240.065	C06114	-2.42	gamma-Glutamyl-beta-aminopropiononitrile; gamma-Glutamyl-3-aminopropiononitrile
240.909	C03232	-1.97	3-Phosphonooxypyruvate; 3-Phosphonooxypyruvic acid; 3-Phosphohydroxypyruvate
240.966	C00279	-2.89	D-Erythrose 4-phosphate
241.029	C00049	-2.25	L-Aspartate; L-Aspartic acid; 2-Aminosuccinic acid; L-Asp
241.105	C01598	-2.33	Melatonin; N-Acetyl-5-methoxytryptamine
241.97	C03974	-1.82	2-Acyl-sn-glycerol 3-phosphate
242.138	C05341	-1.81	beta-Alanyl-L-lysine
243.153	C00468	-2.23	Estrone; 3-Hydroxy-1,3,5(10)-estratrien-17-one
243.233	C00064	-1.65	L-Glutamine; L-2-Aminoglutaramic acid
243.973	C00168	-3.09	Hydroxypyruvate; Hydroxypyruvic acid; 3-Hydroxypyruvate; 3-Hydroxypyruvic acid
245.048	C05382	-2.17	Sedoheptulose 7-phosphate; D-Sedoheptulose 7-phosphate; D-altro-Heptulose 7-phosphate

246.024	C02051	-2.13	Lipoylprotein; H-Protein-lipoyllysine; [GCSH]-N6-lipoyl-L-lysine; [GcvH]-N6-lipoyl-L-lysine
246.024	C02051	-2.13	Lipoylprotein; H-Protein-lipoyllysine; [GCSH]-N6-lipoyl-L-lysine; [GcvH]-N6-lipoyl-L-lysine
247.167	C00523	-3.79	Androsterone; 3alpha-Hydroxy-5alpha-androstan-17-one
247.983	C00062	-2.44	L-Arginine; (S)-2-Amino-5-guanidinovaleric acid; L-Arg
248.042	C00931	-2.66	Porphobilinogen
248.054	C06114	-1.92	gamma-Glutamyl-beta-aminopropiononitrile; gamma-Glutamyl-3-aminopropiononitrile
248.067	C00881	-2.47	Deoxycytidine; 2'-Deoxycytidine
249.045	C17755	-2.77	Dopamine quinone
249.918	C00168	-3.48	Hydroxypyruvate; Hydroxypyruvic acid; 3-Hydroxypyruvate; 3-Hydroxypyruvic acid
249.998	C02305	-1.85	Phosphocreatine; N-Phosphocreatine; Creatine phosphate
250.112	C00750	-2.5	Spermine; N,N'-Bis(3-aminopropyl)-1,4-butanediamine
250.146	C01598	-2.54	Melatonin; N-Acetyl-5-methoxytryptamine
250.178	C02166	-2.57	Leukotriene C4; LTC4
250.938	C03232	-2.16	3-Phosphonooxypyruvate; 3-Phosphonooxypyruvic acid; 3-Phosphohydroxypyruvate
250.993	C02305	-3.32	Phosphocreatine; N-Phosphocreatine; Creatine phosphate
251.086	C02055	-1.68	N-Acylglycine
251.125	C01598	-2.51	Melatonin; N-Acetyl-5-methoxytryptamine
251.178	C05692	-1.93	Se-Adenosyl-L-selenohomocysteine; Se-Adenosylselenohomocysteine
251.235	C00931	-2.13	Porphobilinogen
252.125	C00931	-1.78	Porphobilinogen
253.01	C00117	-2.74	D-Ribose 5-phosphate; Ribose 5-phosphate
253.01	C00199	-2.36	D-Ribulose 5-phosphate
253.01	C00231	-1.69	D-Xylulose 5-phosphate
253.01	C00620	-2.67	alpha-D-Ribose 1-phosphate; Ribose 1-phosphate; D-Ribose 1-phosphate
253.053	C00337	-2.21	(S)-Dihydroorotate; (S)-4,5-Dihydroorotate; L-Dihydroorotate; L-Dihydroorotic acid; Dihydro-L-orotic acid
253.178	C00931	-1.9	Porphobilinogen
254.007	C00166	-1.76	Phenylpyruvate; Phenylpyruvic acid; alpha-Ketohydrocinnamic acid; keto-Phenylpyruvate
254.962	C03680	-2.74	4-Imidazolone-5-propanoate; 4-Imidazolone-5-propionic acid; 4,5-Dihydro-4-oxo-5-imidazolepropanoate
254.993	C00122	-1.8	Fumarate; Fumaric acid; trans-Butenedioic acid
255.06	C05576	-2.13	3,4-Dihydroxyphenylethyleneglycol
255.115	C01598	-2.62	Melatonin; N-Acetyl-5-methoxytryptamine
256.021	C00049	-2.2	L-Aspartate; L-Aspartic acid; 2-Aminosuccinic acid; L-Asp
257.055	C00064	-1.94	L-Glutamine; L-2-Aminoglutaramic acid
257.139	C00468	-2.35	Estrone; 3-Hydroxy-1,3,5(10)-estratrien-17-one
257.15	C13713	-2.12	Allotetrahydrodeoxycorticosterone; 5alpha-THDOC; 3alpha,21-Dihydroxy-5alpha-pregnan-20-one
258.001	C01036	-2.04	4-Maleylacetoacetate; 4-Maleylacetoacetic acid
258.149	C00931	-1.92	Porphobilinogen
258.981	C00363	-3.38	dTDP; Deoxythymidine 5'-diphosphate
259.025	C01236	-2.93	D-Glucono-1,5-lactone 6-phosphate; 6-Phospho-D-glucono-1,5-lactone
259.119	C01598	-2.79	Melatonin; N-Acetyl-5-methoxytryptamine
259.165	C05138	-2.71	17alpha-Hydroxypregnenolone
260.05	C00352	-2.77	D-Glucosamine 6-phosphate; D-Glucosamine phosphate
260.094	C01242	-2.67	[Protein]-S8-aminomethyldihydrolipoyllysine; H-protein-S-aminomethyldihydrolipoyllysine
260.949	C00117	-2.49	D-Ribose 5-phosphate; Ribose 5-phosphate
261.01	C00031	-2.16	D-Glucose; Grape sugar; Dextrose; Glucose; D-Glucopyranose
261.046	C00352	-2.87	D-Glucosamine 6-phosphate; D-Glucosamine phosphate
261.046	C00645	-2.31	N-Acetyl-D-mannosamine; 2-Acetamido-2-deoxy-D-mannose
261.046	C01132	-2.28	N-Acetyl-D-galactosamine; N-Acetyl-D-chondrosamine; 2-Acetamido-2-deoxy-D-galactose
261.074	C01239	-1.68	N-Acetyl-beta-D-glucosaminylamine
262.003	C00074	-2.53	Phosphoenolpyruvate; Phosphoenolpyruvic acid; PEP
262.055	C00140	-2.51	N-Acetyl-D-glucosamine; N-Acetylchitosamine; 2-Acetamido-2-deoxy-D-glucose; GlcNAc
262.055	C00140	-2.76	N-Acetyl-D-glucosamine; N-Acetylchitosamine; 2-Acetamido-2-deoxy-D-glucose; GlcNAc
262.055	C00352	-3.22	D-Glucosamine 6-phosphate; D-Glucosamine phosphate
262.055	C00645	-2.88	N-Acetyl-D-mannosamine; 2-Acetamido-2-deoxy-D-mannose
262.055	C00645	-3.1	N-Acetyl-D-mannosamine; 2-Acetamido-2-deoxy-D-mannose
262.055	C01132	-2.84	N-Acetyl-D-galactosamine; N-Acetyl-D-chondrosamine; 2-Acetamido-2-deoxy-D-galactose
262.055	C01132	-3.17	N-Acetyl-D-galactosamine; N-Acetyl-D-chondrosamine; 2-Acetamido-2-deoxy-D-galactose
262.992	C00785	-1.69	Urocanate; Urocanic acid
263.127	C05642	-1.73	Formyl-N-acetyl-5-methoxykynurenamine
263.952	C15650	-2.4	2,3-Diketo-5-methylthiopentyl-1-phosphate; 5-(Methylthio)-2,3-dioxopentyl phosphate
263.993	C03372	-2.04	Acylglycerone phosphate; Dihydroxyacetone phosphate acyl ester; 1-Acyl-glycerone 3-phosphate
264.023	C05648	-1.85	5-Hydroxy-N-formylkynurenine; 5-Hydroxy-N-formyl-L-kynurenine
264.116	C00612	-4.14	N1-Acetylspermidine
264.124	C05648	-1.84	5-Hydroxy-N-formylkynurenine; 5-Hydroxy-N-formyl-L-kynurenine
264.159	C05648	-2.34	5-Hydroxy-N-formylkynurenine; 5-Hydroxy-N-formyl-L-kynurenine
264.238	C00517	-2.16	Hexadecanal; Palmitaldehyde
265.023	C00785	-2.06	Urocanate; Urocanic acid
265.101	C01598	-2.27	Melatonin; N-Acetyl-5-methoxytryptamine
265.165	C06425	-2.49	Icosanoic acid; Eicosanoic acid; Arachidic acid
265.97	C15650	-2.22	2,3-Diketo-5-methylthiopentyl-1-phosphate; 5-(Methylthio)-2,3-dioxopentyl phosphate
266.027	C03508	-2.23	L-2-Amino-3-oxobutanoic acid; L-2-Amino-3-oxobutanoate; L-2-Amino-acetoacetate; (S)-2-Amino-3-oxobutanoic acid
266.062	C05130	-2.35	Imidazole-4-acetaldehyde; Imidazole acetaldehyde
266.077	C05711	-4.37	gamma-Glutamyl-beta-cyanoalanine
266.142	C01598	-3.49	Melatonin; N-Acetyl-5-methoxytryptamine
266.971	C00236	-2.47	3-Phospho-D-glyceroyl phosphate; 1,3-Bisphospho-D-glycerate
266.971	C01159	-3.12	2,3-Bisphospho-D-glycerate; 2,3-Disphospho-D-glycerate; D-Greenwald ester; DPG
267.000	C00166	-3.48	Phenylpyruvate; Phenylpyruvic acid; alpha-Ketohydrocinnamic acid; keto-Phenylpyruvate
268.104	C00330	-2.94	Deoxyguanosine; 2'-Deoxyguanosine
268.184	C00735	-5.8	Cortisol; Hydrocortisone; 11beta,17alpha,21-Trihydroxy-4-pregnene-3,20-dione; Kendall's compound F
268.949	C03508	-1.65	L-2-Amino-3-oxobutanoic acid; L-2-Amino-3-oxobutanoate; L-2-Amino-acetoacetate; (S)-2-Amino-3-oxobutanoic acid
270.046	C00049	-2.04	L-Aspartate; L-Aspartic acid; 2-Aminosuccinic acid; L-Asp
270.187	C00735	-2.85	Cortisol; Hydrocortisone; 11beta,17alpha,21-Trihydroxy-4-pregnene-3,20-dione; Kendall's compound F
270.977	C00117	-3.73	D-Ribose 5-phosphate; Ribose 5-phosphate

270.977	C00199	-4.43	D-Ribulose 5-phosphate
270.977	C00231	-3.76	D-Xylulose 5-phosphate
270.977	C00620	-3.7	alpha-D-Ribose 1-phosphate; Ribose 1-phosphate; D-Ribose 1-phosphate
271.088	C01598	-1.69	Melatonin; N-Acetyl-5-methoxytryptamine
271.151	C00062	-1.95	L-Arginine; (S)-2-Amino-5-guanidinovaleric acid; L-Arg
271.188	C00468	-1.71	Estrone; 3-Hydroxy-1,3,5(10)-estratrien-17-one
272.083	C01598	-1.98	Melatonin; N-Acetyl-5-methoxytryptamine
272.13	C00931	-2.24	Porphobilinogen
272.981	C00468	-2.12	Estrone; 3-Hydroxy-1,3,5(10)-estratrien-17-one
273.074	C05642	-2.51	Formyl-N-acetyl-5-methoxykynurenamine
273.133	C00468	-1.9	Estrone; 3-Hydroxy-1,3,5(10)-estratrien-17-one
273.22	C00523	-2.83	Androsterone; 3alpha-Hydroxy-5alpha-androstan-17-one
273.257	C00535	-5.87	Testosterone; 17beta-Hydroxy-4-androsten-3-one
273.954	C03232	-3.18	3-Phosphonooxypyruvate; 3-Phosphonooxypyruvic acid; 3-Phosphohydroxypyruvate
274.171	C00931	-2.2	Porphobilinogen
274.877	C00197	-1.93	3-Phospho-D-glycerate; D-Glycerate 3-phosphate; 3-Phospho-(R)-glycerate; 3-Phosphoglycerate
275.056	C05141	-2.71	Estriol; 1,3,5(10)-Estratriene-3,16-alpha,17beta-triol
275.084	C05298	-1.73	2-Hydroxyestrone
275.152	C00931	-2	Porphobilinogen
275.236	C00535	-4.41	Testosterone; 17beta-Hydroxy-4-androsten-3-one
276.266	C00062	-2.98	L-Arginine; (S)-2-Amino-5-guanidinovaleric acid; L-Arg
276.919	C00117	-3.23	D-Ribose 5-phosphate; Ribose 5-phosphate
277.175	C05648	-1.97	5-Hydroxy-N-formylkynurenine; 5-Hydroxy-N-formyl-L-kynurenine
279.231	C00249	-2.92	Hexadecanoic acid; Hexadecanoate; Hexadecylic acid; Palmitic acid; Palmitate; Cetylic acid
279.231	C06426	-2.99	(6Z,9Z,12Z)-Octadecatrienoic acid; 6,9,12-Octadecatrienoic acid; gamma-Linolenic acid; Gamolenic acid
279.231	C06427	-3.96	(9Z,12Z,15Z)-Octadecatrienoic acid; alpha-Linolenic acid; 9,12,15-Octadecatrienoic acid; Linolenate; alpha-Linolenate
279.97	C00294	-1.74	Inosine
280.09	C00884	-3.09	Homocarnosine; L-Homocarnosine; gamma-Aminobutyryl histidine; N-(4-Amino-1-oxobutyryl)histidine
280.09	C00884	-3.47	Homocarnosine; L-Homocarnosine; gamma-Aminobutyryl histidine; N-(4-Amino-1-oxobutyryl)histidine
280.09	C01262	-2.2	beta-Alanyl-N(pi)-methyl-L-histidine; Anserine
280.09	C01262	-2.34	beta-Alanyl-N(pi)-methyl-L-histidine; Anserine
280.116	C15977	-1.69	[Dihydrolipoyllysine-residue (2-methylpropanoyl)transferase] S-(2-methylpropanoyl)dihydrolipoyllysine
280.19	C05141	-2.5	Estriol; 1,3,5(10)-Estratriene-3,16-alpha,17beta-triol
280.231	C06427	-1.9	(9Z,12Z,15Z)-Octadecatrienoic acid; alpha-Linolenic acid; 9,12,15-Octadecatrienoic acid; Linolenate; alpha-Linolenate
280.962	C15650	-1.77	2,3-Diketo-5-methylthiopentyl-1-phosphate; 5-(Methylthio)-2,3-dioxopentyl phosphate
280.962	C15651	-1.77	2-Hydroxy-3-keto-5-methylthiopentenyl-1-phosphate; 2-Hydroxy-5-(methylthio)-3-oxopent-1-enyl phosphate
280.971	C00581	-1.81	Guanidinoacetate; Guanidinoacetic acid; Glycocyamine; N-Amidinoglycine; Guanidoacetic acid
281.008	C00160	-2.9	Glycolate; Glycolic acid; Hydroxyacetic acid
281.094	C01262	-1.85	beta-Alanyl-N(pi)-methyl-L-histidine; Anserine
281.094	C01262	-1.96	beta-Alanyl-N(pi)-methyl-L-histidine; Anserine
281.151	C00951	-3.3	Estradiol-17beta; Estradiol; beta-Estradiol
281.206	C05141	-3.14	Estriol; 1,3,5(10)-Estratriene-3,16-alpha,17beta-triol
281.987	C04299	-1.95	D-myo-Inositol 1,2-cyclic phosphate; 1D-myo-Inositol 1,2-cyclic phosphate
282.048	C05711	-3.11	gamma-Glutamyl-beta-cyanoalanine
282.096	C05642	-2.33	Formyl-N-acetyl-5-methoxykynurenamine
282.15	C05141	-3.76	Estriol; 1,3,5(10)-Estratriene-3,16-alpha,17beta-triol
282.218	C05475	-2.47	11beta,21-Dihydroxy-5beta-pregnane-3,20-dione; 5beta-Pregnane-11beta,21-diol-3,20-dione
283.033	C03508	-2.46	L-2-Amino-3-oxobutanoic acid; L-2-Amino-3-oxobutanoate; L-2-Amino-acetoacetate; (S)-2-Amino-3-oxobutanoic acid
283.117	C00062	-2.82	L-Arginine; (S)-2-Amino-5-guanidinovaleric acid; L-Arg
283.145	C05761	-1.67	Tetradecanoyl-[acp]; Tetradecanoyl-[acyl-carrier protein]; Myristoyl-[acyl-carrier protein]
283.171	C02140	-4.98	Corticosterone; 11beta,21-Dihydroxy-4-pregnene-3,20-dione; Kendall's compound B; Reichstein's substance H
283.204	C05141	-2.88	Estriol; 1,3,5(10)-Estratriene-3,16-alpha,17beta-triol
283.222	C00468	-3	Estrone; 3-Hydroxy-1,3,5(10)-estratrien-17-one
283.957	C15650	-2.26	2,3-Diketo-5-methylthiopentyl-1-phosphate; 5-(Methylthio)-2,3-dioxopentyl phosphate
283.957	C15651	-2.2	2-Hydroxy-3-keto-5-methylthiopentenyl-1-phosphate; 2-Hydroxy-5-(methylthio)-3-oxopent-1-enyl phosphate
283.977	C00279	-2.03	D-Erythrose 4-phosphate
284.206	C05141	-4.2	Estriol; 1,3,5(10)-Estratriene-3,16-alpha,17beta-triol
284.909	C00279	-3.25	D-Erythrose 4-phosphate
285.22	C00535	-2.85	Testosterone; 17beta-Hydroxy-4-androsten-3-one
285.241	C00378	-2.08	Thiamine; Thiamin; Vitamin B1; Aneurin; Antiberiberi factor
285.296	C06425	-1.87	Icosanoic acid; Eicosanoic acid; Arachidic acid
286.07	C01762	-2.12	Xanthosine
286.106	C05340	-2.08	beta-Alanyl-L-arginine
286.222	C00535	-2.56	Testosterone; 17beta-Hydroxy-4-androsten-3-one
286.25	C05139	-4.75	16alpha-Hydroxydehydroepiandrosterone; 5-Androstene-3beta,16alpha-diol-17-one
287.176	C00931	-2.19	Porphobilinogen
287.196	C00280	-3.52	Androstenedione; Androst-4-ene-3,17-dione; 4-Androstene-3,17-dione
287.236	C00535	-4.6	Testosterone; 17beta-Hydroxy-4-androsten-3-one
288.104	C00378	-1.77	Thiamine; Thiamin; Vitamin B1; Aneurin; Antiberiberi factor
288.104	C05642	-1.92	Formyl-N-acetyl-5-methoxykynurenamine
288.177	C05642	-2.65	Formyl-N-acetyl-5-methoxykynurenamine
288.239	C00535	-3.27	Testosterone; 17beta-Hydroxy-4-androsten-3-one
289.1	C05642	-2.11	Formyl-N-acetyl-5-methoxykynurenamine
289.174	C00523	-4.47	Androsterone; 3alpha-Hydroxy-5alpha-androstan-17-one
289.214	C00535	-3.23	Testosterone; 17beta-Hydroxy-4-androsten-3-one
289.214	C00674	-4.01	5alpha-Androstane-3,17-dione; Androstanedione
289.214	C01227	-3.38	Dehydroepiandrosterone; 3beta-Hydroxyandrost-5-en-17-one; Dehydroisoandrosterone; DHA; DHEA
289.214	C03772	-4.06	5beta-Androstane-3,17-dione
289.251	C00535	-3.94	Testosterone; 17beta-Hydroxy-4-androsten-3-one
290.213	C00735	-4.43	Cortisol; Hydrocortisone; 11beta,17alpha,21-Trihydroxy-4-pregnene-3,20-dione; Kendall's compound F
290.255	C00535	-4.07	Testosterone; 17beta-Hydroxy-4-androsten-3-one
290.886	C00168	-1.69	Hydroxypyruvate; Hydroxypyruvic acid; 3-Hydroxypyruvate; 3-Hydroxypyruvic acid
291.013	C03508	-2.25	L-2-Amino-3-oxobutanoic acid; L-2-Amino-3-oxobutanoate; L-2-Amino-acetoacetate; (S)-2-Amino-3-oxobutanoic acid

291.084	C00330	-1.7	Deoxyguanosine; 2'-Deoxyguanosine
291.14	C00108	-2.19	Anthranilate; Anthranilic acid; o-Aminobenzoic acid; Vitamin L1; 2-Aminobenzoate
291.192	C02165	-2.67	Leukotriene B4; (6Z,8E,10E,14Z)-(5S,12R)-5,12-Dihydroxyeicosa-6,8,10,14-tetraenoate
291.23	C00523	-4.12	Androsterone; 3alpha-Hydroxy-5alpha-androstan-17-one
291.23	C03917	-4.59	Dihydrotestosterone; 17beta-Hydroxyandrostan-3-one; Androstanolone; 17beta-Hydroxy-5alpha-androstan-3-one
291.23	C04295	-4	Androstenediol; Androst-5-ene-3beta,17beta-diol; 3beta,17beta-Dihydroxyandrost-5-ene
291.23	C04373	-3.34	Etiocholanolone; 3alpha-Hydroxy-5beta-androstan-17-one; Etiocholan-3alpha-ol-17-one
291.23	C05293	-4.11	5beta-Dihydrotestosterone
291.267	C00535	-4.18	Testosterone; 17beta-Hydroxy-4-androsten-3-one
292.014	C00640	-2.44	(3S)-3-Hydroxyacyl-CoA; (S)-3-Hydroxyacyl-CoA
292.042	C05512	-2.76	Deoxyinosine
292.051	C05382	-1.84	Sedoheptulose 7-phosphate; D-Sedoheptulose 7-phosphate; D-altro-Heptulose 7-phosphate
292.051	C05512	-2.46	Deoxyinosine
292.051	C05512	-2.49	Deoxyinosine
292.051	C05512	-2.62	Deoxyinosine
292.107	C06114	-2.12	gamma-Glutamyl-beta-aminopropiononitrile; gamma-Glutamyl-3-aminopropiononitrile
292.197	C00909	-2.09	Leukotriene A4; LTA4; (7E,9E,11Z,14Z)-(5S,6S)-5,6-Epoxyeicosa-7,9,11,14-tetraenoic acid
292.226	C04707	-4.08	(5Z,13E)-11alpha-Hydroxy-9,15-dioxoprost-13-enoate; (5Z)-(15S)-11alpha-Hydroxy-9,15-dioxoprosta-13-enoate
292.884	C00168	-2.09	Hydroxypyruvate; Hydroxypyruvic acid; 3-Hydroxypyruvate; 3-Hydroxypyruvic acid
293.012	C00279	-2.51	D-Erythrose 4-phosphate
293.068	C02763	-3	2-Hydroxy-3-phenylpropenoate; enol-Phenylpyruvate; enol-Phenylpyruvic acid; enol-alpha-Ketohydrocinnamic acid
293.097	C03406	-2.18	N-(L-Arginino)succinate; 2-(Nomega-L-Arginino)succinate; L-Argininosuccinate; L-Argininosuccinic acid
293.176	C05141	-4.59	Estriol; 1,3,5(10)-Estratriene-3,16-alpha,17beta-triol
293.244	C03852	-2.67	Androstan-3alpha,17beta-diol; 5alpha-Androstan-3alpha,17beta-diol
294.07	C00559	-2.11	Deoxyadenosine; 2'-Deoxyadenosine
294.101	C00655	-2.02	Xanthosine 5'-phosphate; Xanthylic acid; XMP; (9-D-Ribosylxanthine)-5'-phosphate
294.185	C05141	-3.88	Estriol; 1,3,5(10)-Estratriene-3,16-alpha,17beta-triol
294.213	C00249	-1.87	Hexadecanoic acid; Hexadecanoate; Hexadecylic acid; Palmitic acid; Palmitate; Cetylic acid
295.127	C05141	-4.66	Estriol; 1,3,5(10)-Estratriene-3,16-alpha,17beta-triol
295.165	C00951	-4.05	Estradiol-17beta; Estradiol; beta-Estradiol
295.259	C05699	-1.8	L-Selenocystathionine
296.184	C06425	-1.79	Icosanoic acid; Eicosanoic acid; Arachidic acid
296.257	C06427	-2.81	(9Z,12Z,15Z)-Octadecatrienoic acid; alpha-Linolenic acid; 9,12,15-Octadecatrienoic acid; Linolenate; alpha-Linolenate
296.919	C03508	-1.85	L-2-Amino-3-oxobutanoic acid; L-2-Amino-3-oxobutanoate; L-2-Amino-acetoacetate; (S)-2-Amino-3-oxobutanoic acid
297.12	C04688	-1.65	(3R)-3-Hydroxytetradecanoyl-[acyl-carrier protein]; (R)-3-Hydroxytetradecanoyl-[acyl-carrier protein]
297.179	C00535	-3.71	Testosterone; 17beta-Hydroxy-4-androsten-3-one
297.219	C05141	-2.7	Estriol; 1,3,5(10)-Estratriene-3,16-alpha,17beta-triol
297.239	C05648	-2.07	5-Hydroxy-N-formylkynurenine; 5-Hydroxy-N-formyl-L-kynurenine
297.314	C06425	-2.55	Icosanoic acid; Eicosanoic acid; Arachidic acid
298.274	C06425	-1.85	Icosanoic acid; Eicosanoic acid; Arachidic acid
299.083	C00523	-2.49	Androsterone; 3alpha-Hydroxy-5alpha-androstan-17-one
300.004	C05827	-2.24	Methylimidazole acetaldehyde; 1-Methylimidazole-4-acetaldehyde; Methylimidazoleacetaldehyde
300.066	C05642	-3.59	Formyl-N-acetyl-5-methoxykynurenamine
300.135	C00468	-1.96	Estrone; 3-Hydroxy-1,3,5(10)-estratrien-17-one
300.199	C05141	-2.96	Estriol; 1,3,5(10)-Estratriene-3,16-alpha,17beta-triol
300.289	C00319	-1.99	Sphingosine; Sphingenine; Sphingoid; Sphing-4-enine
300.985	C00279	-1.97	D-Erythrose 4-phosphate
302.214	C02140	-8.65	Corticosterone; 11beta,21-Dihydroxy-4-pregnene-3,20-dione; Kendall's compound B; Reichstein's substance H
303.03	C05332	-2.14	Phenethylamine; 2-Phenylethylamine; beta-Phenylethylamine; Phenylethylamine
303.058	C00357	-2	N-Acetyl-D-glucosamine 6-phosphate
303.058	C04257	-2.11	N-Acetyl-D-mannosamine 6-phosphate; N-Acetylmannosamine 6-phosphate
303.081	C05642	-3.18	Formyl-N-acetyl-5-methoxykynurenamine
303.176	C16677	-2.34	all-trans-4-Hydroxyretinoic acid
303.301	C00836	-3.41	Sphinganine; Dihydrosphingosine; 2-Amino-1,3-dihydroxyoctadecane
305.11	C00064	-1.95	L-Glutamine; L-2-Aminoglutaramic acid
305.209	C05139	-3.31	16alpha-Hydroxydehydroepiandrosterone; 5-Androstene-3beta,16alpha-diol-17-one
305.209	C05291	-3.36	7alpha-Hydroxytestosterone
305.209	C05294	-2.98	19-Hydroxytestosterone; 17beta,19-Dihydroxyandrost-4-en-3-one
306.068	C00130	-2.52	IMP; Inosinic acid; Inosine monophosphate; Inosine 5'-monophosphate; Inosine 5'-phosphate; 5'-Inosinate
306.128	C00097	-2.68	L-Cysteine; L-2-Amino-3-mercaptopropionic acid
307.064	C01762	-2.45	Xanthosine
307.113	C00931	-2.72	Porphobilinogen
307.173	C06425	-2.66	Icosanoic acid; Eicosanoic acid; Arachidic acid
307.201	C00951	-3.06	Estradiol-17beta; Estradiol; beta-Estradiol
307.279	C04707	-3.61	(5Z,13E)-11alpha-Hydroxy-9,15-dioxoprost-13-enoate; (5Z)-(15S)-11alpha-Hydroxy-9,15-dioxoprosta-13-enoate
308.061	C00239	-2.26	dCMP; Deoxycytidylic acid; Deoxycytidine monophosphate; Deoxycytidylate; 2'-Deoxycytidine 5'-monophosphate
308.061	C01762	-2.87	Xanthosine
308.089	C00051	-1.74	Glutathione; 5-L-Glutamyl-L-cysteinylglycine; N-(N-gamma-L-Glutamyl-L-cysteinyl)glycine
308.089	C02320	-1.65	R-S-Glutathione
308.147	C13713	-5.31	Allotetrahydrodeoxycorticosterone; 5alpha-THDOC; 3alpha,21-Dihydroxy-5alpha-pregnan-20-one
308.203	C00376	-1.86	Retinal; Vitamin A aldehyde; Retinene; all-trans-Retinal; all-trans-Vitamin A aldehyde; all-trans-Retinene
308.221	C00836	-1.99	Sphinganine; Dihydrosphingosine; 2-Amino-1,3-dihydroxyoctadecane
308.954	C00279	-2.95	D-Erythrose 4-phosphate
309.051	C00365	-3.24	dUMP; Deoxyuridylic acid; Deoxyuridine monophosphate; Deoxyuridine 5'-phosphate
309.13	C05648	-1.68	5-Hydroxy-N-formylkynurenine; 5-Hydroxy-N-formyl-L-kynurenine
309.217	C00410	-4.86	Progesterone; 4-Pregnene-3,20-dione
310.166	C05648	-1.91	5-Hydroxy-N-formylkynurenine; 5-Hydroxy-N-formyl-L-kynurenine
310.207	C00410	-3.37	Progesterone; 4-Pregnene-3,20-dione
310.222	C00410	-3.46	Progesterone; 4-Pregnene-3,20-dione
311.126	C00468	-1.73	Estrone; 3-Hydroxy-1,3,5(10)-estratrien-17-one
311.126	C00468	-1.75	Estrone; 3-Hydroxy-1,3,5(10)-estratrien-17-one
311.126	C00468	-1.84	Estrone; 3-Hydroxy-1,3,5(10)-estratrien-17-one
311.161	C02140	-4.52	Corticosterone; 11beta,21-Dihydroxy-4-pregnene-3,20-dione; Kendall's compound B; Reichstein's substance H

311.161	C05141	-3.57	Estriol; 1,3,5(10)-Estratriene-3,16-alpha,17beta-triol
311.161	C05301	-3.43	2-Hydroxyestradiol; 2-Hydroxyestradiol-17beta; 2-OH-Estradiol
311.195	C00674	-2.13	5alpha-Androstane-3,17-dione; Androstanedione
311.195	C00674	-2.39	5alpha-Androstane-3,17-dione; Androstanedione
311.195	C01227	-1.9	Dehydroepiandrosterone; 3beta-Hydroxyandrost-5-en-17-one; Dehydroisoandrosterone; DHA; DHEA
311.195	C03772	-2.3	5beta-Androstane-3,17-dione
311.233	C00951	-2.59	Estradiol-17beta; Estradiol; beta-Estradiol
312.161	C13713	-2.85	Allotetrahydrodeoxycorticosterone; 5alpha-THDOC; 3alpha,21-Dihydroxy-5alpha-pregnan-20-one
312.197	C00535	-2.03	Testosterone; 17beta-Hydroxy-4-androsten-3-one
312.197	C00674	-2.27	5alpha-Androstane-3,17-dione; Androstanedione
312.197	C01227	-1.89	Dehydroepiandrosterone; 3beta-Hydroxyandrost-5-en-17-one; Dehydroisoandrosterone; DHA; DHEA
312.197	C03772	-2.53	5beta-Androstane-3,17-dione
312.238	C00535	-2.36	Testosterone; 17beta-Hydroxy-4-androsten-3-one
312.889	C04540	-2.74	N4-(Acetyl-beta-D-glucosaminyl)asparagine; N4-(beta-N-Acetyl-D-glucosaminyl)-L-asparagine
313.114	C03406	-1.88	N-(L-Arginino)succinate; 2-(Nomega-L-Arginino)succinate; L-Argininosuccinate; L-Argininosuccinic acid
313.141	C02140	-3.14	Corticosterone; 11beta,21-Dihydroxy-4-pregnene-3,20-dione; Kendall's compound B; Reichstein's substance H
313.176	C05141	-1.95	Estriol; 1,3,5(10)-Estratriene-3,16-alpha,17beta-triol
313.209	C00523	-2.56	Androsterone; 3alpha-Hydroxy-5alpha-androstan-17-one
313.209	C03917	-2.23	Dihydrotestosterone; 17beta-Hydroxyandrostan-3-one; Androstanolone; 17beta-Hydroxy-5alpha-androstan-3-one
313.209	C04295	-2.09	Androstenediol; Androst-5-ene-3beta,17beta-diol; 3beta,17beta-Dihydroxyandrost-5-ene; 3beta
313.209	C04373	-2.68	Etiocholanolone; 3alpha-Hydroxy-5beta-androstan-17-one; Etiocholan-3alpha-ol-17-one
313.209	C05293	-1.96	5beta-Dihydrotestosterone
313.31	C06425	-2.62	Icosanoic acid; Eicosanoic acid; Arachidic acid
313.31	C06425	-2.65	Icosanoic acid; Eicosanoic acid; Arachidic acid
314.126	C01301	-3.13	3alpha,7alpha,12alpha-Trihydroxy-5beta-cholestan-26-al
314.178	C00735	-2.2	Cortisol; Hydrocortisone; 11beta,17alpha,21-Trihydroxy-4-pregnene-3,20-dione; Kendall's compound F
314.212	C00523	-2.23	Androsterone; 3alpha-Hydroxy-5alpha-androstan-17-one
314.312	C06425	-1.99	Icosanoic acid; Eicosanoic acid; Arachidic acid
314.312	C06425	-2.04	Icosanoic acid; Eicosanoic acid; Arachidic acid
314.887	C04540	-2.87	N4-(Acetyl-beta-D-glucosaminyl)asparagine; N4-(beta-N-Acetyl-D-glucosaminyl)-L-asparagine
315.079	C00049	-2.35	L-Aspartate; L-Aspartic acid; 2-Aminosuccinic acid; L-Asp
315.188	C05139	-2.57	16alpha-Hydroxydehydroepiandrosterone; 5-Androstene-3beta,16alpha-diol-17-one
315.229	C00410	-5.02	Progesterone; 4-Pregnene-3,20-dione
315.229	C03852	-4.3	Androstan-3alpha,17beta-diol; 5alpha-Androstan-3alpha,17beta-diol
316.196	C02140	-2.01	Corticosterone; 11beta,21-Dihydroxy-4-pregnene-3,20-dione; Kendall's compound B; Reichstein's substance H
316.226	C05454	-2.2	3alpha,7alpha,12alpha-Trihydroxy-5beta-cholestane; 5beta-Cholestane-3alpha,7alpha,12alpha-triol;
316.984	C01236	-2.31	D-Glucono-1,5-lactone 6-phosphate; 6-Phospho-D-glucono-1,5-lactone
317.207	C04717	-3.09	(9Z,11E)-(13S)-13-Hydroperoxyoctadeca-9,11-dienoic acid; (9Z,11E)-(13S)-13-Hydroperoxyoctadeca-9,11-dienoate;
317.207	C16677	-3.17	all-trans-4-Hydroxyretinoic acid
317.207	C16679	-2.92	all-trans-18-Hydroxyretinoic acid
317.207	C16680	-3.12	all-trans-5,6-Epoxyretinoic acid; all-trans-5,6-Epoxy-5,6-dihydroretinoic acid
317.243	C01953	-2.35	Pregnenolone; 5-Pregnen-3beta-ol-20-one; 3beta-Hydroxypregn-5-en-20-one
317.243	C03681	-3.46	5alpha-Pregnane-3,20-dione; 5alpha-Dihydroprogesterone
317.243	C05479	-3.35	5beta-Pregnane-3,20-dione
318.214	C01301	-3.66	3alpha,7alpha,12alpha-Trihydroxy-5beta-cholestan-26-al
318.214	C16677	-2.14	all-trans-4-Hydroxyretinoic acid
318.214	C16679	-1.92	all-trans-18-Hydroxyretinoic acid
318.214	C16680	-1.99	all-trans-5,6-Epoxyretinoic acid; all-trans-5,6-Epoxy-5,6-dihydroretinoic acid
318.293	C11695	-3.08	Anandamide; Arachidonylethanolamide; N-(5Z,8Z,11Z,14Z-Eicosatetraenoyl)-ethanolamine; AEA
318.938	C15650	-2.61	2,3-Diketo-5-methylthiopentyl-1-phosphate; 5-(Methylthio)-2,3-dioxopentyl phosphate
319.077	C06427	-1.75	(9Z,12Z,15Z)-Octadecatrienoic acid; alpha-Linolenic acid; 9,12,15-Octadecatrienoic acid; Linolenate; alpha-Linolenate
319.164	C02140	-2.16	Corticosterone; 11beta,21-Dihydroxy-4-pregnene-3,20-dione; Kendall's compound B; Reichstein's substance H
319.194	C00122	-1.88	Fumarate; Fumaric acid; trans-Butenedioic acid
319.224	C00909	-1.68	Leukotriene A4; LTA4; (7E,9E,11Z,14Z)-(5S,6S)-5,6-Epoxyeicosa-7,9,11,14-tetraenoic acid
319.224	C14825	-1.75	9(10)-EpOME; (9R,10S)-(12Z)-9,10-Epoxyoctadecenoic acid
319.224	C14826	-1.94	12(13)-EpOME; (12R,13S)-(9Z)-12,13-Epoxyoctadecenoic acid
320.153	C00750	-2.24	Spermine; N,N'-Bis(3-aminopropyl)-1,4-butanediamine
320.255	C04707	-3.08	(5Z,13E)-11alpha-Hydroxy-9,15-dioxoprost-13-enoate; (5Z)-(15S)-11alpha-Hydroxy-9,15-dioxoprosta-13-enoate
321.076	C05642	-2.69	Formyl-N-acetyl-5-methoxykynurenamine
321.181	C00410	-4.2	Progesterone; 4-Pregnene-3,20-dione
322.049	C02305	-2.45	Phosphocreatine; N-Phosphocreatine; Creatine phosphate
322.185	C00410	-3.23	Progesterone; 4-Pregnene-3,20-dione
323.043	C01762	-3.01	Xanthosine
323.075	C05382	-1.91	Sedoheptulose 7-phosphate; D-Sedoheptulose 7-phosphate; D-altro-Heptulose 7-phosphate
323.153	C00523	-4.28	Androsterone; 3alpha-Hydroxy-5alpha-androstan-17-one
323.197	C00410	-5.96	Progesterone; 4-Pregnene-3,20-dione
324.201	C00410	-5.35	Progesterone; 4-Pregnene-3,20-dione
324.288	C00836	-3.36	Sphinganine; Dihydrosphingosine; 2-Amino-1,3-dihydroxyoctadecane
325.116	C05300	-1.7	16alpha-Hydroxyestrone
325.212	C00410	-5.55	Progesterone; 4-Pregnene-3,20-dione
326.171	C02838	-2.5	L-Octanoylcarnitine
326.216	C00410	-5.33	Progesterone; 4-Pregnene-3,20-dione
327.014	C01236	-2.61	D-Glucono-1,5-lactone 6-phosphate; 6-Phospho-D-glucono-1,5-lactone
327.116	C01176	-3.76	17alpha-Hydroxyprogesterone; 17alpha-Hydroxy-4-pregnene-3,20-dione; Pregn-4-ene-3,20-dione-17-ol
327.116	C05298	-4.18	2-Hydroxyestrone
327.116	C05300	-4.36	16alpha-Hydroxyestrone
327.194	C05139	-2.17	16alpha-Hydroxydehydroepiandrosterone; 5-Androstene-3beta,16alpha-diol-17-one
327.194	C05291	-2.37	7alpha-Hydroxytestosterone
327.194	C05294	-1.97	19-Hydroxytestosterone; 17beta,19-Dihydroxyandrost-4-en-3-one
327.194	C05648	-2.43	5-Hydroxy-N-formylkynurenine; 5-Hydroxy-N-formyl-L-kynurenine
327.228	C00410	-5.85	Progesterone; 4-Pregnene-3,20-dione
327.288	C05341	-2.36	beta-Alanyl-L-lysine
328.232	C00410	-5.73	Progesterone; 4-Pregnene-3,20-dione

328.819	C00294	-1.91	Inosine
328.882	C15650	-2	2,3-Diketo-5-methylthiopentyl-1-phosphate; 5-(Methylthio)-2,3-dioxopentyl phosphate
329.138	C00735	-4	Cortisol; Hydrocortisone; 11beta,17alpha,21-Trihydroxy-4-pregnene-3,20-dione; Kendall's compound F
329.188	C06425	-2.91	Icosanoic acid; Eicosanoic acid; Arachidic acid
329.206	C00376	-1.71	Retinal; Vitamin A aldehyde; Retinene; all-trans-Retinal; all-trans-Vitamin A aldehyde; all-trans-Retinene
329.243	C00410	-6.25	Progesterone; 4-Pregnene-3,20-dione
329.304	C00735	-1.89	Cortisol; Hydrocortisone; 11beta,17alpha,21-Trihydroxy-4-pregnene-3,20-dione; Kendall's compound F
329.317	C00376	-2.96	Retinal; Vitamin A aldehyde; Retinene; all-trans-Retinal; all-trans-Vitamin A aldehyde; all-trans-Retinene
330.047	C00239	-2.44	dCMP; Deoxycytidylic acid; Deoxycytidine monophosphate; Deoxycytidylate; 2'-Deoxycytidine 5'-monophosphate
330.206	C00735	-4.58	Cortisol; Hydrocortisone; 11beta,17alpha,21-Trihydroxy-4-pregnene-3,20-dione; Kendall's compound F
330.246	C00410	-5.57	Progesterone; 4-Pregnene-3,20-dione
331.095	C03406	-2.34	N-(L-Arginino)succinate; 2-(Nomega-L-Arginino)succinate; L-Argininosuccinate; L-Argininosuccinic acid
331.095	C03722	-1.75	Quinolinate; Pyridine-2,3-dicarboxylate; Quinolinic acid; 2,3-Pyridinedicarboxylic acid
331.186	C03917	-2.34	Dihydrotestosterone; 17beta-Hydroxyandrostan-3-one; Androstanolone; 17beta-Hydroxy-5alpha-androstan-3-one
331.186	C04295	-2.03	Androstenediol; Androst-5-ene-3beta,17beta-diol; 3beta,17beta-Dihydroxyandrost-5-ene
331.186	C05293	-2.05	5beta-Dihydrotestosterone
331.918	C00468	-2.37	Estrone; 3-Hydroxy-1,3,5(10)-estratrien-17-one
332.007	C05382	-2.05	Sedoheptulose 7-phosphate; D-Sedoheptulose 7-phosphate; D-altro-Heptulose 7-phosphate
332.007	C06222	-1.96	Sedoheptulose 1-phosphate; altro-Heptulose 1-phosphate
332.082	C00130	-1.71	IMP; Inosinic acid; Inosine monophosphate; Inosine 5'-monophosphate; Inosine 5'-phosphate; 5'-Inosinate
332.177	C00327	-3.38	L-Citrulline; 2-Amino-5-ureidovaleric acid; Citrulline
332.259	C01953	-4.18	Pregnenolone; 5-Pregnen-3beta-ol-20-one; 3beta-Hydroxypregn-5-en-20-one
333.047	C00363	-1.93	dTDP; Deoxythymidine 5'-diphosphate
333.112	C00363	-2.93	dTDP; Deoxythymidine 5'-diphosphate
333.239	C02140	-3.84	Corticosterone; 11beta,21-Dihydroxy-4-pregnene-3,20-dione; Kendall's compound B; Reichstein's substance H
333.239	C05485	-2.37	21-Hydroxypregnenolone
334.047	C00130	-1.88	IMP; Inosinic acid; Inosine monophosphate; Inosine 5'-monophosphate; Inosine 5'-phosphate; 5'-Inosinate
334.082	C00523	-1.92	Androsterone; 3alpha-Hydroxy-5alpha-androstan-17-one
334.122	C00300	-2.62	Creatine; alpha-Methylguanidino acetic acid; Methylglycocyamine
334.168	C05476	-5.68	Tetrahydrocorticosterone
334.934	C04540	-2.48	N4-(Acetyl-beta-D-glucosaminyl)asparagine; N4-(beta-N-Acetyl-D-glucosaminyl)-L-asparagine
335.096	C00363	-2.83	dTDP; Deoxythymidine 5'-diphosphate
335.162	C05141	-4.59	Estriol; 1,3,5(10)-Estratriene-3,16-alpha,17beta-triol
335.218	C11695	-2.01	Anandamide; Arachidonylethanolamide; N-(5Z,8Z,11Z,14Z-Eicosatetraenoyl)-ethanolamine; AEA
335.252	C00468	-2.53	Estrone; 3-Hydroxy-1,3,5(10)-estratrien-17-one
336.04	C00130	-2.43	IMP; Inosinic acid; Inosine monophosphate; Inosine 5'-monophosphate; Inosine 5'-phosphate; 5'-Inosinate
336.169	C05141	-3.82	Estriol; 1,3,5(10)-Estratriene-3,16-alpha,17beta-triol
336.186	C14772	-1.74	5,6-DHET; (8Z,11Z,14Z)-5,6-Dihydroxyeicosa-8,11,14-trienoic acid
336.222	C11695	-1.78	Anandamide; Arachidonylethanolamide; N-(5Z,8Z,11Z,14Z-Eicosatetraenoyl)-ethanolamine; AEA
337.175	C00410	-2.97	Progesterone; 4-Pregnene-3,20-dione
337.233	C02165	-3.06	Leukotriene B4; (6Z,8E,10E,14Z)-(5S,12R)-5,12-Dihydroxyeicosa-6,8,10,14-tetraenoate
337.233	C05356	-3.42	5(S)-HPETE; 5(S)-Hydroperoxy-6-trans-8,11,14-cis-eicosatetraenoic acid
337.233	C05965	-4.08	12(S)-HPETE; (5Z,8Z,10E,14Z)-(12S)-12-Hydroperoxyicosa-5,8,10,14-tetraenoic acid
337.233	C05966	-3.57	15(S)-HPETE; (5Z,8Z,11Z,13E)-(15S)-15-Hydroperoxyicosa-5,8,11,13-tetraenoic acid
337.233	C14781	-3.65	15H-11,12-EETA; 15-Hydroxy-11,12-epoxyeicosatrienoic acid
337.233	C14813	-3.41	11H-14,15-EETA; 11-Hydroxy-14,15-EETA; 11-Hydroxy-14,15-epoxyeicosatrienoic acid
337.308	C00881	-1.98	Deoxycytidine; 2'-Deoxycytidine
338.178	C01953	-2.78	Pregnenolone; 5-Pregnen-3beta-ol-20-one; 3beta-Hydroxypregn-5-en-20-one
339.192	C00410	-4.87	Progesterone; 4-Pregnene-3,20-dione
339.221	C00762	-5.24	Cortisone; 17alpha,21-Dihydroxy-4-pregnene-3,11,20-trione; Kendall's compound E; Reichstein's substance Fa
339.286	C00468	-1.94	Estrone; 3-Hydroxy-1,3,5(10)-estratrien-17-one
340.136	C11695	-2.76	Anandamide; Arachidonylethanolamide; N-(5Z,8Z,11Z,14Z-Eicosatetraenoyl)-ethanolamine; AEA
341.016	C00160	-3.08	Glycolate; Glycolic acid; Hydroxyacetic acid
341.105	C00735	-1.97	Cortisol; Hydrocortisone; 11beta,17alpha,21-Trihydroxy-4-pregnene-3,20-dione; Kendall's compound F
341.207	C00410	-4.15	Progesterone; 4-Pregnene-3,20-dione
341.241	C05480	-2.73	Pregnanolone; 3alpha-Hydroxy-5beta-pregnan-20-one
341.241	C13712	-2.76	Allopregnanolone; 3alpha-Hydroxy-5alpha-pregnan-20-one; 3alpha-OH DHP
341.264	C05761	-1.75	Tetradecanoyl-[acp]; Tetradecanoyl-[acyl-carrier protein]; Myristoyl-[acyl-carrier protein]
341.342	C06425	-1.79	Icosanoic acid; Eicosanoic acid; Arachidic acid
342.031	C00330	-1.96	Deoxyguanosine; 2'-Deoxyguanosine
342.063	C04707	-2.99	(5Z,13E)-11alpha-Hydroxy-9,15-dioxoprost-13-enoate; (5Z)-(15S)-11alpha-Hydroxy-9,15-dioxoprosta-13-enoate
342.209	C00410	-3.31	Progesterone; 4-Pregnene-3,20-dione
342.246	C05480	-3.28	Pregnanolone; 3alpha-Hydroxy-5beta-pregnan-20-one
342.246	C13712	-3.31	Allopregnanolone; 3alpha-Hydroxy-5alpha-pregnan-20-one; 3alpha-OH DHP
342.342	C06425	-2.5	Icosanoic acid; Eicosanoic acid; Arachidic acid
343.2	C05490	-8.36	11-Dehydrocorticosterone
343.221	C00951	-2.2	Estradiol-17beta; Estradiol; beta-Estradiol
344.205	C00410	-6.99	Progesterone; 4-Pregnene-3,20-dione
345.208	C00535	-3.23	Testosterone; 17beta-Hydroxy-4-androsten-3-one
345.208	C05490	-5.53	11-Dehydrocorticosterone
345.236	C00410	-4.76	Progesterone; 4-Pregnene-3,20-dione
346.237	C00410	-2.12	Progesterone; 4-Pregnene-3,20-dione
347.065	C00655	-2.16	Xanthosine 5'-phosphate; Xanthylic acid; XMP; (9-D-Ribosylxanthine)-5'-phosphate
347.217	C02140	-3.59	Corticosterone; 11beta,21-Dihydroxy-4-pregnene-3,20-dione; Kendall's compound B; Reichstein's substance H
347.217	C05477	-3.6	21-Hydroxy-5beta-pregnane-3,11,20-trione
347.217	C05488	-2.3	11-Deoxycortisol; Cortodoxone
347.217	C05497	-2.84	21-Deoxycortisol; 4-Pregnene-11beta,17alpha-diol-3,20-dione; 11beta,17alpha-Dihydroxyprogesterone
347.291	C00523	-2.36	Androsterone; 3alpha-Hydroxy-5alpha-androstan-17-one
348.192	C01301	-2.94	3alpha,7alpha,12alpha-Trihydroxy-5beta-cholestan-26-al
348.218	C05138	-2.9	17alpha-Hydroxypregnenolone
348.286	C11695	-2.97	Anandamide; Arachidonylethanolamide; N-(5Z,8Z,11Z,14Z-Eicosatetraenoyl)-ethanolamine; AEA
349.056	C00300	-1.87	Creatine; alpha-Methylguanidino acetic acid; Methylglycocyamine
349.224	C05476	-1.92	Tetrahydrocorticosterone

349.224	C05477	-2.24	21-Hydroxy-5beta-pregnane-3,11,20-trione
349.267	C00523	-2.46	Androsterone; 3alpha-Hydroxy-5alpha-androstan-17-one
350.058	C05922	-2.06	Formamidopyrimidine nucleoside triphosphate
350.187	C05959	-2.88	11-epi-Prostaglandin F2alpha; 11-epi-Prostaglandin F2a; 11-epi-PGF2alpha; 11-epi-PGF2a
350.23	C01301	-2.59	3alpha,7alpha,12alpha-Trihydroxy-5beta-cholestan-26-al
351.2	C01301	-3.94	3alpha,7alpha,12alpha-Trihydroxy-5beta-cholestan-26-al
352.057	C00363	-3.14	dTDP; Deoxythymidine 5'-diphosphate
352.202	C04707	-3.79	(5Z,13E)-11alpha-Hydroxy-9,15-dioxoprost-13-enoate; (5Z)-(15S)-11alpha-Hydroxy-9,15-dioxoprosta-13-enoate
352.222	C13713	-2.4	Allotetrahydrodeoxycorticosterone; 5alpha-THDOC; 3alpha,21-Dihydroxy-5alpha-pregnan-20-one
353.073	C00921	-3.51	Dihydropteroate; 7,8-Dihydropteroate
353.208	C01176	-5.16	17alpha-Hydroxyprogesterone; 17alpha-Hydroxy-4-pregnene-3,20-dione
353.208	C03205	-7.3	11-Deoxycorticosterone; Deoxycorticosterone; Cortexone; 21-Hydroxy-4-pregnene-3,20-dione; Desoxycortone; DOC
353.208	C05498	-6.99	11beta-Hydroxyprogesterone
354.158	C00526	-2.3	Deoxyuridine; 2-Deoxyuridine; 2'-Deoxyuridine
354.171	C05648	-2.07	5-Hydroxy-N-formylkynurenine; 5-Hydroxy-N-formyl-L-kynurenine
354.892	C00197	-2.45	3-Phospho-D-glycerate; D-Glycerate 3-phosphate; 3-Phospho-(R)-glycerate; 3-Phosphoglycerate
355.1	C02990	-2.79	L-Palmitoylcarnitine
355.188	C00410	-3.48	Progesterone; 4-Pregnene-3,20-dione
355.218	C05490	-5.31	11-Dehydrocorticosterone
356.19	C00410	-4.01	Progesterone; 4-Pregnene-3,20-dione
356.231	C04707	-4.18	(5Z,13E)-11alpha-Hydroxy-9,15-dioxoprost-13-enoate; (5Z)-(15S)-11alpha-Hydroxy-9,15-dioxoprosta-13-enoate
357.202	C00523	-2.51	Androsterone; 3alpha-Hydroxy-5alpha-androstan-17-one
357.232	C00535	-2.91	Testosterone; 17beta-Hydroxy-4-androsten-3-one
358.032	C00104	-2.78	IDP; Inosine 5'-diphosphate; Inosine diphosphate
358.208	C05642	-2.67	Formyl-N-acetyl-5-methoxykynurenamine
358.232	C04707	-3.55	(5Z,13E)-11alpha-Hydroxy-9,15-dioxoprost-13-enoate; (5Z)-(15S)-11alpha-Hydroxy-9,15-dioxoprosta-13-enoate
359.124	C05711	-2.41	gamma-Glutamyl-beta-cyanoalanine
359.215	C00523	-2.16	Androsterone; 3alpha-Hydroxy-5alpha-androstan-17-one
360.219	C05480	-2.19	Pregnanolone; 3alpha-Hydroxy-5beta-pregnan-20-one
361.015	C00364	-1.68	dTMP; Thymidine 5'-phosphate; Deoxythymidine 5'-phosphate; Thymidylic acid; 5'-Thymidylic acid
361.104	C00363	-1.8	dTDP; Deoxythymidine 5'-diphosphate
361.167	C00523	-1.84	Androsterone; 3alpha-Hydroxy-5alpha-androstan-17-one
361.205	C00762	-4.15	Cortisone; 17alpha,21-Dihydroxy-4-pregnene-3,11,20-trione; Kendall's compound E; Reichstein's substance Fa
361.205	C01780	-5.37	Aldosterone; 11beta,21-Dihydroxy-3,20-dioxo-4-pregnen-18-al
361.233	C02140	-2.06	Corticosterone; 11beta,21-Dihydroxy-4-pregnene-3,20-dione; Kendall's compound B; Reichstein's substance H
361.27	C00535	-4.14	Testosterone; 17beta-Hydroxy-4-androsten-3-one
361.885	C05341	-1.82	beta-Alanyl-L-lysine
361.973	C00062	-4.39	L-Arginine; (S)-2-Amino-5-guanidinovaleric acid; L-Arg
362.885	C03232	-2.07	3-Phosphonooxypyruvate; 3-Phosphonooxypyruvic acid; 3-Phosphohydroxypyruvate
363.125	C02999	-1.92	N-Acetylmuramoyl-Ala; N-Acetyl-D-muramoyl-L-alanine
363.217	C03588	-1.93	4,5alpha-Dihydrocortisone
363.217	C05469	-1.77	17alpha,21-Dihydroxy-5beta-pregnane-3,11,20-trione; 5beta-Pregnane-17alpha,21-diol-3,11,20-trione
363.245	C00523	-2.42	Androsterone; 3alpha-Hydroxy-5alpha-androstan-17-one
364.017	C00167	-1.83	UDP-glucuronate; UDPglucuronate; UDP-D-glucuronate; UDP-alpha-D-glucuronate
364.017	C00364	-3.05	dTMP; Thymidine 5'-phosphate; Deoxythymidine 5'-phosphate; Thymidylic acid; 5'-Thymidylic acid
364.017	C00364	-3.09	dTMP; Thymidine 5'-phosphate; Deoxythymidine 5'-phosphate; Thymidylic acid; 5'-Thymidylic acid
364.178	C04717	-1.82	(9Z,11E)-(13S)-13-Hydroperoxyoctadeca-9,11-dienoic acid; (9Z,11E)-(13S)-13-Hydroperoxyoctadeca-9,11-dienoate
364.32	C00523	-3.06	Androsterone; 3alpha-Hydroxy-5alpha-androstan-17-one
365.196	C02838	-1.97	L-Octanoylcarnitine
366.197	C16677	-2.21	all-trans-4-Hydroxyretinoic acid
367.12	C00294	-1.9	Inosine
367.244	C05472	-1.95	Urocortisol; Tetrahydrocortisol; 5beta-Pregnane-3alpha,11beta,17alpha,21-tetrol-20-one
367.964	C05699	-1.68	L-Selenocystathionine
368.198	C05454	-2.33	3alpha,7alpha,12alpha-Trihydroxy-5beta-cholestane; 5beta-Cholestane-3alpha,7alpha,12alpha-triol
368.249	C05472	-5.59	Urocortisol; Tetrahydrocortisol; 5beta-Pregnane-3alpha,11beta,17alpha,21-tetrol-20-one
369.199	C02140	-5.78	Corticosterone; 11beta,21-Dihydroxy-4-pregnene-3,20-dione; Kendall's compound B; Reichstein's substance H
369.199	C05477	-6.09	21-Hydroxy-5beta-pregnane-3,11,20-trione
369.199	C05488	-4.09	11-Deoxycortisol; Cortodoxone
369.199	C05497	-4.43	21-Deoxycortisol; 4-Pregnene-11beta,17alpha-diol-3,20-dione; 11beta,17alpha-Dihydroxyprogesterone
370.003	C05922	-2.18	Formamidopyrimidine nucleoside triphosphate
371.164	C13713	-2.77	Allotetrahydrodeoxycorticosterone; 5alpha-THDOC; 3alpha,21-Dihydroxy-5alpha-pregnan-20-one
371.194	C02140	-4.79	Corticosterone; 11beta,21-Dihydroxy-4-pregnene-3,20-dione; Kendall's compound B; Reichstein's substance H
371.194	C05138	-2.29	17alpha-Hydroxypregnenolone
371.194	C05485	-3.39	21-Hydroxypregnenolone
371.217	C05475	-4.09	11beta,21-Dihydroxy-5beta-pregnane-3,20-dione; 5beta-Pregnane-11beta,21-diol-3,20-dione
371.217	C05478	-4.42	3alpha,21-Dihydroxy-5beta-pregnane-11,20-dione; 5beta-Pregnane-3alpha,21-diol-11,20-dione
371.217	C05487	-2.46	17alpha,21-Dihydroxypregnenolone
372.002	C00130	-2.07	IMP; Inosinic acid; Inosine monophosphate; Inosine 5'-monophosphate; Inosine 5'-phosphate; 5'-Inosinate
372.208	C03640	-2.15	Sphingosyl-phosphocholine
372.994	C00130	-2.03	IMP; Inosinic acid; Inosine monophosphate; Inosine 5'-monophosphate; Inosine 5'-phosphate; 5'-Inosinate
373.237	C05476	-3.91	Tetrahydrocorticosterone
374.04	C00363	-2.91	dTDP; Deoxythymidine 5'-diphosphate
374.202	C01301	-3.99	3alpha,7alpha,12alpha-Trihydroxy-5beta-cholestan-26-al
374.27	C05476	-3.11	Tetrahydrocorticosterone
375.12	C00104	-2.59	IDP; Inosine 5'-diphosphate; Inosine diphosphate
375.222	C13713	-5.49	Allotetrahydrodeoxycorticosterone; 5alpha-THDOC; 3alpha,21-Dihydroxy-5alpha-pregnan-20-one
375.248	C01190	-2.02	Glucosylceramide; Glucocerebroside; beta-D-Glucosyl-(1<->1)-ceramide; D-Glucosyl-N-acylsphingosine
376.009	C00049	-2.05	L-Aspartate; L-Aspartic acid; 2-Aminosuccinic acid; L-Asp
376.146	C05648	-2.18	5-Hydroxy-N-formylkynurenine; 5-Hydroxy-N-formyl-L-kynurenine
376.146	C05692	-2.28	Se-Adenosyl-L-selenohomocysteine; Se-Adenosylselenohomocysteine
376.194	C04707	-2.72	(5Z,13E)-11alpha-Hydroxy-9,15-dioxoprost-13-enoate; (5Z)-(15S)-11alpha-Hydroxy-9,15-dioxoprosta-13-enoate
376.224	C05138	-3.67	17alpha-Hydroxypregnenolone
376.253	C01190	-1.74	Glucosylceramide; Glucocerebroside; beta-D-Glucosyl-(1<->1)-ceramide; D-Glucosyl-N-acylsphingosine

377.085	C00363	-3.55	dTDP; Deoxythymidine 5'-diphosphate
377.187	C14772	-2.77	5,6-DHET; (8Z,11Z,14Z)-5,6-Dihydroxyeicosa-8,11,14-trienoic acid
377.206	C00735	-4.82	Cortisol; Hydrocortisone; 11beta,17alpha,21-Trihydroxy-4-pregnene-3,20-dione; Kendall's compound F
377.214	C05138	-2.21	17alpha-Hydroxypregnenolone
377.993	C05382	-1.98	Sedoheptulose 7-phosphate; D-Sedoheptulose 7-phosphate; D-altro-Heptulose 7-phosphate
378.182	C04707	-2.85	(5Z,13E)-11alpha-Hydroxy-9,15-dioxoprost-13-enoate; (5Z)-(15S)-11alpha-Hydroxy-9,15-dioxoprosta-13-enoate
378.194	C14772	-2.46	5,6-DHET; (8Z,11Z,14Z)-5,6-Dihydroxyeicosa-8,11,14-trienoic acid
378.216	C14772	-1.86	5,6-DHET; (8Z,11Z,14Z)-5,6-Dihydroxyeicosa-8,11,14-trienoic acid
378.216	C16677	-1.79	all-trans-4-Hydroxyretinoic acid
378.226	C00735	-2.23	Cortisol; Hydrocortisone; 11beta,17alpha,21-Trihydroxy-4-pregnene-3,20-dione; Kendall's compound F
378.99	C05382	-2.25	Sedoheptulose 7-phosphate; D-Sedoheptulose 7-phosphate; D-altro-Heptulose 7-phosphate
379.195	C02838	-1.96	L-Octanoylcarnitine
379.22	C05476	-3.43	Tetrahydrocorticosterone
381.196	C04717	-2.35	(9Z,11E)-(13S)-13-Hydroperoxyoctadeca-9,11-dienoic acid; (9Z,11E)-(13S)-13-Hydroperoxyoctadeca-9,11-dienoate
382.198	C04540	-2	N4-(Acetyl-beta-D-glucosaminyl)asparagine; N4-(beta-N-Acetyl-D-glucosaminyl)-L-asparagine
383.026	C06198	-1.83	P1,P4-Bis(5'-uridyl) tetraphosphate; UppppU
383.115	C00031	-2.59	D-Glucose; Grape sugar; Dextrose; Glucose; D-Glucopyranose
383.211	C05476	-5.33	Tetrahydrocorticosterone
384.118	C00031	-3.21	D-Glucose; Grape sugar; Dextrose; Glucose; D-Glucopyranose
384.169	C01301	-2.07	3alpha,7alpha,12alpha-Trihydroxy-5beta-cholestan-26-al
385.121	C00031	-2.77	D-Glucose; Grape sugar; Dextrose; Glucose; D-Glucopyranose
385.182	C02140	-8.01	Corticosterone; 11beta,21-Dihydroxy-4-pregnene-3,20-dione; Kendall's compound B; Reichstein's substance H
385.182	C05477	-8.54	21-Hydroxy-5beta-pregnane-3,11,20-trione
385.182	C05488	-5.91	11-Deoxycortisol; Cortodoxone
385.182	C05497	-6.91	21-Deoxycortisol; 4-Pregnene-11beta,17alpha-diol-3,20-dione; 11beta,17alpha-Dihydroxyprogesterone
386.262	C01190	-2.1	Glucosylceramide; Glucocerebroside; beta-D-Glucosyl-(1<->1)-ceramide; D-Glucosyl-N-acylsphingosine
387.183	C02140	-6.43	Corticosterone; 11beta,21-Dihydroxy-4-pregnene-3,20-dione; Kendall's compound B; Reichstein's substance H
387.183	C05477	-7.13	21-Hydroxy-5beta-pregnane-3,11,20-trione
387.183	C05488	-5.9	11-Deoxycortisol; Cortodoxone
387.183	C05497	-6.07	21-Deoxycortisol; 4-Pregnene-11beta,17alpha-diol-3,20-dione; 11beta,17alpha-Dihydroxyprogesterone
387.249	C00523	-3.7	Androsterone; 3alpha-Hydroxy-5alpha-androstan-17-one
388.178	C04707	-3.06	(5Z,13E)-11alpha-Hydroxy-9,15-dioxoprost-13-enoate; (5Z)-(15S)-11alpha-Hydroxy-9,15-dioxoprosta-13-enoate
388.273	C14772	-2.57	5,6-DHET; (8Z,11Z,14Z)-5,6-Dihydroxyeicosa-8,11,14-trienoic acid
389.169	C04707	-1.75	(5Z,13E)-11alpha-Hydroxy-9,15-dioxoprost-13-enoate; (5Z)-(15S)-11alpha-Hydroxy-9,15-dioxoprosta-13-enoate
389.198	C00376	-1.76	Retinal; Vitamin A aldehyde; Retinene; all-trans-Retinal; all-trans-Vitamin A aldehyde; all-trans-Retinene
389.339	C00836	-2.26	Sphinganine; Dihydrosphingosine; 2-Amino-1,3-dihydroxyoctadecane
390.174	C04707	-3.3	(5Z,13E)-11alpha-Hydroxy-9,15-dioxoprost-13-enoate; (5Z)-(15S)-11alpha-Hydroxy-9,15-dioxoprosta-13-enoate
390.174	C04707	-3.66	(5Z,13E)-11alpha-Hydroxy-9,15-dioxoprost-13-enoate; (5Z)-(15S)-11alpha-Hydroxy-9,15-dioxoprosta-13-enoate
391.11	C00104	-2.74	IDP; Inosine 5'-diphosphate; Inosine diphosphate
391.11	C05692	-2.53	Se-Adenosyl-L-selenohomocysteine; Se-Adenosylselenohomocysteine
391.221	C00735	-2.72	Cortisol; Hydrocortisone; 11beta,17alpha,21-Trihydroxy-4-pregnene-3,20-dione; Kendall's compound F
392.188	C01301	-4.69	3alpha,7alpha,12alpha-Trihydroxy-5beta-cholestan-26-al
393.207	C05959	-3.02	11-epi-Prostaglandin F2alpha; 11-epi-Prostaglandin F2a; 11-epi-PGF2alpha; 11-epi-PGF2a
393.207	C14782	-3.03	11,12,15-THETA; 11,12,15-Trihydroxyicosatrienoic acid
393.207	C14814	-2.97	11,14,15-THETA; 11,14,15-Trihydroxyicosatrienoic acid; (5Z,8Z,12E)-11,14,15-Trihydroxyeicosa-5,8,12-trienoic acid
393.259	C02838	-3.22	L-Octanoylcarnitine
394.212	C05959	-2.8	11-epi-Prostaglandin F2alpha; 11-epi-Prostaglandin F2a; 11-epi-PGF2alpha; 11-epi-PGF2a
394.212	C14782	-2.8	11,12,15-THETA; 11,12,15-Trihydroxyicosatrienoic acid
394.212	C14814	-2.83	11,14,15-THETA; 11,14,15-Trihydroxyicosatrienoic acid; (5Z,8Z,12E)-11,14,15-Trihydroxyeicosa-5,8,12-trienoic acid
394.259	C02165	-1.7	Leukotriene B4; (6Z,8E,10E,14Z)-(5S,12R)-5,12-Dihydroxyeicosa-6,8,10,14-tetraenoate
395.171	C01301	-2.45	3alpha,7alpha,12alpha-Trihydroxy-5beta-cholestan-26-al
396.225	C01301	-1.7	3alpha,7alpha,12alpha-Trihydroxy-5beta-cholestan-26-al
396.805	C00294	-1.98	Inosine
396.922	C00117	-2.43	D-Ribose 5-phosphate; Ribose 5-phosphate
397.107	C03758	-1.98	Dopamine; 4-(2-Aminoethyl)-1,2-benzenediol; 4-(2-Aminoethyl)benzene-1,2-diol; 3,4-Dihydroxyphenethylamine
397.183	C05648	-1.8	5-Hydroxy-N-formylkynurenine; 5-Hydroxy-N-formyl-L-kynurenine
397.237	C01301	-2.35	3alpha,7alpha,12alpha-Trihydroxy-5beta-cholestan-26-al
398.108	C02051	-3	Lipoylprotein; H-Protein-lipoyllysine; [GCSH]-N6-lipoyl-L-lysine; [GcvH]-N6-lipoyl-L-lysine
398.199	C02838	-2.03	L-Octanoylcarnitine
398.241	C04707	-3.17	(5Z,13E)-11alpha-Hydroxy-9,15-dioxoprost-13-enoate; (5Z)-(15S)-11alpha-Hydroxy-9,15-dioxoprosta-13-enoate
399.088	C00031	-2.03	D-Glucose; Grape sugar; Dextrose; Glucose; D-Glucopyranose
399.182	C05761	-1.77	Tetradecanoyl-[acp]; Tetradecanoyl-[acyl-carrier protein]; Myristoyl-[acyl-carrier protein]
400.092	C00031	-2.37	D-Glucose; Grape sugar; Dextrose; Glucose; D-Glucopyranose
400.211	C01301	-2.66	3alpha,7alpha,12alpha-Trihydroxy-5beta-cholestan-26-al
401.088	C00031	-2.55	D-Glucose; Grape sugar; Dextrose; Glucose; D-Glucopyranose
401.17	C00735	-3.02	Cortisol; Hydrocortisone; 11beta,17alpha,21-Trihydroxy-4-pregnene-3,20-dione; Kendall's compound F
401.17	C01124	-3.94	18-Hydroxycorticosterone
401.17	C03588	-2.9	4,5alpha-Dihydrocortisone
401.17	C05469	-2.98	17alpha,21-Dihydroxy-5beta-pregnane-3,11,20-trione; 5beta-Pregnane-17alpha,21-diol-3,11,20-trione
401.17	C05473	-3.11	11beta,21-Dihydroxy-3,20-oxo-5beta-pregnan-18-al
401.225	C05138	-1.71	17alpha-Hydroxypregnenolone
401.89	C06425	-1.66	Icosanoic acid; Eicosanoic acid; Arachidic acid
402.175	C05454	-2.12	3alpha,7alpha,12alpha-Trihydroxy-5beta-cholestane; 5beta-Cholestane-3alpha,7alpha,12alpha-triol
402.248	C02835	-2.38	Imidazole-4-acetate; Imidazoleacetic acid; 4-Imidazoleacetate
403.176	C00735	-3.82	Cortisol; Hydrocortisone; 11beta,17alpha,21-Trihydroxy-4-pregnene-3,20-dione; Kendall's compound F
403.176	C00735	-3.89	Cortisol; Hydrocortisone; 11beta,17alpha,21-Trihydroxy-4-pregnene-3,20-dione; Kendall's compound F
403.176	C01124	-4.68	18-Hydroxycorticosterone
403.176	C01124	-4.97	18-Hydroxycorticosterone
403.176	C03588	-4.17	4,5alpha-Dihydrocortisone
403.176	C03588	-4.55	4,5alpha-Dihydrocortisone
403.176	C05469	-3.89	17alpha,21-Dihydroxy-5beta-pregnane-3,11,20-trione; 5beta-Pregnane-17alpha,21-diol-3,11,20-trione
403.176	C05469	-4.5	17alpha,21-Dihydroxy-5beta-pregnane-3,11,20-trione; 5beta-Pregnane-17alpha,21-diol-3,11,20-trione
403.176	C05473	-4.29	11beta,21-Dihydroxy-3,20-oxo-5beta-pregnan-18-al

403.176	C05473	-4.3	11beta,21-Dihydroxy-3,20-oxo-5beta-pregnan-18-al
405.096	C00279	-3.31	D-Erythrose 4-phosphate
406.185	C04707	-2.74	(5Z,13E)-11alpha-Hydroxy-9,15-dioxoprost-13-enoate; (5Z)-(15S)-11alpha-Hydroxy-9,15-dioxoprosta-13-enoate
406.269	C00168	-1.86	Hydroxypyruvate; Hydroxypyruvic acid; 3-Hydroxypyruvate; 3-Hydroxypyruvic acid
406.953	C00363	-1.69	dTDP; Deoxythymidine 5'-diphosphate
407.092	C05642	-1.83	Formyl-N-acetyl-5-methoxykynurenamine
407.202	C00735	-5.74	Cortisol; Hydrocortisone; 11beta,17alpha,21-Trihydroxy-4-pregnene-3,20-dione; Kendall's compound F
407.273	C00735	-4.83	Cortisol; Hydrocortisone; 11beta,17alpha,21-Trihydroxy-4-pregnene-3,20-dione; Kendall's compound F
409.218	C05959	-2.11	11-epi-Prostaglandin F2alpha; 11-epi-Prostaglandin F2a; 11-epi-PGF2alpha; 11-epi-PGF2a
410.183	C03640	-2.02	Sphingosyl-phosphocholine
412.229	C05452	-1.66	3alpha,7alpha-Dihydroxy-5beta-cholestane; 5beta-Cholestane-3alpha,7alpha-diol
413.047	C00363	-2.05	dTDP; Deoxythymidine 5'-diphosphate
415.105	C00255	-1.81	Riboflavin; Lactoflavin; 7,8-Dimethyl-10-ribitylisoalloxazine; Vitamin B2
416.268	C02838	-1.9	L-Octanoylcarnitine
417.159	C05476	-3.91	Tetrahydrocorticosterone
417.198	C05476	-3.61	Tetrahydrocorticosterone
418.029	C00130	-1.94	IMP; Inosinic acid; Inosine monophosphate; Inosine 5'-monophosphate; Inosine 5'-phosphate; 5'-Inosinate
418.243	C05959	-2.88	11-epi-Prostaglandin F2alpha; 11-epi-Prostaglandin F2a; 11-epi-PGF2alpha; 11-epi-PGF2a
419.176	C00735	-4.82	Cortisol; Hydrocortisone; 11beta,17alpha,21-Trihydroxy-4-pregnene-3,20-dione; Kendall's compound F
420.883	C00197	-2.07	3-Phospho-D-glycerate; D-Glycerate 3-phosphate; 3-Phospho-(R)-glycerate; 3-Phosphoglycerate
421.142	C04717	-2.57	(9Z,11E)-(13S)-13-Hydroperoxyoctadeca-9,11-dienoic acid; (9Z,11E)-(13S)-13-Hydroperoxyoctadeca-9,11-dienoate
421.204	C05959	-2.14	11-epi-Prostaglandin F2alpha; 11-epi-Prostaglandin F2a; 11-epi-PGF2alpha; 11-epi-PGF2a
421.329	C00931	-2.48	Porphobilinogen
422.201	C01301	-2.8	3alpha,7alpha,12alpha-Trihydroxy-5beta-cholestan-26-al
422.29	C01301	-2.6	3alpha,7alpha,12alpha-Trihydroxy-5beta-cholestan-26-al
422.88	C00197	-3.22	3-Phospho-D-glycerate; D-Glycerate 3-phosphate; 3-Phospho-(R)-glycerate; 3-Phosphoglycerate
426.22	C04540	-1.87	N4-(Acetyl-beta-D-glucosaminyl)asparagine; N4-(beta-N-Acetyl-D-glucosaminyl)-L-asparagine
428.912	C00119	-2.78	5-Phospho-alpha-D-ribose 1-diphosphate; 5-Phosphoribosyl diphosphate; 5-Phosphoribosyl 1-pyrophosphate; PRPP
429.088	C05526	-2.82	S-Glutathionyl-L-cysteine
429.15	C11695	-2.94	Anandamide; Arachidonylethanolamide; N-(5Z,8Z,11Z,14Z-Eicosatetraenoyl)-ethanolamine; AEA
430.242	C05648	-2.35	5-Hydroxy-N-formylkynurenine; 5-Hydroxy-N-formyl-L-kynurenine
430.789	C01301	-2.25	3alpha,7alpha,12alpha-Trihydroxy-5beta-cholestan-26-al
430.912	C00119	-2.8	5-Phospho-alpha-D-ribose 1-diphosphate; 5-Phosphoribosyl diphosphate; 5-Phosphoribosyl 1-pyrophosphate; PRPP
431.916	C00119	-2.27	5-Phospho-alpha-D-ribose 1-diphosphate; 5-Phosphoribosyl diphosphate; 5-Phosphoribosyl 1-pyrophosphate; PRPP
432.195	C05699	-1.7	L-Selenocystathionine
433.005	C05583	-2.78	3-Methoxy-4-hydroxyphenylglycolaldehyde
434.961	C00655	-1.82	Xanthosine 5'-phosphate; Xanthylic acid; XMP; (9-D-Ribosylxanthine)-5'-phosphate
435.09	C00082	-2.13	L-Tyrosine; (S)-3-(p-Hydroxyphenyl)alanine; (S)-2-Amino-3-(p-hydroxyphenyl)propionic acid; Tyrosine
435.321	C00735	-4.9	Cortisol; Hydrocortisone; 11beta,17alpha,21-Trihydroxy-4-pregnene-3,20-dione; Kendall's compound F
436.212	C03640	-1.91	Sphingosyl-phosphocholine
437.136	C00624	-2	N-Acetyl-L-glutamate; N-Acetyl-L-glutamic acid
437.334	C05141	-3.56	Estriol; 1,3,5(10)-Estratriene-3,16-alpha,17beta-triol
439.263	C05454	-2.97	3alpha,7alpha,12alpha-Trihydroxy-5beta-cholestane; 5beta-Cholestane-3alpha,7alpha,12alpha-triol;
439.349	C05476	-7.99	Tetrahydrocorticosterone
440.003	C00363	-2.18	dTDP; Deoxythymidine 5'-diphosphate
440.257	C05959	-2.82	11-epi-Prostaglandin F2alpha; 11-epi-Prostaglandin F2a; 11-epi-PGF2alpha; 11-epi-PGF2a
440.28	C05454	-3.16	3alpha,7alpha,12alpha-Trihydroxy-5beta-cholestane; 5beta-Cholestane-3alpha,7alpha,12alpha-triol;
440.986	C00130	-1.81	IMP; Inosinic acid; Inosine monophosphate; Inosine 5'-monophosphate; Inosine 5'-phosphate; 5'-Inosinate
441.263	C01301	-2.84	3alpha,7alpha,12alpha-Trihydroxy-5beta-cholestan-26-al
441.352	C05452	-3.86	3alpha,7alpha-Dihydroxy-5beta-cholestane; 5beta-Cholestane-3alpha,7alpha-diol
442.265	C01301	-2.41	3alpha,7alpha,12alpha-Trihydroxy-5beta-cholestan-26-al
442.991	C00363	-2	dTDP; Deoxythymidine 5'-diphosphate
443.332	C01190	-1.73	Glucosylceramide; Glucocerebroside; beta-D-Glucosyl-(1<->1)-ceramide; D-Glucosyl-N-acylsphingosine
443.332	C05452	-4.01	3alpha,7alpha-Dihydroxy-5beta-cholestane; 5beta-Cholestane-3alpha,7alpha-diol
443.995	C01031	-1.72	S-Formylglutathione
445.222	C02838	-3.01	L-Octanoylcarnitine
445.26	C00468	-1.69	Estrone; 3-Hydroxy-1,3,5(10)-estratrien-17-one
446.186	C05648	-1.74	5-Hydroxy-N-formylkynurenine; 5-Hydroxy-N-formyl-L-kynurenine
447.241	C05959	-1.91	11-epi-Prostaglandin F2alpha; 11-epi-Prostaglandin F2a; 11-epi-PGF2alpha; 11-epi-PGF2a
447.27	C01301	-4.54	3alpha,7alpha,12alpha-Trihydroxy-5beta-cholestan-26-al
451.287	C01301	-2.65	3alpha,7alpha,12alpha-Trihydroxy-5beta-cholestan-26-al
451.353	C05138	-3.11	17alpha-Hydroxypregnenolone
452.298	C01301	-3.35	3alpha,7alpha,12alpha-Trihydroxy-5beta-cholestan-26-al
452.36	C13713	-2.38	Allotetrahydrodeoxycorticosterone; 5alpha-THDOC; 3alpha,21-Dihydroxy-5alpha-pregnan-20-one
453.233	C04707	-4.22	(5Z,13E)-11alpha-Hydroxy-9,15-dioxoprost-13-enoate; (5Z)-(15S)-11alpha-Hydroxy-9,15-dioxoprosta-13-enoate
453.298	C01301	-3.51	3alpha,7alpha,12alpha-Trihydroxy-5beta-cholestan-26-al
454.238	C04707	-4.22	(5Z,13E)-11alpha-Hydroxy-9,15-dioxoprost-13-enoate; (5Z)-(15S)-11alpha-Hydroxy-9,15-dioxoprosta-13-enoate
455.231	C04707	-4.03	(5Z,13E)-11alpha-Hydroxy-9,15-dioxoprost-13-enoate; (5Z)-(15S)-11alpha-Hydroxy-9,15-dioxoprosta-13-enoate
455.95	C00104	-1.99	IDP; Inosine 5'-diphosphate; Inosine diphosphate
458.344	C16677	-1.94	all-trans-4-Hydroxyretinoic acid
459.275	C06425	-2.14	Icosanoic acid; Eicosanoic acid; Arachidic acid
459.32	C05454	-2.66	3alpha,7alpha,12alpha-Trihydroxy-5beta-cholestane; 5beta-Cholestane-3alpha,7alpha,12alpha-triol
462.966	C00363	-2.58	dTDP; Deoxythymidine 5'-diphosphate
463.303	C04707	-4.19	(5Z,13E)-11alpha-Hydroxy-9,15-dioxoprost-13-enoate; (5Z)-(15S)-11alpha-Hydroxy-9,15-dioxoprosta-13-enoate
464.306	C04707	-4.99	(5Z,13E)-11alpha-Hydroxy-9,15-dioxoprost-13-enoate; (5Z)-(15S)-11alpha-Hydroxy-9,15-dioxoprosta-13-enoate
465.129	C00081	-2.09	ITP; Inosine 5'-triphosphate; Inosine triphosphate; Inosine tripolyphosphate
465.299	C01301	-3.47	3alpha,7alpha,12alpha-Trihydroxy-5beta-cholestan-26-al
466.975	C00075	-1.94	UTP; Uridine 5'-triphosphate; Uridine triphosphate
467.206	C01190	-1.67	Glucosylceramide; Glucocerebroside; beta-D-Glucosyl-(1<->1)-ceramide; D-Glucosyl-N-acylsphingosine
468.953	C00363	-4.21	dTDP; Deoxythymidine 5'-diphosphate
469.208	C05452	-2.62	3alpha,7alpha-Dihydroxy-5beta-cholestane; 5beta-Cholestane-3alpha,7alpha-diol
469.256	C05959	-3.06	11-epi-Prostaglandin F2alpha; 11-epi-Prostaglandin F2a; 11-epi-PGF2alpha; 11-epi-PGF2a
471.058	C00363	-2.64	dTDP; Deoxythymidine 5'-diphosphate

471.263	C03640	-2.38	Sphingosyl-phosphocholine
471.38	C01301	-2.83	3alpha,7alpha,12alpha-Trihydroxy-5beta-cholestan-26-al
473.306	C01301	-3.16	3alpha,7alpha,12alpha-Trihydroxy-5beta-cholestan-26-al
473.306	C04554	-2.66	3alpha,7alpha-Dihydroxy-5beta-cholestanate; 3alpha,7alpha-Dihydroxy-5beta-cholestanoate
473.34	C05452	-2.48	3alpha,7alpha-Dihydroxy-5beta-cholestane; 5beta-Cholestane-3alpha,7alpha-diol
475.256	C02166	-2.25	Leukotriene C4; LTC4
475.311	C01301	-3.83	3alpha,7alpha,12alpha-Trihydroxy-5beta-cholestan-26-al
475.311	C04554	-4.03	3alpha,7alpha-Dihydroxy-5beta-cholestanate; 3alpha,7alpha-Dihydroxy-5beta-cholestanoate
476.292	C00735	-2.82	Cortisol; Hydrocortisone; 11beta,17alpha,21-Trihydroxy-4-pregnene-3,20-dione; Kendall's compound F
476.308	C05454	-2.5	3alpha,7alpha,12alpha-Trihydroxy-5beta-cholestane; 5beta-Cholestane-3alpha,7alpha,12alpha-triol;
477.11	C00049	-2.02	L-Aspartate; L-Aspartic acid; 2-Aminosuccinic acid; L-Asp
477.274	C01301	-2.3	3alpha,7alpha,12alpha-Trihydroxy-5beta-cholestan-26-al
478.327	C00168	-1.91	Hydroxypyruvate; Hydroxypyruvic acid; 3-Hydroxypyruvate; 3-Hydroxypyruvic acid
479.33	C00762	-2.58	Cortisone; 17alpha,21-Dihydroxy-4-pregnene-3,11,20-trione; Kendall's compound E; Reichstein's substance Fa
480.231	C05452	-3.23	3alpha,7alpha-Dihydroxy-5beta-cholestane; 5beta-Cholestane-3alpha,7alpha-diol
480.278	C01190	-1.83	Glucosylceramide; Glucocerebroside; beta-D-Glucosyl-(1<->1)-ceramide; D-Glucosyl-N-acylsphingosine
481.15	C01051	-3.22	Uroporphyrinogen III
481.346	C05138	-2.96	17alpha-Hydroxypregnenolone
483.299	C03640	-2.16	Sphingosyl-phosphocholine
485.293	C02838	-3.15	L-Octanoylcarnitine
489.333	C01190	-1.97	Glucosylceramide; Glucocerebroside; beta-D-Glucosyl-(1<->1)-ceramide; D-Glucosyl-N-acylsphingosine
490.334	C01190	-1.72	Glucosylceramide; Glucocerebroside; beta-D-Glucosyl-(1<->1)-ceramide; D-Glucosyl-N-acylsphingosine
490.334	C03701	-2.09	Glycosyl-N-acylsphingosine
490.963	C00460	-1.95	dUTP; 2'-Deoxyuridine 5'-triphosphate
491.293	C05138	-1.79	17alpha-Hydroxypregnenolone
493.277	C01301	-3.1	3alpha,7alpha,12alpha-Trihydroxy-5beta-cholestan-26-al
495.307	C04707	-1.92	(5Z,13E)-11alpha-Hydroxy-9,15-dioxoprost-13-enoate; (5Z)-(15S)-11alpha-Hydroxy-9,15-dioxoprosta-13-enoate
497.259	C04707	-4.2	(5Z,13E)-11alpha-Hydroxy-9,15-dioxoprost-13-enoate; (5Z)-(15S)-11alpha-Hydroxy-9,15-dioxoprosta-13-enoate
501.363	C05452	-4.2	3alpha,7alpha-Dihydroxy-5beta-cholestane; 5beta-Cholestane-3alpha,7alpha-diol
502.367	C06425	-2.19	Icosanoic acid; Eicosanoic acid; Arachidic acid
503.235	C00169	-3.2	Carbamoyl phosphate
503.316	C05452	-2.24	3alpha,7alpha-Dihydroxy-5beta-cholestane; 5beta-Cholestane-3alpha,7alpha-diol
503.421	C00212	-2.89	Adenosine
506.308	C03640	-2.37	Sphingosyl-phosphocholine
506.308	C03640	-2.51	Sphingosyl-phosphocholine
506.308	C03640	-2.62	Sphingosyl-phosphocholine
507.05	C00167	-2.03	UDP-glucuronate; UDPglucuronate; UDP-D-glucuronate; UDP-alpha-D-glucuronate
507.329	C03640	-1.87	Sphingosyl-phosphocholine
508.332	C03640	-2.26	Sphingosyl-phosphocholine
509.329	C01301	-2.54	3alpha,7alpha,12alpha-Trihydroxy-5beta-cholestan-26-al
510.252	C05959	-2.72	11-epi-Prostaglandin F2alpha; 11-epi-Prostaglandin F2a; 11-epi-PGF2alpha; 11-epi-PGF2a
511.146	C00664	-2.7	5-Formiminotetrahydrofolate; 5-Formimidoyltetrahydrofolate
511.274	C04707	-2.74	(5Z,13E)-11alpha-Hydroxy-9,15-dioxoprost-13-enoate; (5Z)-(15S)-11alpha-Hydroxy-9,15-dioxoprosta-13-enoate
511.345	C01301	-4.33	3alpha,7alpha,12alpha-Trihydroxy-5beta-cholestan-26-al
513.316	C14772	-1.96	5,6-DHET; (8Z,11Z,14Z)-5,6-Dihydroxyeicosa-8,11,14-trienoic acid
515.298	C04707	-1.66	(5Z,13E)-11alpha-Hydroxy-9,15-dioxoprost-13-enoate; (5Z)-(15S)-11alpha-Hydroxy-9,15-dioxoprosta-13-enoate
517.03	C00295	-2.38	Orotate; Orotic acid; Uracil-6-carboxylic acid
517.237	C01301	-3.06	3alpha,7alpha,12alpha-Trihydroxy-5beta-cholestan-26-al
518.248	C05922	-2.46	Formamidopyrimidine nucleoside triphosphate
519.263	C02165	-2.06	Leukotriene B4; (6Z,8E,10E,14Z)-(5S,12R)-5,12-Dihydroxyeicosa-6,8,10,14-tetraenoate
524.257	C05452	-3.87	3alpha,7alpha-Dihydroxy-5beta-cholestane; 5beta-Cholestane-3alpha,7alpha-diol
524.303	C03640	-1.7	Sphingosyl-phosphocholine
524.932	C01024	-2.58	Hydroxymethylbilane
527.257	C04540	-2.15	N4-(Acetyl-beta-D-glucosaminyl)asparagine; N4-(beta-N-Acetyl-D-glucosaminyl)-L-asparagine
529.989	C00286	-2.79	dGTP; 2'-Deoxyguanosine 5'-triphosphate; Deoxyguanosine 5'-triphosphate; Deoxyguanosine triphosphate
530.972	C00081	-3.53	ITP; Inosine 5'-triphosphate; Inosine triphosphate; Inosine tripolyphosphate
533.41	C05454	-2.74	3alpha,7alpha,12alpha-Trihydroxy-5beta-cholestane; 5beta-Cholestane-3alpha,7alpha,12alpha-triol
533.966	C00049	-2.24	L-Aspartate; L-Aspartic acid; 2-Aminosuccinic acid; L-Asp
535.453	C06425	-2.23	Icosanoic acid; Eicosanoic acid; Arachidic acid
539.169	C00363	-3.14	dTDP; Deoxythymidine 5'-diphosphate
543.286	C04707	-2.56	(5Z,13E)-11alpha-Hydroxy-9,15-dioxoprost-13-enoate; (5Z)-(15S)-11alpha-Hydroxy-9,15-dioxoprosta-13-enoate
545.511	C00363	-1.69	dTDP; Deoxythymidine 5'-diphosphate
547.019	C05922	-2.09	Formamidopyrimidine nucleoside triphosphate
549.056	C00363	-2.11	dTDP; Deoxythymidine 5'-diphosphate
550.287	C01228	-2.2	Guanosine 3',5'-bis(diphosphate); Guanosine 3'-diphosphate 5'-diphosphate
551.015	C05922	-2.3	Formamidopyrimidine nucleoside triphosphate
551.355	C04707	-3.76	(5Z,13E)-11alpha-Hydroxy-9,15-dioxoprost-13-enoate; (5Z)-(15S)-11alpha-Hydroxy-9,15-dioxoprosta-13-enoate
552.274	C04707	-2.42	(5Z,13E)-11alpha-Hydroxy-9,15-dioxoprost-13-enoate; (5Z)-(15S)-11alpha-Hydroxy-9,15-dioxoprosta-13-enoate
552.358	C01301	-4.03	3alpha,7alpha,12alpha-Trihydroxy-5beta-cholestan-26-al
555.143	C01228	-1.99	Guanosine 3',5'-bis(diphosphate); Guanosine 3'-diphosphate 5'-diphosphate
556.07	C00049	-1.79	L-Aspartate; L-Aspartic acid; 2-Aminosuccinic acid; L-Asp
557.307	C01228	-2.58	Guanosine 3',5'-bis(diphosphate); Guanosine 3'-diphosphate 5'-diphosphate
558.378	C02166	-2.47	Leukotriene C4; LTC4
558.439	C03640	-2.26	Sphingosyl-phosphocholine
559.438	C04707	-2.42	(5Z,13E)-11alpha-Hydroxy-9,15-dioxoprost-13-enoate; (5Z)-(15S)-11alpha-Hydroxy-9,15-dioxoprosta-13-enoate
561.086	C00049	-3.13	L-Aspartate; L-Aspartic acid; 2-Aminosuccinic acid; L-Asp
561.935	C01228	-2.2	Guanosine 3',5'-bis(diphosphate); Guanosine 3'-diphosphate 5'-diphosphate
563.361	C03640	-2.13	Sphingosyl-phosphocholine
565.322	C01190	-1.71	Glucosylceramide; Glucocerebroside; beta-D-Glucosyl-(1<->1)-ceramide
566.02	C00049	-2.6	L-Aspartate; L-Aspartic acid; 2-Aminosuccinic acid; L-Asp
567.024	C00190	-1.96	UDP-D-xylose; UDP-xylose; UDP-alpha-D-xylose
569.087	C04392	-1.73	P1,P4-Bis(5'-xanthosyl) tetraphosphate; XppppX
569.316	C01301	-3.45	3alpha,7alpha,12alpha-Trihydroxy-5beta-cholestan-26-al

571.036	C00130	-2.18	IMP; Inosinic acid; Inosine monophosphate; Inosine 5'-monophosphate; Inosine 5'-phosphate; 5'-Inosinate
572.022	C00075	-2.95	UTP; Uridine 5'-triphosphate; Uridine triphosphate
575.009	C00190	-2.79	UDP-D-xylose; UDP-xylose; UDP-alpha-D-xylose
578.052	C05922	-1.91	Formamidopyrimidine nucleoside triphosphate
579.048	C05692	-2.1	Se-Adenosyl-L-selenohomocysteine; Se-Adenosylselenohomocysteine
579.96	C05922	-2.13	Formamidopyrimidine nucleoside triphosphate
580.999	C00300	-2.98	Creatine; alpha-Methylguanidino acetic acid; Methylglycocyamine
581.371	C06425	-2.1	Icosanoic acid; Eicosanoic acid; Arachidic acid
581.966	C05922	-3.17	Formamidopyrimidine nucleoside triphosphate
581.966	C05922	-3.19	Formamidopyrimidine nucleoside triphosphate
582.306	C01228	-2.35	Guanosine 3',5'-bis(diphosphate); Guanosine 3'-diphosphate 5'-diphosphate
586.025	C05922	-3.14	Formamidopyrimidine nucleoside triphosphate
587.965	C03680	-3.32	4-Imidazolone-5-propanoate; 4-Imidazolone-5-propionic acid; 4,5-Dihydro-4-oxo-5-imidazolepropanoate
589.036	C00075	-2.13	UTP; Uridine 5'-triphosphate; Uridine triphosphate
591.256	C00363	-3.39	dTDP; Deoxythymidine 5'-diphosphate
597.262	C03263	-2.09	Coproporphyrinogen III
601.463	C04707	-2.09	(5Z,13E)-11alpha-Hydroxy-9,15-dioxoprost-13-enoate; (5Z)-(15S)-11alpha-Hydroxy-9,15-dioxoprosta-13-enoate
602.466	C04707	-2.5	(5Z,13E)-11alpha-Hydroxy-9,15-dioxoprost-13-enoate; (5Z)-(15S)-11alpha-Hydroxy-9,15-dioxoprosta-13-enoate
609.264	C16641	-3.14	Irinotecan
609.272	C02166	-1.87	Leukotriene C4; LTC4
613.054	C01051	-1.89	Uroporphyrinogen III
614.184	C00075	-2.2	UTP; Uridine 5'-triphosphate; Uridine triphosphate
616.302	C05922	-1.99	Formamidopyrimidine nucleoside triphosphate
618.993	C00167	-1.68	UDP-glucuronate; UDPglucuronate; UDP-D-glucuronate; UDP-alpha-D-glucuronate
619.057	C08565	-2.01	Portulacaxanthin II
619.136	C02166	-1.75	Leukotriene C4; LTC4
620.353	C00042	-2.12	Succinate; Succinic acid; Butanedionic acid; Ethylenesuccinic acid
621.34	C03640	-2.79	Sphingosyl-phosphocholine
622.344	C01079	-2.15	Protoporphyrinogen IX
623.271	C16641	-2.72	Irinotecan
623.301	C00167	-1.71	UDP-glucuronate; UDPglucuronate; UDP-D-glucuronate; UDP-alpha-D-glucuronate
629.799	C03263	-2.62	Coproporphyrinogen III
633.267	C03263	-3.24	Coproporphyrinogen III
636.274	C16641	-2.5	Irinotecan
641.017	C00075	-3.18	UTP; Uridine 5'-triphosphate; Uridine triphosphate
641.254	C00486	-2.13	Bilirubin
641.274	C03263	-2.96	Coproporphyrinogen III
641.922	C01228	-2.01	Guanosine 3',5'-bis(diphosphate); Guanosine 3'-diphosphate 5'-diphosphate
643.245	C00239	-1.76	dCMP; Deoxycytidylic acid; Deoxycytidine monophosphate; Deoxycytidylate; 2'-Deoxycytidine 5'-monophosphate
643.264	C03263	-2.45	Coproporphyrinogen III
645.201	C16641	-1.84	Irinotecan
645.417	C02990	-3.66	L-Palmitoylcarnitine
649.096	C01024	-1.71	Hydroxymethylbilane
650.235	C01051	-2.87	Uroporphyrinogen III
651.257	C00167	-2.01	UDP-glucuronate; UDPglucuronate; UDP-D-glucuronate; UDP-alpha-D-glucuronate
652.247	C04886	-1.75	alpha-N-Acetylneuraminyl-2,6-beta-D-galactosyl-1,4-N-acetyl-beta-D-glucosamine
652.773	C04856	-2.69	(6S)-6beta-Hydroxy-1,4,5,6-tetrahydronicotinamide-adenine dinucleotide; (S)-NADH-hydrate
654.303	C03263	-2.87	Coproporphyrinogen III
655.237	C03263	-3.29	Coproporphyrinogen III
656.249	C03263	-2.61	Coproporphyrinogen III
658.238	C01051	-2.28	Uroporphyrinogen III
658.245	C03263	-1.71	Coproporphyrinogen III
659.235	C01051	-2.73	Uroporphyrinogen III
661.271	C09332	-1.82	THF-L-glutamate; Tetrahydropteroyldiglutamate; (6S)-H4PteGlu2
661.321	C03263	-2.51	Coproporphyrinogen III
661.321	C05768	-2.03	Coproporphyrinogen I
662.272	C03263	-2.42	Coproporphyrinogen III
666.259	C04392	-2.94	P1,P4-Bis(5'-xanthosyl) tetraphosphate; XppppX
666.277	C01051	-2.29	Uroporphyrinogen III
667.198	C05922	-2.26	Formamidopyrimidine nucleoside triphosphate
668.277	C06198	-2.45	P1,P4-Bis(5'-uridyl) tetraphosphate; UppppU
669.229	C00049	-1.7	L-Aspartate; L-Aspartic acid; 2-Aminosuccinic acid; L-Asp
669.293	C03263	-2.49	Coproporphyrinogen III
670.289	C01051	-2.43	Uroporphyrinogen III
673.244	C04886	-1.73	alpha-N-Acetylneuraminyl-2,6-beta-D-galactosyl-1,4-N-acetyl-beta-D-glucosamine
673.364	C04707	-1.72	(5Z,13E)-11alpha-Hydroxy-9,15-dioxoprost-13-enoate; (5Z)-(15S)-11alpha-Hydroxy-9,15-dioxoprosta-13-enoate
674.243	C03263	-3.4	Coproporphyrinogen III
674.255	C03263	-2.23	Coproporphyrinogen III
675.224	C03263	-2	Coproporphyrinogen III
676.225	C04856	-2.67	(6S)-6beta-Hydroxy-1,4,5,6-tetrahydronicotinamide-adenine dinucleotide; (S)-NADH-hydrate; (S)-NADHX
676.297	C03263	-1.95	Coproporphyrinogen III
677.225	C03263	-3.1	Coproporphyrinogen III
677.249	C03263	-1.85	Coproporphyrinogen III
677.278	C03263	-2.95	Coproporphyrinogen III
678.252	C06198	-3.35	P1,P4-Bis(5'-uridyl) tetraphosphate; UppppU
683.192	C00049	-1.96	L-Aspartate; L-Aspartic acid; 2-Aminosuccinic acid; L-Asp
684.282	C03263	-2.89	Coproporphyrinogen III
687.252	C03263	-1.76	Coproporphyrinogen III
687.375	C01079	-2.05	Protoporphyrinogen IX
688.285	C03263	-3.1	Coproporphyrinogen III
689.253	C03263	-1.69	Coproporphyrinogen III
690.26	C03263	-2.54	Coproporphyrinogen III
691.276	C00337	-2.45	(S)-Dihydroorotate; (S)-4,5-Dihydroorotate; L-Dihydroorotate; L-Dihydroorotic acid; Dihydro-L-orotic acid

691.76	C01944	-2.05	Octanoyl-CoA
692.157	C04540	-1.98	N4-(Acetyl-beta-D-glucosaminyl)asparagine; N4-(beta-N-Acetyl-D-glucosaminyl)-L-asparagine
692.225	C01051	-2.5	Uroporphyrinogen III
693.381	C03263	-1.74	Coproporphyrinogen III
693.631	C01352	-2.13	FADH2
694.261	C03263	-1.91	Coproporphyrinogen III
695.264	C03263	-1.94	Coproporphyrinogen III
695.338	C03263	-1.93	Coproporphyrinogen III
696.335	C03263	-2.1	Coproporphyrinogen III
697.269	C03263	-3.57	Coproporphyrinogen III
699.278	C03263	-3.02	Coproporphyrinogen III
699.278	C05768	-2.78	Coproporphyrinogen I
700.269	C03263	-1.89	Coproporphyrinogen III
701.235	C03263	-2.51	Coproporphyrinogen III
704.356	C00075	-1.98	UTP; Uridine 5'-triphosphate; Uridine triphosphate
705.235	C03263	-1.82	Coproporphyrinogen III
705.321	C03263	-3.72	Coproporphyrinogen III
705.349	C01228	-3.89	Guanosine 3',5'-bis(diphosphate); Guanosine 3'-diphosphate 5'-diphosphate
706.235	C03263	-2.66	Coproporphyrinogen III
706.326	C03263	-2.52	Coproporphyrinogen III
707.251	C03263	-2.48	Coproporphyrinogen III
709.22	C03263	-2.28	Coproporphyrinogen III
709.245	C00167	-2.09	UDP-glucuronate; UDPglucuronate; UDP-D-glucuronate; UDP-alpha-D-glucuronate
709.263	C17234	-1.93	2-Aminobut-2-enoate; (2Z)-2-Aminobut-2-enoic acid; 2-Ammoniobut-2-enoate
709.394	C01228	-1.69	Guanosine 3',5'-bis(diphosphate); Guanosine 3'-diphosphate 5'-diphosphate
710.142	C02305	-1.9	Phosphocreatine; N-Phosphocreatine; Creatine phosphate
710.251	C03263	-2.16	Coproporphyrinogen III
711.231	C04392	-2.02	P1,P4-Bis(5'-xanthosyl) tetraphosphate; XppppX
711.247	C04392	-2.88	P1,P4-Bis(5'-xanthosyl) tetraphosphate; XppppX
712.312	C03263	-3.62	Coproporphyrinogen III
713.255	C04392	-2.39	P1,P4-Bis(5'-xanthosyl) tetraphosphate; XppppX
713.31	C03263	-3.49	Coproporphyrinogen III
714.302	C03263	-2.82	Coproporphyrinogen III
718.196	C00100	-2.85	Propanoyl-CoA; Propionyl-CoA; Propionyl coenzyme A
718.279	C00379	-1.85	Xylitol
720.291	C00049	-1.98	L-Aspartate; L-Aspartic acid; 2-Aminosuccinic acid; L-Asp
721.325	C05922	-1.81	Formamidopyrimidine nucleoside triphosphate
721.396	C04540	-2.18	N4-(Acetyl-beta-D-glucosaminyl)asparagine; N4-(beta-N-Acetyl-D-glucosaminyl)-L-asparagine
724.25	C01051	-2.14	Uroporphyrinogen III
724.319	C03263	-2.97	Coproporphyrinogen III
725.233	C16470	-1.73	5-Methylhex-4-enoyl-CoA
725.997	C00049	-2.06	L-Aspartate; L-Aspartic acid; 2-Aminosuccinic acid; L-Asp
726.315	C01228	-2.12	Guanosine 3',5'-bis(diphosphate); Guanosine 3'-diphosphate 5'-diphosphate
727.383	C05922	-1.75	Formamidopyrimidine nucleoside triphosphate
728.223	C01051	-1.74	Uroporphyrinogen III
729.234	C04392	-2.47	P1,P4-Bis(5'-xanthosyl) tetraphosphate; XppppX
729.291	C06198	-1.86	P1,P4-Bis(5'-uridyl) tetraphosphate; UppppU
729.337	C00190	-2.58	UDP-D-xylose; UDP-xylose; UDP-alpha-D-xylose
730.229	C01051	-2.84	Uroporphyrinogen III
730.29	C01051	-2.28	Uroporphyrinogen III
730.368	C00190	-2.64	UDP-D-xylose; UDP-xylose; UDP-alpha-D-xylose
732.286	C00882	-2.54	Dephospho-CoA; Dephosphocoenzyme A; 3'-Dephospho-CoA
733.164	C00190	-2.21	UDP-D-xylose; UDP-xylose; UDP-alpha-D-xylose
734.313	C01024	-2.62	Hydroxymethylbilane
735.267	C04886	-1.68	alpha-N-Acetylneuraminyl-2,6-beta-D-galactosyl-1,4-N-acetyl-beta-D-glucosamine
737.277	C00049	-3.26	L-Aspartate; L-Aspartic acid; 2-Aminosuccinic acid; L-Asp
737.628	C04899	-2.49	(6S)-6beta-Hydroxy-1,4,5,6-tetrahydronicotinamide-adenine dinucleotide phosphate; (S)-NADPH-hydrate
738.323	C03263	-2.04	Coproporphyrinogen III
738.365	C03263	-2.71	Coproporphyrinogen III
739.312	C03263	-3.06	Coproporphyrinogen III
740.831	C00547	-1.83	L-Noradrenaline; Noradrenaline; Norepinephrine; Arterenol; 4-[(1R)-2-Amino-1-hydroxyethyl]-1,2-benzenediol
742.22	C00100	-2.23	Propanoyl-CoA; Propionyl-CoA; Propionyl coenzyme A
743.251	C06198	-2.26	P1,P4-Bis(5'-uridyl) tetraphosphate; UppppU
743.995	C05922	-2.36	Formamidopyrimidine nucleoside triphosphate
744.257	C03263	-2.43	Coproporphyrinogen III
745.219	C01051	-2.47	Uroporphyrinogen III
745.26	C03263	-1.95	Coproporphyrinogen III
745.306	C03263	-3.4	Coproporphyrinogen III
746.213	C01051	-2.25	Uroporphyrinogen III
747.21	C01051	-2.57	Uroporphyrinogen III
749.011	C05922	-2.3	Formamidopyrimidine nucleoside triphosphate
749.179	C01051	-3.02	Uroporphyrinogen III
749.203	C04392	-2.13	P1,P4-Bis(5'-xanthosyl) tetraphosphate; XppppX
749.31	C03263	-3.35	Coproporphyrinogen III
751.185	C01260	-2.16	P1,P4-Bis(5'-adenosyl)tetraphosphate; ApppppA
752.108	C16470	-2.39	5-Methylhex-4-enoyl-CoA
753.252	C01944	-2.35	Octanoyl-CoA
754.26	C01944	-1.74	Octanoyl-CoA
755.341	C01228	-2.06	Guanosine 3',5'-bis(diphosphate); Guanosine 3'-diphosphate 5'-diphosphate
756.302	C01024	-1.82	Hydroxymethylbilane
756.338	C06198	-2.99	P1,P4-Bis(5'-uridyl) tetraphosphate; UppppU
757.349	C01228	-1.99	Guanosine 3',5'-bis(diphosphate); Guanosine 3'-diphosphate 5'-diphosphate
758.295	C01024	-2	Hydroxymethylbilane

759.244	C01051	-1.84	Uroporphyrinogen III
760.232	C04899	-1.91	(6S)-6beta-Hydroxy-1,4,5,6-tetrahydronicotinamide-adenine dinucleotide phosphate; (S)-NADPH-hydrate
761.219	C01217	-1.71	5,6,7,8-Tetrahydromethanopterin; H4MPT; THMPT; Tetrahydromethanopterin
761.246	C01051	-2.28	Uroporphyrinogen III
761.283	C01024	-1.66	Hydroxymethylbilane
761.306	C06198	-2.98	P1,P4-Bis(5'-uridyl) tetraphosphate; UppppU
762.224	C01051	-1.91	Uroporphyrinogen III
762.269	C06198	-1.94	P1,P4-Bis(5'-uridyl) tetraphosphate; UppppU
764.25	C16470	-2.35	5-Methylhex-4-enoyl-CoA
765.276	C01051	-2.45	Uroporphyrinogen III
766.182	C00882	-1.91	Dephospho-CoA; Dephosphocoenzyme A; 3'-Dephospho-CoA
766.196	C00024	-2.09	Acetyl-CoA; Acetyl coenzyme A
766.238	C01024	-1.84	Hydroxymethylbilane
766.288	C06198	-2.59	P1,P4-Bis(5'-uridyl) tetraphosphate; UppppU
767.191	C06198	-1.75	P1,P4-Bis(5'-uridyl) tetraphosphate; UppppU
767.262	C06198	-2.33	P1,P4-Bis(5'-uridyl) tetraphosphate; UppppU
767.706	C01260	-1.82	P1,P4-Bis(5'-adenosyl)tetraphosphate; ApppppA
769.177	C00100	-2.74	Propanoyl-CoA; Propionyl-CoA; Propionyl coenzyme A
770.183	C01352	-1.92	FADH2
771.182	C01352	-3.56	FADH2
776.431	C00500	-3.02	Biliverdin; Biliverdin IX alpha
777.266	C04392	-2.44	P1,P4-Bis(5'-xanthosyl) tetraphosphate; XppppX
778.287	C06198	-2.01	P1,P4-Bis(5'-uridyl) tetraphosphate; UppppU
778.305	C06198	-1.83	P1,P4-Bis(5'-uridyl) tetraphosphate; UppppU
779.304	C06198	-3.15	P1,P4-Bis(5'-uridyl) tetraphosphate; UppppU
780.171	C00024	-2.94	Acetyl-CoA; Acetyl coenzyme A
780.256	C16470	-2.25	5-Methylhex-4-enoyl-CoA
780.338	C01051	-2.89	Uroporphyrinogen III
781.288	C01217	-2.05	5,6,7,8-Tetrahydromethanopterin; H4MPT; THMPT; Tetrahydromethanopterin
781.329	C01051	-3.13	Uroporphyrinogen III
782.376	C04377	-1.81	5,10-Methylenetetrahydromethanopterin; N5,N10-Methylenetetrahydromethanopterin
783.263	C01352	-2.68	FADH2
783.275	C00049	-2.04	L-Aspartate; L-Aspartic acid; 2-Aminosuccinic acid; L-Asp
784.418	C04377	-1.75	5,10-Methylenetetrahydromethanopterin; N5,N10-Methylenetetrahydromethanopterin
786.135	C00016	-3.92	FAD; Flavin adenine dinucleotide
787.248	C01051	-3.2	Uroporphyrinogen III
788.185	C00100	-1.67	Propanoyl-CoA; Propionyl-CoA; Propionyl coenzyme A
788.237	C01352	-2.27	FADH2
789.178	C01352	-1.92	FADH2
789.206	C01352	-3.58	FADH2
789.271	C06198	-2.26	P1,P4-Bis(5'-uridyl) tetraphosphate; UppppU
790.183	C01352	-3.03	FADH2
790.269	C06198	-2.31	P1,P4-Bis(5'-uridyl) tetraphosphate; UppppU
790.313	C01217	-1.74	5,6,7,8-Tetrahydromethanopterin; H4MPT; THMPT; Tetrahydromethanopterin
791.211	C00024	-3.3	Acetyl-CoA; Acetyl coenzyme A
791.267	C01051	-1.85	Uroporphyrinogen III
791.289	C06198	-3.57	P1,P4-Bis(5'-uridyl) tetraphosphate; UppppU
791.413	C00097	-1.89	L-Cysteine; L-2-Amino-3-mercaptopropionic acid
792.288	C06198	-1.73	P1,P4-Bis(5'-uridyl) tetraphosphate; UppppU
794.272	C06198	-2.56	P1,P4-Bis(5'-uridyl) tetraphosphate; UppppU
795.308	C11134	-2.18	Testosterone glucuronide; Testosterone 17beta-(beta-D-glucuronide)
796.16	C00024	-2.57	Acetyl-CoA; Acetyl coenzyme A
796.311	C01051	-2.2	Uroporphyrinogen III
797.194	C00882	-2.07	Dephospho-CoA; Dephosphocoenzyme A; 3'-Dephospho-CoA
797.237	C01944	-3.05	Octanoyl-CoA
798.18	C01352	-1.81	FADH2
798.195	C00024	-2.21	Acetyl-CoA; Acetyl coenzyme A
798.235	C01217	-1.87	5,6,7,8-Tetrahydromethanopterin; H4MPT; THMPT; Tetrahydromethanopterin
798.303	C01051	-1.71	Uroporphyrinogen III
799.204	C00024	-3.29	Acetyl-CoA; Acetyl coenzyme A
799.248	C01944	-2.53	Octanoyl-CoA
800.197	C04405	-1.7	(2S,3S)-3-Hydroxy-2-methylbutanoyl-CoA; (S)-3-Hydroxy-2-methylbutyryl-CoA
800.258	C01944	-1.81	Octanoyl-CoA
800.314	C04392	-1.97	P1,P4-Bis(5'-xanthosyl) tetraphosphate; XppppX
801.067	C04899	-2.57	(6S)-6beta-Hydroxy-1,4,5,6-tetrahydronicotinamide-adenine dinucleotide phosphate; (S)-NADPH-hydrate
801.259	C06198	-3.9	P1,P4-Bis(5'-uridyl) tetraphosphate; UppppU
801.318	C06198	-2.8	P1,P4-Bis(5'-uridyl) tetraphosphate; UppppU
802.266	C04392	-2.24	P1,P4-Bis(5'-xanthosyl) tetraphosphate; XppppX
802.324	C06198	-4.24	P1,P4-Bis(5'-uridyl) tetraphosphate; UppppU
803.26	C01944	-2.34	Octanoyl-CoA
803.32	C01024	-2.67	Hydroxymethylbilane
804.258	C16470	-1.75	5-Methylhex-4-enoyl-CoA
806.258	C00363	-2.05	dTDP; Deoxythymidine 5'-diphosphate
807.071	C00016	-3.78	FAD; Flavin adenine dinucleotide
808.081	C00016	-3.39	FAD; Flavin adenine dinucleotide
808.384	C00956	-2.11	L-2-Aminoadipate; L-alpha-Aminoadipate; L-alpha-Aminoadipic acid; L-2-Aminoadipic acid
810.43	C00882	-2.07	Dephospho-CoA; Dephosphocoenzyme A; 3'-Dephospho-CoA
811.094	C00016	-2.35	FAD; Flavin adenine dinucleotide
811.186	C00100	-2.65	Propanoyl-CoA; Propionyl-CoA; Propionyl coenzyme A
811.292	C01944	-2.74	Octanoyl-CoA
812.178	C00100	-2.41	Propanoyl-CoA; Propionyl-CoA; Propionyl coenzyme A
812.198	C00083	-2.21	Malonyl-CoA; Malonyl coenzyme A
812.293	C06198	-2.55	P1,P4-Bis(5'-uridyl) tetraphosphate; UppppU

812.983	C01024	-2.99	Hydroxymethylbilane
813.203	C00024	-1.69	Acetyl-CoA; Acetyl coenzyme A
813.272	C06198	-2.3	P1,P4-Bis(5'-uridyl) tetraphosphate; UppppU
813.969	C06198	-2.52	P1,P4-Bis(5'-uridyl) tetraphosphate; UppppU
814.179	C00100	-2.85	Propanoyl-CoA; Propionyl-CoA; Propionyl coenzyme A
814.199	C01352	-1.66	FADH2
814.971	C01024	-2.35	Hydroxymethylbilane
814.988	C01217	-2.69	5,6,7,8-Tetrahydromethanopterin; H4MPT; THMPT; Tetrahydromethanopterin
815.231	C01944	-2.73	Octanoyl-CoA
816.18	C00100	-2.59	Propanoyl-CoA; Propionyl-CoA; Propionyl coenzyme A
818.317	C04377	-1.7	5,10-Methylenetetrahydromethanopterin; N5,N10-Methylenetetrahydromethanopterin
820.324	C01051	-1.93	Uroporphyrinogen III
821.326	C06198	-2.33	P1,P4-Bis(5'-uridyl) tetraphosphate; UppppU
823.32	C01051	-1.89	Uroporphyrinogen III
824.045	C00016	-3.69	FAD; Flavin adenine dinucleotide
824.122	C00016	-2.41	FAD; Flavin adenine dinucleotide
824.361	C06198	-2.38	P1,P4-Bis(5'-uridyl) tetraphosphate; UppppU
824.455	C05922	-2.08	Formamidopyrimidine nucleoside triphosphate
825.054	C00083	-2.58	Malonyl-CoA; Malonyl coenzyme A
825.357	C06198	-2.92	P1,P4-Bis(5'-uridyl) tetraphosphate; UppppU
826.055	C00016	-3.3	FAD; Flavin adenine dinucleotide
826.121	C00016	-4.6	FAD; Flavin adenine dinucleotide
826.121	C00016	-4.73	FAD; Flavin adenine dinucleotide
827.052	C00016	-2.64	FAD; Flavin adenine dinucleotide
827.124	C00016	-1.99	FAD; Flavin adenine dinucleotide
827.248	C16470	-2.3	5-Methylhex-4-enoyl-CoA
828.06	C00016	-3.26	FAD; Flavin adenine dinucleotide
828.367	C01051	-2.19	Uroporphyrinogen III
829.246	C01944	-2.52	Octanoyl-CoA
830.226	C04377	-2.01	5,10-Methylenetetrahydromethanopterin; N5,N10-Methylenetetrahydromethanopterin
830.252	C04405	-1.98	(2S,3S)-3-Hydroxy-2-methylbutanoyl-CoA; (S)-3-Hydroxy-2-methylbutyryl-CoA
832.386	C06198	-2.95	P1,P4-Bis(5'-uridyl) tetraphosphate; UppppU
833.229	C00091	-2.86	Succinyl-CoA; Succinyl coenzyme A
833.322	C01051	-1.75	Uroporphyrinogen III
834.231	C01944	-3.22	Octanoyl-CoA
834.397	C04377	-1.67	5,10-Methylenetetrahydromethanopterin; N5,N10-Methylenetetrahydromethanopterin
837.134	C00024	-3.15	Acetyl-CoA; Acetyl coenzyme A
837.274	C01051	-2.16	Uroporphyrinogen III
839.066	C00024	-2.82	Acetyl-CoA; Acetyl coenzyme A
841.05	C00294	-3.49	Inosine
841.156	C00136	-1.89	Butanoyl-CoA; Butyryl-CoA
842.056	C01352	-1.68	FADH2
845.393	C01051	-1.92	Uroporphyrinogen III
845.413	C03263	-3.16	Coproporphyrinogen III
846.416	C01024	-2.21	Hydroxymethylbilane
847.266	C04392	-2.13	P1,P4-Bis(5'-xanthosyl) tetraphosphate; XppppX
848.093	C00024	-2.14	Acetyl-CoA; Acetyl coenzyme A
849.097	C00024	-2.26	Acetyl-CoA; Acetyl coenzyme A
849.352	C04392	-3.06	P1,P4-Bis(5'-xanthosyl) tetraphosphate; XppppX
850.107	C00024	-2.4	Acetyl-CoA; Acetyl coenzyme A
850.22	C04405	-2.98	(2S,3S)-3-Hydroxy-2-methylbutanoyl-CoA; (S)-3-Hydroxy-2-methylbutyryl-CoA
852.411	C04392	-2.12	P1,P4-Bis(5'-xanthosyl) tetraphosphate; XppppX
853.254	C04405	-1.89	(2S,3S)-3-Hydroxy-2-methylbutanoyl-CoA; (S)-3-Hydroxy-2-methylbutyryl-CoA
853.288	C01024	-1.86	Hydroxymethylbilane
853.361	C01944	-1.94	Octanoyl-CoA
853.384	C04392	-2.47	P1,P4-Bis(5'-xanthosyl) tetraphosphate; XppppX
854.124	C00083	-2.02	Malonyl-CoA; Malonyl coenzyme A
854.159	C01144	-2.23	(S)-3-Hydroxybutanoyl-CoA; (S)-3-Hydroxybutyryl-CoA
854.159	C04047	-2.61	3-Hydroxy-2-methylpropanoyl-CoA; 3-Hydroxy-2-methylpropionyl-CoA; 3-Hydroxyisobutyryl-CoA
854.159	C05116	-2.65	3-Hydroxybutanoyl-CoA; 3-Hydroxybutyryl-CoA
854.159	C06000	-2.9	(S)-3-Hydroxyisobutyryl-CoA
854.34	C04392	-2.45	P1,P4-Bis(5'-xanthosyl) tetraphosphate; XppppX
854.383	C01051	-2.53	Uroporphyrinogen III
859.267	C01051	-1.66	Uroporphyrinogen III
861.201	C01051	-3.27	Uroporphyrinogen III
861.279	C01051	-1.78	Uroporphyrinogen III
861.362	C06198	-2.53	P1,P4-Bis(5'-uridyl) tetraphosphate; UppppU
862.28	C04392	-2.99	P1,P4-Bis(5'-xanthosyl) tetraphosphate; XppppX
862.349	C06198	-2.44	P1,P4-Bis(5'-uridyl) tetraphosphate; UppppU
862.372	C01051	-1.95	Uroporphyrinogen III
862.412	C01051	-2.2	Uroporphyrinogen III
862.518	C01024	-2.07	Hydroxymethylbilane
863.204	C01051	-2.99	Uroporphyrinogen III
863.348	C01051	-2.08	Uroporphyrinogen III
863.409	C01024	-2.3	Hydroxymethylbilane
863.52	C01024	-2.4	Hydroxymethylbilane
864.204	C16470	-2.4	5-Methylhex-4-enoyl-CoA
864.377	C01051	-1.82	Uroporphyrinogen III
864.399	C00016	-2.39	FAD; Flavin adenine dinucleotide
864.421	C06198	-2.44	P1,P4-Bis(5'-uridyl) tetraphosphate; UppppU
864.521	C01024	-1.77	Hydroxymethylbilane
865.31	C01024	-2.39	Hydroxymethylbilane
865.379	C00049	-1.86	L-Aspartate; L-Aspartic acid; 2-Aminosuccinic acid; L-Asp

866.204	C01024	-1.74	Hydroxymethylbilane
866.314	C04392	-2.29	P1,P4-Bis(5'-xanthosyl) tetraphosphate; XppppX
866.381	C06198	-4.17	P1,P4-Bis(5'-uridyl) tetraphosphate; UppppU
867.204	C01024	-1.94	Hydroxymethylbilane
867.339	C01051	-1.73	Uroporphyrinogen III
867.542	C16470	-2.32	5-Methylhex-4-enoyl-CoA
868.172	C04405	-3.16	(2S,3S)-3-Hydroxy-2-methylbutanoyl-CoA; (S)-3-Hydroxy-2-methylbutyryl-CoA
868.172	C05271	-2.82	trans-Hex-2-enoyl-CoA; (2E)-Hexenoyl-CoA
868.172	C05998	-2.94	3-Hydroxyisovaleryl-CoA; 3-Hydroxyisovaleryl coenzyme A
868.212	C04405	-2.32	(2S,3S)-3-Hydroxy-2-methylbutanoyl-CoA; (S)-3-Hydroxy-2-methylbutyryl-CoA
868.229	C01944	-1.93	Octanoyl-CoA
868.291	C01024	-2.22	Hydroxymethylbilane
868.546	C16471	-2.94	5-Methyl-3-oxo-4-hexenoyl-CoA
869.552	C16471	-3.75	5-Methyl-3-oxo-4-hexenoyl-CoA
870.342	C01051	-2.8	Uroporphyrinogen III
872.088	C00024	-1.66	Acetyl-CoA; Acetyl coenzyme A
872.315	C01024	-2.14	Hydroxymethylbilane
873.407	C01051	-2.46	Uroporphyrinogen III
874.416	C06198	-2.88	P1,P4-Bis(5'-uridyl) tetraphosphate; UppppU
875.34	C01944	-1.84	Octanoyl-CoA
876.183	C16470	-2.32	5-Methylhex-4-enoyl-CoA
876.233	C04405	-1.69	(2S,3S)-3-Hydroxy-2-methylbutanoyl-CoA; (S)-3-Hydroxy-2-methylbutyryl-CoA
877.239	C01944	-2.36	Octanoyl-CoA
877.273	C01024	-1.87	Hydroxymethylbilane
877.329	C04392	-2.17	P1,P4-Bis(5'-xanthosyl) tetraphosphate; XppppX
877.414	C06198	-2.43	P1,P4-Bis(5'-uridyl) tetraphosphate; UppppU
878.269	C01024	-2	Hydroxymethylbilane
878.336	C04392	-2.74	P1,P4-Bis(5'-xanthosyl) tetraphosphate; XppppX
879.189	C05271	-2.32	trans-Hex-2-enoyl-CoA; (2E)-Hexenoyl-CoA
879.189	C16470	-2.51	5-Methylhex-4-enoyl-CoA
880.35	C01051	-2.53	Uroporphyrinogen III
881.209	C01024	-2.31	Hydroxymethylbilane
882.097	C00640	-1.8	(3S)-3-Hydroxyacyl-CoA; (S)-3-Hydroxyacyl-CoA
882.097	C00640	-1.94	(3S)-3-Hydroxyacyl-CoA; (S)-3-Hydroxyacyl-CoA
882.097	C01086	-1.66	(3R)-3-Hydroxyacyl-CoA; (R)-3-Hydroxyacyl-CoA
882.097	C01086	-1.76	(3R)-3-Hydroxyacyl-CoA; (R)-3-Hydroxyacyl-CoA
882.097	C05668	-2.71	3-Hydroxypropionyl-CoA; 3-Hydroxypropionyl coenzyme A; 3-Hydroxypropanoyl-CoA
882.097	C05668	-2.77	3-Hydroxypropionyl-CoA; 3-Hydroxypropionyl coenzyme A; 3-Hydroxypropanoyl-CoA
882.162	C16470	-2.64	5-Methylhex-4-enoyl-CoA
882.204	C01944	-2.72	Octanoyl-CoA
882.425	C01051	-2.26	Uroporphyrinogen III
883.319	C01024	-1.67	Hydroxymethylbilane
884.213	C01051	-1.77	Uroporphyrinogen III
885.185	C05271	-2.07	trans-Hex-2-enoyl-CoA; (2E)-Hexenoyl-CoA
887.565	C01217	-2.18	5,6,7,8-Tetrahydromethanopterin; H4MPT; THMPT; Tetrahydromethanopterin
888.101	C01352	-1.69	FADH2
888.158	C05271	-1.72	trans-Hex-2-enoyl-CoA; (2E)-Hexenoyl-CoA
888.31	C04392	-2.96	P1,P4-Bis(5'-xanthosyl) tetraphosphate; XppppX
889.136	C00136	-3.15	Butanoyl-CoA; Butyryl-CoA
889.136	C03344	-2.76	2-Methylacetoacetyl-CoA; 2-Methyl-3-acetoacetyl-CoA
889.181	C04405	-1.66	(2S,3S)-3-Hydroxy-2-methylbutanoyl-CoA; (S)-3-Hydroxy-2-methylbutyryl-CoA
889.532	C01024	-3.47	Hydroxymethylbilane
890.326	C01944	-1.98	Octanoyl-CoA
891.119	C02557	-1.74	Methylmalonyl-CoA; Methylmalonyl coenzyme A; 2-Methylmalonyl-CoA
891.119	C02557	-1.88	Methylmalonyl-CoA; Methylmalonyl coenzyme A; 2-Methylmalonyl-CoA
891.119	C03069	-1.66	3-Methylcrotonyl-CoA; 3-Methylbut-2-enoyl-CoA; 3-Methylcrotonoyl-CoA; Dimethylacryloyl-CoA
891.119	C03069	-1.82	3-Methylcrotonyl-CoA; 3-Methylbut-2-enoyl-CoA; 3-Methylcrotonoyl-CoA; Dimethylacryloyl-CoA
891.119	C03069	-1.9	3-Methylcrotonyl-CoA; 3-Methylbut-2-enoyl-CoA; 3-Methylcrotonoyl-CoA; Dimethylacryloyl-CoA
891.119	C03345	-2.08	2-Methylbut-2-enoyl-CoA; trans-2-Methylbut-2-enoyl-CoA; Tiglyl-CoA; (E)-2-Methylcrotonoyl-CoA;
891.119	C03345	-2.25	2-Methylbut-2-enoyl-CoA; trans-2-Methylbut-2-enoyl-CoA; Tiglyl-CoA; (E)-2-Methylcrotonoyl-CoA;
891.119	C03345	-2.26	2-Methylbut-2-enoyl-CoA; trans-2-Methylbut-2-enoyl-CoA; Tiglyl-CoA; (E)-2-Methylcrotonoyl-CoA;
891.119	C16470	-1.88	5-Methylhex-4-enoyl-CoA
891.131	C16471	-1.65	5-Methyl-3-oxo-4-hexenoyl-CoA
891.319	C04392	-2.69	P1,P4-Bis(5'-xanthosyl) tetraphosphate; XppppX
891.389	C00049	-1.77	L-Aspartate; L-Aspartic acid; 2-Aminosuccinic acid; L-Asp
892.179	C00136	-1.74	Butanoyl-CoA; Butyryl-CoA
892.179	C16471	-1.84	5-Methyl-3-oxo-4-hexenoyl-CoA
892.247	C04405	-1.74	(2S,3S)-3-Hydroxy-2-methylbutanoyl-CoA; (S)-3-Hydroxy-2-methylbutyryl-CoA
892.311	C04392	-2.24	P1,P4-Bis(5'-xanthosyl) tetraphosphate; XppppX
892.392	C04377	-1.96	5,10-Methylenetetrahydromethanopterin; N5,N10-Methylenetetrahydromethanopterin
892.992	C04392	-1.89	P1,P4-Bis(5'-xanthosyl) tetraphosphate; XppppX
893.139	C04405	-1.7	(2S,3S)-3-Hydroxy-2-methylbutanoyl-CoA; (S)-3-Hydroxy-2-methylbutyryl-CoA
893.303	C04392	-3.3	P1,P4-Bis(5'-xanthosyl) tetraphosphate; XppppX
893.394	C06198	-2.78	P1,P4-Bis(5'-uridyl) tetraphosphate; UppppU
893.558	C01944	-3.7	Octanoyl-CoA
894.228	C04405	-1.84	(2S,3S)-3-Hydroxy-2-methylbutanoyl-CoA; (S)-3-Hydroxy-2-methylbutyryl-CoA
894.301	C01944	-2.21	Octanoyl-CoA
894.35	C04392	-1.75	P1,P4-Bis(5'-xanthosyl) tetraphosphate; XppppX
895.302	C01944	-2.54	Octanoyl-CoA
895.355	C01944	-2.25	Octanoyl-CoA
895.562	C00100	-4.56	Propanoyl-CoA; Propionyl-CoA; Propionyl coenzyme A
896.229	C01944	-1.96	Octanoyl-CoA
896.304	C01024	-1.79	Hydroxymethylbilane

897.388	C06198	-3.34	P1,P4-Bis(5'-uridyl) tetraphosphate; UppppU
898.154	C01051	-3.29	Uroporphyrinogen III
899.158	C05271	-3.06	trans-Hex-2-enoyl-CoA; (2E)-Hexenoyl-CoA
899.3	C04392	-2.79	P1,P4-Bis(5'-xanthosyl) tetraphosphate; XppppX
